# On piecewise models and species–area patterns

**DOI:** 10.1002/ece3.5417

**Published:** 2019-07-02

**Authors:** De Gao, Zhen Cao, Peng Xu, Gad Perry

**Affiliations:** ^1^ School of Resources and Environmental Science Hebei Normal University Shijiazhuang China; ^2^ Department of Chemical and Environmental Engineering Hebei College of Industry and Technology Shijiazhuang China; ^3^ Department of Mathematics and Statistics Eastern Michigan University Ypsilanti Michigan USA; ^4^ Department of Natural Resources Management Texas Tech University Lubbock Texas USA

**Keywords:** breakpoint values, conservation biogeography, island biogeography, piecewise regression, small island effect, species–area relationships, thresholds, zero‐slope regression

## Abstract

**Aim:**

Area thresholds, at which the form of the species–area relationship (SAR) changes abruptly, have played an important role in the theoretical framework of conservation biogeography and biodiversity research. The application of piecewise regressions has been advocated as a rigorous statistical technique to identify such thresholds within SARs, but a large variety of piecewise models remains untested. We explore the prevalence and number of thresholds in SARs and examine whether the currently widely used method for detecting the small island effect (SIE) is robust.

**Location:**

Global.

**Taxon:**

We consider all multicellular taxa based on the criteria of datasets selection.

**Methods:**

We apply 15 regression models, including linear regression and piecewise regressions with two and three segments to 68 global island datasets that are sourced from the literature.

**Results:**

The number of area thresholds in SARs varied among groups and correlated positively with area range of a studied system. Under the AIC or AIC_c_ criterion, three‐segment piecewise models were more prevalent, whereas under the BIC criterion, two‐segment piecewise models were more prevalent. From the results of Aegean Sea isopods, West Indies herpetofauna, and Australian Islands mammals, we found evidence that the traditional criteria for detection of SIEs are not robust.

**Main conclusions:**

Our study demonstrates that (a) to detect an SIE, the comparison should use as many models as possible, including not only variants with and without a left‐horizontal part, but also those with two and more segments; (b) naive use of the traditional two‐segment piecewise regressions may cause poor estimations of both slope and breakpoint values; (c) the number of thresholds increases with the area range of a studied system; (d) conservation biologists and applied ecologists should determine the number of area thresholds when estimating the precise species–area patterns and making management strategies in fragmented landscapes.

## 1 INTRODUCTION

The species–area relationship (SAR), fundamental to the development of MacArthur and Wilson's (1963, 1967) equilibrium theory and island biogeography, has great utility for assessing species diversity and conservation needs (Ladle & Whittaker, 2011; Lomolino, 2000). Insights from SARs have been applied in optimal reserve design (Neigel, 2003), identifying biodiversity hotspots (Fattorini, 2007), and assessing the impact that habitat fragmentation exerts on diversity (Harcourt & Doherty, 2005). However, debate continues as to the applicability of island biogeographic theory in the context of habitat fragmentation (Laurance, 2008), because as species–area studies began to accumulate, scholars have found evidence for nonlinearity of log‐transformed SARs.

Some studies showing thresholds in SARs depict a steep–shallow relationship, with an initially rapid increase in richness with area until a threshold is reached beyond which a slow rate of increase applies (Drinnan, 2005; Fahrig, 2001; Matthews, Steinbauer, Tzirkalli, Triantis, & Whittaker, 2014). Others depict a shallow–steep relationship, showing an initially slow rate of (or no) increase in richness with increasing area, followed by a steeper phase beyond a threshold, often named the small island effect (SIE; Morrison, 2014; Triantis et al., 2006; Wang, Chen, & Millien, 2018; Wang, Millien, & Ding, 2016). Although around 20 functions have been described for fitting SARs (Triantis, Guilhaumon, & Whittaker, 2012), piecewise regression has been advocated as a rigorous statistical technique suitable for identifying area thresholds (Ficetola & Denoёl, 2009).

Currently, four piecewise regression functions are found in the literature: (a) continuous left‐horizontal function [*Y* = *c* + (log *A* > *T*) *z* (log *A* − *T*) (Lomolino & Weiser, 2001)]; (b) continuous two‐slope function [*Y* = *c* + (log *A* ≤ *T*) *z*_1_ log *A* + (log *A* > *T*) [*z*_1_ *T* + *z*_2_ (log *A* − *T*)] (Dengler, 2010; Morrison & Spiller, 2008)]; (c) discontinuous two‐slope function [*Y* = (log *A* ≤ *T*) (*c*_1_ + *z*_1_ log *A*) + (log *A* > *T*) (*c*_2_ + *z*_2_ log *A*) (Gentile & Argano, 2005)]; and (d) continuous right‐horizontal function [*Y* = *c* + (log *A* ≤ *T*) *z* log *A* + (log *A* > *T*) *z T* (Dengler, 2010)]. Several shortcomings in the applications of piecewise models persist, however.

First, it is conceivable that discontinuous relationships with disconnected SAR segments may exist in nature (Gentile & Argano, 2005; Maron et al., 2012), and their detection in a few datasets most likely signifies important roles for confounding variables that were not included in the models, such as isolation or matrix effects (Crowe, 1979; Levenson, 1981; Matthews et al., 2014). However, discontinuous function is only allowed in the two‐slope method, where two linear segments are fitted onto the data (Gentile & Argano, 2005).

Second, the current continuous models include one segment obtained by regression and the other one obtained by nonregression: slope iteration for the continuous two‐slope function (Dengler, 2010; Morrison & Spiller, 2008) or direct inheritance for the continuous left‐horizontal (Lomolino & Weiser, 2001) and the continuous right‐horizontal (Dengler, 2010) functions. However, either segment could be obtained by regression, that is, if the upper segment is the regression part, the lower segment will be the nonregression one, and vice versa. So, each method actually has two possible expression and calculation forms rather than one, which is neglected in the literature.

Third, piecewise models with two segments have been widely used in previous studies (e.g., Dengler, 2010; Lomolino & Weiser, 2001; Wang et al., 2016). However, Lomolino and Weiser (2001) and Rosenzweig (2004) proposed three biological scales of species–area curves with three corresponding dominant processes of species addition: (a) stochastic extinction forces structure insular communities on small islands; (b) more deterministic, ecological factors associated with habitat diversity, carrying capacity and extinction/immigration dynamics as envisioned by MacArthur and Wilson (1967) on islands of intermediate size; and (c) in situ speciation on relatively large islands. Therefore, limiting our attention to just one or two species–area patterns is unreasonable.

In recent work on detecting the SIE of herpetofauna of the West Indies, Gao and Perry (2016a) showed that piecewise regressions with three segments performed better than those of two segments, suggesting piecewise models with more than two segments should be considered. Dengler (2010) proposed that model comparisons should include at least one variant without an SIE, one with a left‐horizontal function, and one with a two‐slope function. And thereafter, datasets fitted best by the left‐horizontal function are thought to provide stronger evidence for an SIE than those fitted best by the two‐slope function (Morrison, 2014; Wang et al., 2016). However, Dengler's (2010) proposal is based on currently incomprehensive piecewise models, which are restricted to a maximum of two slopes, so we question the reliability of the method for detecting the SIE.

The aim of this paper was thus twofold. First, we provide an updated synthetic analysis of piecewise models to the SARs of 68 island datasets, representing different geographic regions, taxa, and size ranges. We discuss whether the currently widely used method for detecting the SIE is robust when other piecewise models are taken into account. Second, we examine whether larger sample size range and larger species range lead to detection of more area thresholds.

## 2 METHODS

### 2.1 Data collection

Between May 2015 and July 2017, we searched for true and habitat island studies and relevant datasets within four main abstracting databases (JSTOR, ISI Web of Knowledge, and BIOSIS Biological Abstracts) using the keywords “species richness,” “fragments,” “small island effect,” and “islands” in different combinations. Cross‐referenced papers derived from the reference lists of sourced papers were also included. More than a thousand journal papers, books, reports, online databases, and unpublished resources were screened. To increase the statistical power for the robustness of piecewise models, however, we only included datasets that met the following criteria: (a) islands constituted discrete patches of habitat surrounded by contrasting habitat; (b) there were at least 40 islands within each dataset; (c) the area and species richness of each island were accessible either in the source publication or from the authors of the source papers; and (d) the dataset derived from a source did not overlap with those from any other sources (data for different taxa within the same study system were accepted).

For Aegean Sea isopods, we removed the largest island from the dataset and formed a new dataset to facilitate further comparison. Finally, 68 datasets from 38 sources (see Appendix S1 in Supporting Information) were retained for further analyses.

### 2.2 Statistical analyses

**Figure 1:**
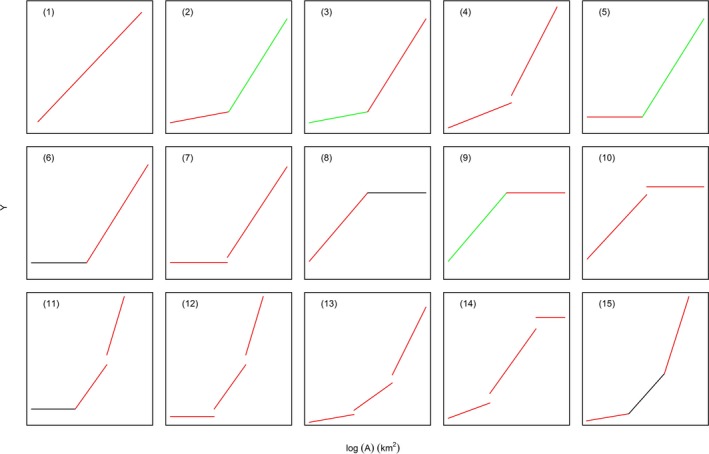
Schematic illustration of the function and derivation of each segment in each model. Red line signifies the segment is obtained by regression; green line signifies segment is obtained by slope iteration; and black line signifies the segment is obtained by direct inheritance. The illustrated slope of each segment (excluding the horizontal segment) in each model does not imply the actual relative shallowness or steepness in data analysis. We denote the *x*‐axis as log (A) (km^2^), and the *y*‐axis as Y, which stands for species richness *S* or log *S* for semi‐log and log–log versions of the model, respectively

We fitted a linear model (Equation 1) alongside 14 piecewise models (Equations (2), (3), (4), (5), (6), (7), (8), (9), (10), (11), (12), (13), (14), (15); Figure 1) to each dataset.

(1) $$Y=c_{1}+z_{1}\text{log}A$$

(2) $$Y=c_{1}+\left(\text{log}A\leq T_{1}\right)z_{1}\text{log}A+\left(\text{log}A>T_{1}\right)\left[z_{1}T_{1}+z_{2}\left(\text{log}A-T_{1}\right)\right]$$

(3) $$Y=c_{1}+\left(\text{log}A\leq T_{1}\right)\left[z_{1}\text{log}A+\left(z_{2}-z_{1}\right)T_{1}\right]+\left(\text{log}A>T_{1}\right)z_{2}\text{log}A$$

(4) $$Y=\left(\text{log}A\leq T_{1}\right)\left(c_{1}+z_{1}\text{log}A\right)+\left(\text{log}A>T_{1}\right)\left(c_{2}+z_{2}\text{log}A\right)$$

(5) $$Y=c_{1}+\left(\text{log}A>T_{1}\right)z_{1}\left(\text{log}A-T_{1}\right)$$

(6) $$Y=c_{1}+\left(\text{log}A\leq T_{1}\right)z_{1}T_{1}+\left(\text{log}A>T_{1}\right)z_{1}\text{log}A$$

(7) $$Y=\left(\text{log}A\leq T_{1}\right)c_{1}+\left(\text{log}A>T_{1}\right)\left(c_{2}+z_{1}\text{log}A\right)$$

(8) $$Y=c_{1}+\left(\text{log}A\leq T_{1}\right)z_{1}\text{log}A+\left(\text{log}A>T_{1}\right)z_{1}T_{1}$$

(9) $$Y=c_{1}+\left(\text{log}A\leq T_{1}\right)z_{1}\left(\text{log}A-T_{1}\right)$$

(10) $$Y=\left(\text{log}A\leq T_{1}\right)\left(c_{1}+z_{1}\text{log}A\right)+\left(\text{log}A>T_{1}\right)c_{2}$$

(11) $$\begin{array}{c} Y=\left(\text{log}A\leq T_{2}\right)\left[c_{1}+\left(\text{log}A\leq T_{1}\right)z_{1}T_{1}+\left(\text{log}A>T_{1}\right)z_{1}\text{log}A\right]\\ +\left(\text{log}A>T_{2}\right)\left(c_{2}+z_{2}\text{log}A\right) \end{array}$$

(12) $$Y=\left(\text{log}A\leq T_{1}\right)c_{1}+\left(\text{log}A>T_{1}\text{AND}\text{log}A\leq T_{2}\right)\left(c_{2}+z_{1}\text{log}A\right)+\left(\text{log}A>T_{2}\right)\left(c_{3}+z_{2}\text{log}A\right)$$

(13) $$\begin{array}{c} Y=\left(\text{log}A\leq T_{1}\right)\left(c_{1}+z_{1}\text{log}A\right)+\left(\text{log}A>T_{1}\text{AND}\text{log}A\leq T_{2}\right)\left(c_{2}+z_{2}\text{log}A\right)\\ +\left(\text{log}A>T_{2}\right)\left(c_{3}+z_{3}\text{log}A\right) \end{array}$$

(14) $$Y=\left(\text{log}A\leq T_{1}\right)\left(c_{1}+z_{1}\text{log}A\right)+\left(\text{log}A>T_{1}\text{AND}\text{log}A\leq T_{2}\right)\left(c_{2}+z_{2}\text{log}A\right)+\left(\text{log}A>T_{2}\right)c_{3}$$

(15) $$\begin{array}{c} Y=\left(\text{log}A\leq T_{1}\right)\left(c_{1}+z_{1}\text{log}A\right)+\left(\text{log}A>T_{1}\text{AND}\text{log}A\leq T_{2}\right)[\left(c_{1}-c_{2}+z_{1}T_{1}-z_{2}T_{2}\right)\left(\text{log}A-T_{1}\right)/\left(T_{1}-T_{2}\right)+c_{1}\\ +z_{1}T_{1}]+\left(\text{log}A>T_{2}\right)\left(c_{2}+z_{2}\text{log}A\right) \end{array}$$

In these equations, *Y* stands for species richness *S* or log *S* for semi‐log and log–log versions of the model, respectively, *A* for area (in the unit of km^2^), while *c_i_* (intercept), *z_i_* (slope), and *T_i_* (breakpoint) are fitted parameters. The logical AND operator combines two logical operands that have a value true or false. The expression combined by logical AND evaluates to true if both operands log *A* > *T*_1_ and log *A* ≤ *T*_2_ evaluate to true; if either or both of the operands for the logical AND operator are false, the result of the expression is false. The logical expressions in brackets return value 1 if they are true and 0 if they are false. Islands that have no species recorded were involved in multiple datasets (see Table S1 in Appendix S1). In some datasets, there were lots of small islands, very few large ones, and a large gap in between. To improve statistical rigor and avoid outlier effects, all datasets, with the exception of the three large datasets mentioned below, were analyzed with the log–log versions of the model. Since removing observations is not favored (Williamson, 1981), we used the traditional log (*S* + 1) for the datasets that includes empty islands, which is consistent with the treatment applied by Suarez, Bolger, and Case (1998), Lavin et al. (2001), and Gao and Perry (2016a). For Aegean Sea isopods, Aegean Sea centipedes, and Italian islands centipedes (see Table S1 in Appendix S1), we used *S* rather than log *S* based on two considerations: (a) Species richness values distribute pretty continuously, and log transformation is unnecessary and (b) both Sfenthourakis and Triantis (2009) and Dengler (2010) have used the untransformed version in their analyses, so we follow this to facilitate the comparison among our results as well as to those of Dengler (2010).

Equations (2), (3), (4), (5), (6), (7), (8), (9), (10) are the comprehensive possible function forms for the two‐segment piecewise method, in which Equations 2 and 3 comprise a regression segment and a slope‐iteration segment; Equation 4 comprises two regression segments; Equations 5 and 9 comprise a zero‐slope regression segment and a slope‐iteration segment; Equations 7 and 10 comprise a regression segment and a zero‐slope regression segment; and Equations 6 and 8 comprise a regression segment and a direct‐inheritance segment (Figure 1). Equations (2), (3), (4), (5), (6), (7), and (8), (9), (10) are the two‐slope, the left‐horizontal, and the right‐horizontal variants, respectively, under the two‐segment condition. As for the three‐segment condition, due to the option of continuity or discontinuity at each breakpoint and performance (regression/slope iteration/direct inheritance) of each segment, there will be many more possible forms than the two‐segment condition. So, here, we just randomly choose five forms (Equations (11), (12), (13), (14), (15)) based on the consideration of computational load. Equations (12), (13), (14) comprise three regression segments, in which, a zero‐slope regression segment constitutes Equations 12 and 14; Equations 11 and 15 comprise two regression segments and a direct‐inheritance segment (Figure 1).

**Figure 2:**
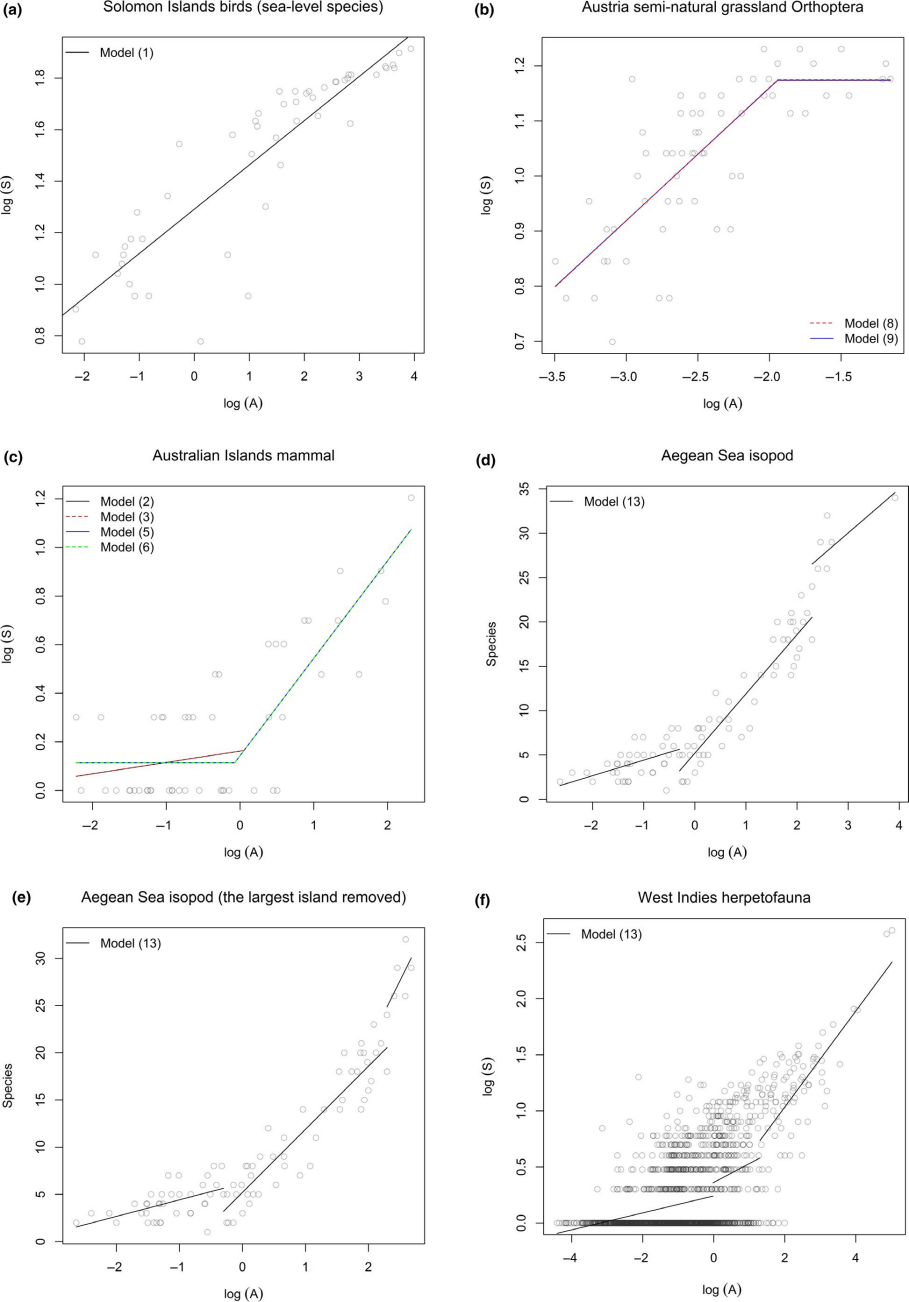
Results of model selection for six sample datasets: (a) Solomon Islands birds (sea‐level species) by BIC, (b) Austria semi‐natural grassland Orthoptera species by BIC, (c) Australian Islands mammals by AIC_c_, (d) and (e) Aegean Sea isopods by AIC_c_, and (f) West Indies herpetofauna by AIC. We analyzed Aegean Sea isopods for both with (d) and without (e) the largest island

If a piecewise model is discontinuous, the model expression will be relatively easier, and each segment can be depicted as (*Y* = *c*) for a zero‐slope regression or (*Y* = *c* + *z* log *A*) for a normal regression. However, if a piecewise model is continuous, the model expression will be more complicated, as the right end of a lower segment has to be connected to the left end of an upper segment. And thus, to unify the expressions, we give priority to the expression of regression segments and denote (*Y* = *c*) for zero‐slope regression segments and (*Y* = *c* + *z* log *A*) for linear regression segments. Therefore, Model 6 (Figure 2; expressed as [*Y* = *c* + (log *A* > *T*) *z* (log *A* − *T*) (Lomolino & Weiser, 2001)]) is denoted here as [*Y* = *c*_1_ + (log *A* ≤ *T*_1_) *z*_1_ *T*_1_ + (log *A* > *T*_1_) *z*_1_ log *A*], because, in this way, *Y* = *c*_1_ + *z*_1_ *T*_1_ when log *A* ≤ *T*_1_ and *Y* = *c*_1_ + *z*_1_ log *A* when log *A* > *T*_1_, and it can be clearly seen that the upper, rather than the lower, part is a regression segment. Instead, the function provided by Lomolino and Weiser (2001) is consistent with Equation 5 here, denoting a lower zero‐slope regression segment and an upper slope‐iteration segment (Figure 1).

We used a minimum residual sum of squares (RSS) method (Gao & Perry, 2016a) to estimate the threshold values. For Equations (2), (3), (4), (5), (6), (7), (8), (9), (10), (11), (12), (13), (14), (15), the parameters were estimated by using nonlinear estimation procedures based on iteration. If a piecewise model is continuous at a breakpoint, then the breakpoint lying between two adjacent data points will influence the RSS value of the model, we incremented such breakpoint values (*T*) by 0.001; however, if a piecewise model is discontinuous at a breakpoint, then the breakpoint lying between two adjacent data points will not influence the RSS value of the model, so we assigned the breakpoint values (*T*) to the log‐transformed area values of each island (see Fig. S2–S10 in Appendix S2). Equations (12), (13), (14) are discontinuous, so we assigned the first breakpoint values (*T*_1_) to the log‐transformed area values of each island, and at any particular value of *T*_1_, *T*_2_ was assigned to the log‐transformed area values between *T*_1_ and the maximum log‐transformed area value. We recorded the minimum RSS value produced by the iteration of *T*_2_ for each particular value of *T*_1_, so that the first breakpoint (*T*_1_) was determined prior to the second one (*T*_2_). After *T*_1_ was determined, we run iteration of *T*_2_ again to look for the *T*_2_ that produced the minimum RSS value (see Fig. S12–S14 in Appendix S2). For Equations 11 and 15, we assigned the second breakpoint values (*T*_2_) to the log‐transformed area values of each island or incremented *T*_2_ by 0.001 for Equations 11 and 15, respectively. And *T*_1_ was incremented from the minimum log‐transformed area value to *T*_2_ by 0.001. We recorded the minimum RSS value produced by the iteration of *T*_1_ for each particular value of *T*_2_, so that the second breakpoint (*T*_2_) was determined prior to the first one (*T*_1_). After *T*_2_ was determined, we run iteration of *T*_1_ again to look for the *T*_1_ that produced the minimum RSS value (see Fig. S11; Fig. S15 in Appendix S2).

Both the Akaike's information criterion (AIC; AIC was applied for Worldwide terrestrial mammals and West Indies herpetofauna and Akaike's information criterion corrected (AIC_c_) for the other taxonomic groups; Burnham & Anderson, 2002) and the Bayesian information criterion (BIC) were applied, respectively, for each taxonomic group as criterions for model selection (Burnham & Anderson, 2002). For each model in each taxonomic group, we calculated the log‐likelihood (log *L*), which was used to determine AIC or AIC_c_ (hereafter referred to as AIC_(c)_ for brevity), and BIC, respectively. We also calculated the difference in AIC_(c)_ (∆AIC_(c)_) and BIC (∆BIC) for the model selection. The model with lower AIC_(c)_ or BIC (hereafter referred to as IC for brevity) value indicates stronger evidence for being better over the others. Models with ICs less than two apart (∆IC ≤ 2) are more or less equivalent (equally supported); those with ICs four to seven apart are clearly distinguishable; and models with ICs more than 10 apart are definitely different (Burnham & Anderson, 2002).

We evaluated the existence of an SIE within an island system by comparing the fit of a linear model (Equation 1) with the other Equations ((2), (3), (4), (5), (6), (7), (8), (9), (10), (11), (12), (13), (14), (15)). When either of Equations (2), (3), (4), (5), (6), (7), (8), (9), (10), (11), (12), (13), (14), (15) were supported as the best model (∆IC ≤ 2), an SIE was considered to be present (Lomolino & Weiser, 2001). Equations 5, 6, 7 11, and 12 being the best model is considered to provide stronger support for the SIE than Equations 2, 3, 4, 13, 14, and 15 (Morrison, 2014; Wang et al., 2016). We evaluated the existence of a steep–shallow area threshold by comparing the fit of a linear model (Equation 1) with Equations 8, 9, 10, and 14. If Equations 8, 9, 10, or 14 are supported (∆IC ≤ 2), the evidence of a steep–shallow area threshold is found (Matthews et al., 2014). We evaluated the threshold number of a dataset by comparing all the equations. Equation 1 has zero threshold, Equations (2), (3), (4), (5), (6), (7), (8), (9), (10) one threshold, and Equations (11), (12), (13), (14), (15) two thresholds. We depicted the threshold number of a dataset as the average number of thresholds represented by all corresponding models that had equal support (∆IC ≤ 2). For example, if Equation 1 is supported, the threshold number is calculated as 0/1 = 0; if Equations 1, 2, and 11 are supported, the threshold number is calculated as (0 + 1+2)/3 = 1. We calculated area range as log *A*_(max)_ − log *A*_(min)_, which reflected how many orders of magnitude that island size varies across a studied system. We applied the log‐transformed version of species range, calculated as log (*S*_(max)_ − *S*_(min)_), to avoid outlier effects and improve linearity. Last, we ran linear regression to show the relationship between threshold number and area range as well as the relationship between threshold number and species range, using AIC_(c)_ and BIC for model selection, respectively. We performed all analyses using R 3.1.1 (R Development Core Team, 2014).

## 3 RESULTS

Among the 68 global island datasets, SIE thresholds were detected in 58 cases (85%) and 46 cases (68%) using AIC_(c)_ and BIC, respectively, whereas steep–shallow thresholds were detected in 31 cases (46%) and 19 cases (28%) using AIC_(c)_ and BIC, respectively (Table 1). Among the 58 cases in which SIE thresholds were detected, two‐segment piecewise models were supported in 35 cases (60%) and three‐segment piecewise models were supported in 42 cases (72%) under AIC_(c)_ criterion, whereas under BIC criterion, two‐segment piecewise models were supported in 36 cases (78%) and three‐segment piecewise models were supported in 16 cases (35%) among the 46 cases in which SIE thresholds were detected (Table 1). Among the 31 cases in which steep–shallow thresholds were detected, two‐segment piecewise models were supported in 11 cases (35%) and three‐segment piecewise models were supported in 26 cases (84%) under AIC_(c)_ criterion, whereas under BIC criterion, two‐segment piecewise models were supported in 13 cases (68%) and three‐segment piecewise models were supported in six cases (32%) among the 19 cases in which steep–shallow thresholds were detected (Table 1).

**Table 1:** The distribution of occurrence frequency across model segments and threshold forms determined under AIC_(c)_ and BIC model selection criterions, respectively, for the 68 global datasets. In our study, models with ∆IC ≤2 were equally supported, and therefore, the sum of the numbers did not equal 68 (total sample size)

Model segments	AIC_(c)_			BIC		
	SIE threshold	Steep–shallow threshold	None threshold	SIE threshold	Steep–shallow threshold	None threshold
Linear model	—	—	12	—	—	31
Two‐segment models	35	11	—	36	13	—
Three‐segment models	42	26	—	16	6	—
Total cases	58	31	12	46	19	31

The number of area thresholds in SARs varied among groups (Figure 2; Table S2 in Appendix S1). Model selection based on BIC identified Equation 1 as the most parsimonious model for Solomon Islands birds (sea‐level species; Figure 2a); Equations 8 and 9 as equally the most parsimonious models for Austria semi‐natural grassland Orthoptera species (Figure 2b). Model selection based on AIC_(c)_ identified Equations 2, 3, 5, and 6 as the most parsimonious model for Australian Islands mammals (Figure 2c); and Equation 13 as the most parsimonious model for Aegean Sea isopods (Figure 2d) and West Indies herpetofauna (Figure 2f). Moreover, different groups showed different forms of the SAR with increasing area: steep–shallow, shallow–steep, shallow–steep–shallow, and shallow–steep–steeper relationships for Austria semi‐natural grassland Orthoptera species (Figure 2b), Australian Islands mammals (Figure 2c), Aegean Sea isopods (Figure 2d), and West Indies herpetofauna (Figure 2f), respectively.

We compared the first breakpoint value (*T*_1_) between the best performing three‐segment method and the best performing two‐segment method for Aegean Sea isopods and West Indies herpetofauna, and found Equations 2, 3, and 4 were equally the most parsimonious models (mutual *∆*AIC_(c)_ ≤2) for the two‐segment method; moreover, *T*_1_ estimated by Equations 2, 3, and 4 were larger than by Equation 13 for Aegean Sea isopods (0.227–0.914 vs. −0.301; Figure 2d; Table S2 in Appendix S1) but smaller for West Indies herpetofauna (−0.603–‐0.111 vs. −0.022; Figure 2f; Table S2 in Appendix S1).

We examined whether the traditional method of detecting the SIE is still robust here for Australian Islands mammals, Aegean Sea isopods, and West Indies herpetofauna using AIC_(c)_ as the criterion for model selection, which is widely used in other SIE studies (e.g., Morrison, 2014; Wang et al., 2016). For Aegean Sea isopods and West Indies herpetofauna, any choice of Equations 2, 3, 4 (one threshold without a left‐horizontal segment) were better than Equations 5, 6, 7 (one threshold with a left‐horizontal segment) but worse than Equation 11 (two thresholds with a left‐horizontal segment); however, Equation 11 was worse than Equation 13 (two thresholds without a left‐horizontal segment; Table S2 in Appendix S1). For Australian Islands mammals, if we compare Equation 4 with Equation 5 or 6, no slope for the SIE will be the conclusion; however, if we compare Equation 2 or 3 with Equation 5, 6, or 7, it is ambiguous to say whether there is a slope (Table S2 in Appendix S1). Thereby, the traditional method of detecting the SIE is not robust, and different choices of model comparison can lead to totally different conclusions.

**Figure 3:**
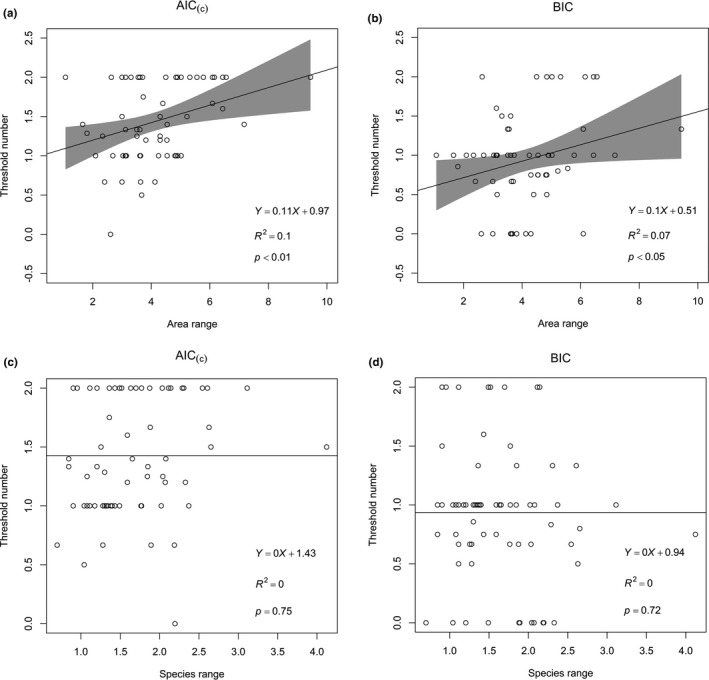
Linear regression, showing the relationship between threshold number and log_10_‐transformed area range (a and b) and species range (c and d) of a studied system, using AIC_(c)_ and BIC for model selection, respectively

From the result of linear regressions conducted by using 68 island datasets, a significant positive relationship was found between threshold number and area range under both AIC_(c)_ (*p* < 0.01) criterion and BIC criterion (*p* < 0.05; Figure 3). This result suggests more species–area patterns could be detected as the area distributions of sampled islands get broader. However, no obvious relationship was found between threshold number and species range (Figure 3).

## 4 DISCUSSION

Our results imply both SIE thresholds (58 cases, 85% and 46 cases, 68% under AIC_(c)_ and BIC criterions, respectively) and steep–shallow thresholds (31 cases, 46% and 19 cases, 28% under AIC_(c)_ and BIC criterions, respectively) are common in nature. Under AIC_(c)_ criterion, three‐segment piecewise models are more prevalent (42 cases, 72% and 26 cases, 84% in detecting SIE and steep–shallow thresholds, respectively), whereas under BIC criterion, two‐segment piecewise models are more prevalent (36 cases, 78% and 13 cases, 68% in detecting SIE and steep–shallow thresholds, respectively). Two‐segment piecewise models have been widely used in previous studies (e.g., Lomolino & Weiser, 2001; Dengler, 2010; Wang et al., 2016); however, three‐segment piecewise models, despite their prevalent existence, have long been neglected.

The shallow–steep–shallow relationship for Aegean Sea isopods (Figure 2d) is inconsistent with the result of Dengler (2010) who detected only two segments; however, the three‐segment models have lower AIC_c_ values than those of two‐segment models. The first two SAR patterns (shallow–steep) are similar to those of Australian Islands mammals, and two possible reasons may explain the last pattern (shallow). First, larger islands normally possess larger human population and richer natural resources, so that a more advanced economic development could be expected (Gao & Perry, 2016b; Helmus, Mahler, & Losos, 2014). Thus, human‐mediated dispersal among those large islands could be facilitated and thereby lowering the slope of SAR. Second, species richness of isopods might be seriously underestimated for the largest island, because if the largest island is removed, a shallow–steep–steeper relationship is obtained (Figure 2e). The shallow–steep–steeper relationships of Aegean Sea isopods (the largest island removed; Figure 2e) and West Indies herpetofauna are in accordance with the proposal that put forward by Lomolino and Weiser (2001) and Rosenzweig (2004), which states three biological scales of species–area curve with three corresponding dominant processes of species addition and slope ranges.

Besides, other factors such as regional biogeographical setting (Gerstner, Dormann, Václavík, Kreft, & Seppelt, 2014), historical events (Hewitt, 2000; Kisel, McInnes, Toomey, & Orme, 2011), climate change (Lewis, 2006), human activities (Corlett, 2015; Gao & Perry, 2016b), and fire and other ecological disturbances (Blondel & Vigne, 1993) may also be responsible for the variation among organisms in their response to a particular physical setting, sensitivity to barriers to dispersal, and habitat extent requirement for proliferation and diversification (Blondel & Vigne, 1993; Reyjol et al., 2007). All these factors, acting either additively or synergistically, may influence the proposed species–area patterns across portion or the whole scale. Thereby, the larger size range with a larger number of sampled islands, the more detailed factors shaping the patterns could be depicted, and the more kind of SARs might be detected. Thus, the number of thresholds is positively correlated with the area range of a studied system, which is consistent with Matthews, Triantis, et al. (2016) and Wang et al. (2016). In contrast, species range received considerably less support in determining the number of thresholds. The main reason for the weak relationships is probably that species range is less effective in determining the limits of the SIE and evolutionary processes considering different taxonomic group, island age, and human influence among the studied systems and is less likely to correlate to area range.

The currently widely used method for detecting the SIE is not robust. First, although a two‐segment left‐horizontal function performs better than a two‐slope function, it may be worse than a three‐slope function; and/or although a two‐slope function performs better than a two‐segment left‐horizontal function, it may be worse than a three‐segment left‐horizontal function (e.g., Aegean Sea isopods; West Indies herpetofauna). Second, even if under the two‐segment circumstances, different choices of comparison between two models with and without a left‐horizontal part may lead to totally different conclusions (e.g., Australian Islands mammals). It implies that the currently available but incomprehensive piecewise models may provide a biased result in detecting the SIE. Therefore, we suggest previous SIE detection works conducted using an incomprehensive set of piecewise models, while ignoring the possibility of three segments need to be reanalyzed. It is very important to do so because the threshold value, where the slope changes, may be important for a successful application of island theory to conservation biogeography (Triantis & Bhagwat, 2011). However, the incomprehensive set of piecewise models may provide a misleading result on the existence of an SIE; moreover, the traditional two‐segment method may cause poor estimations for both slope and threshold value of an SIE. In order to improve the method for detecting the SIE, we suggest that it is essential to determine the number of area thresholds and apply a comprehensive set of piecewise models rather than just as few as two. We agree with the suggestions that a large sample size and small or even empty islands should be included in analyzing the SAR of a system (Matthews et al., 2014; Wang et al., 2016). For instance, if some more large islands (e.g., Australia) were included into Australian Islands mammals, a third species–area pattern with a higher slope might appear; and if some more small empty islands were included into Austrian semi‐natural grassland Orthoptera species, an SIE might appear. Besides, more precise threshold values could be obtained if a complete sampling effort was taken and a large sample size was included.

Although the three‐segment piecewise regressions could display two area thresholds proposed by Lomolino and Weiser (2001) and Rosenzweig (2004) and may provide better estimations for slope and threshold value within an SIE, they incorporate more parameters into the models, which in turn will decrease the statistical power for the robustness as compared with the two‐segment regressions using the same data size. Therefore, a larger number of surveyed islands in the dataset are required for the applicability of three‐segment approach. To ensure an adequate island number incorporated in the dataset, we suggest, first, empty islands from a smaller spatial scale are welcomed to be collected because the effect of empty islands in generating SIEs is prevalent (Wang et al., 2016). Second, an incomplete sampling effort is usually taken, leaving many other islands in the studied system uninvestigated (e.g., Davies & Smith, 1998; Panitsa, Tzanoudakis, Triantis, & Sfenthourakis, 2006; Wang, Bao, Yu, Xu, & Ding, 2010). However, the applicability of complicated models declines from relatively complete island lists to extremely incomplete ones, so we suggest island collection is ought to be complete at the current studied spatial scale to allow more complex models tested. Third, sometimes a surveyed location is involved in a larger‐scale system, for instance, Bahamas, where Morrison (2014) studied the SIE of plants, is actually a portion of the whole West Indies region. The regional SAR patterns could give insight into the causes of local patterns (Schrader, Moeljono, Keppel, & Kreft, 2019), so islands collected from a larger spatial scale are also suggested.

Determining the number of area thresholds in a system is critical to estimating the precise species–area patterns. Thus, we suggest previous piecewise regression and SIE detection works need to be reanalyzed. Two thresholds rather than one shine new light on conservation biogeography: First, it offers opportunity to assess variables such as habitat diversity, productivity, island age, energy, and environmental heterogeneity (Anderson & Wait, 2001; Tjørve & Tjørve, 2011; Whitehead & Jones, 1969) within the limits of the first threshold value; second, speciation may be the dominant process adding to the species richness of assemblages beyond the limits of the second threshold value (Losos & Schluter, 2000); third, habitat diversity, carrying capacity, and extinction/immigration dynamics as envisioned by MacArthur and Wilson (1967) may govern species richness between the first and second threshold values; fourth, it appreciates other factors, such as historical events (Hewitt, 2000; Kisel et al., 2011), climate change (Lewis, 2006), human activities (Corlett, 2015; Gao & Perry, 2016b), and so on, to exert their influence across portion or the whole scale; and last, the rate of biodiversity loss from habitat loss varies among three area intervals, and thus, two thresholds can effectively facilitate the establishment of protection priority level among islands and maximize the diversity saved in a system with limited funds for conservation. We conclude that conservation biologists and applied ecologists should determine the number of area thresholds when estimating the precise species–area patterns and making management strategies in fragmented landscapes.

Despite the widespread use of the AIC, some believe that it is too liberal and tends to select overly complex models (Kass & Raftery, 1995). As compared to the AIC, the BIC is more conservative, insisting on a greater improvement in fit before it will accept a more complex model (Link & Barker, 2006). That is the reason why in our results three‐segment piecewise models are more prevalent under AIC_(c)_ criterion in finding SIE and steep–shallow thresholds, as well as the lower slope and less significant *p*‐value of the relationship between threshold number and area range under BIC criterion. However, the merits and debits of AIC and BIC remain controversial, and a formal comparison in terms of performance between AIC and BIC is very difficult (cf. Burnham & Anderson, 2002, pp. 293–305, for a pro‐AIC account, and Kass & Raftery, 1995, for a pro‐BIC account), so we used both criteria rather than one. To date, although there are a number of species–area pattern studies, the AIC was dominantly applied. Our study is among the first attempt to apply both criterions for model selection in this field. Besides, previous studies concerning SARs and SIEs comprised a large portion of island‐poor (<20 islands) archipelagoes in the analyses (e.g., Matthews, Guilhaumon, Triantis, Borregaard, & Whittaker, 2016; Triantis et al., 2012; Wang et al., 2016). Instead, to meet the complexity of the piecewise regression models, we selected island datasets with > 40 islands in each case. Our study on piecewise models and species–area patterns thus provide reliable results, contributing to the recent heated disputes on the SIE theory and expanding our horizons on SAR thresholds at different spatial scales.

## CONFLICT OF INTEREST

The authors have no conflict of interest related to this work.

## AUTHOR CONTRIBUTIONS

DG and ZC conceived the idea and collected island datasets; DG, ZC, and PX performed data analyses; DG and GP wrote the first version of the manuscript; and all authors contributed and approved the final version.

## Data Availability

All datasets used in this study are sourced from the literature which can be found in Supporting information.
