# Supplementary material for: On piecewise models and species–area patterns
Source: Ecol Evol. 2019 Jul 2;9(14):8351–61. doi: 10.1002/ece3.5417 (PMC6662316; doi:10.1002/ece3.5417)
Supplement: Supplementary file 1 [file ECE3-9-8351-s001.pdf]

Ecology and Evolution

SUPPORTING INFORMATION

On piecewise models and species–area patterns

De Gao, Zhen Cao, Peng Xu, Gad Perry

Appendix S1 Supplementary results, including Table S1–S2.

Table S1. Properties of the 68 island datasets used in the analyses. S_{min}: minimum species richness. S_{max}: maximum species richness. A_{min}: minimum island size. A_{max}: maximum island size.

Sources	Geographical location	Island type	Taxonomic group	No. of islands	S_{min}	S_{max}	A_{min} (km^2)	A_{max} (km^2)
Abbott & Black (1980)	Western Australia	Continental-shelf	Plants	78	0	105	<0.001	0.19
Ackerman *et al*. (2007)	West Indies, Caribbean	Continental-shelf	Plants (orchid species)	49	3	352	21	105007
Benchimol & Peres (2015)	Balbina Hydroelectric Reservoir, Brazilian Amazonia	Inland	Plants	75	14	73	0.01	16.90
Brown & Hutchings (1997)	Central Amazon	Inland	Butterflies (Total species)	42	88	242	0.01	10

Buckley (1985)	Prince Charlotte, N.E Australia	Continental-shelf	Plants (mangrove species)	61	0	8	<0.001	0.004
Buckley (1985)	Prince Charlotte, N.E Australia	Continental-shelf	Plants (ridge species)	61	0	26	<0.001	0.004
Buckley (1985)	Prince Charlotte, N.E Australia	Continental-shelf	Plants (saltflat species)	61	1	8	<0.001	0.004
Caujapé-Castells *et al*. (2010)	Worldwide	Oceanic	Plants (CR endemic)	62	0	213	39.3	785753
Caujapé-Castells *et al*. (2010)	Worldwide	Oceanic	Plants (EN endemic)	62	0	110	39.3	785753
Caujapé-Castells *et al*. (2010)	Worldwide	Oceanic	Plants (Total endemic)	62	0	13250	39.3	785753
Cody (2006)	Barkley Sound Islands (E), Vancouver	Continental-shelf	Plants	213	0	139	<0.001	1.15
Corke (1992)	Grenadine Islands, Caribbean	Continental-shelf	Amphibia and reptiles	48	0	12	0.01	47
Corke (1992)	Winward Islands, Caribbean	Continental-shelf	Amphibia and reptiles	52	0	20	1	1079
Davies & Smith (1998)	West Indies, Caribbean	Continental-shelf	Butterfly	68	8	202	0.3	108660

Dennis & Shreeve (1997)	British islands	Continental-shelf	Butterfly	73	3	42	0.06	2050.62
Diamond & Mayr (1976)	Solomon Islands, Pacific Ocean	Continental-shelf	Birds (Mountain species)	50	0	23	0.007	8590.991
Diamond & Mayr (1976)	Solomon Islands, Pacific Ocean	Continental-shelf	Birds (Sea-level species)	50	6	82	0.007	8590.991
Dickman (1987)	Urban habitat patches, Oxford City	Inland	Mammals	50	2	17	0.0016	0.2
Essl & Dirnböck (2012)	Austria semi-natural grassland remnants	Inland	Orthoptera species	60	5	17	<0.001	0.07
Flannery (1995)	South-West Pacific and Moluccan Islands	Continental-shelf	Mammals	208	1	44	0.25	150718
Hannus & von Numers (2008)	Brunskär Islands, S.W. Finland	Continental-shelf	Plants	82	10	245	<0.001	0.683
Hannus & von Numers (2008)	Getskär Islands, S.W. Finland	Continental-shelf	Plants	78	1	204	<0.001	0.143
Hogg *et al*. (1989)	Great Lakes, Canada	Continental-shelf	Plants	77	8	433	0.002	49.7
Hu *et al*. (2012)	Thousand Island Lake, China	Inland	Plants	152	25	143	<0.001	1.308
Kohn & Walsh (1994)	North Great Britain	Continental-shelf	Plants	47	0	71	<0.001	0.9958

Lavin *et al*. (2001)	Various islands	Continental-shelf	Plants (Endemic taxa)	64	0	450	5	828800
Lawesson *et al*. (1998)	Danish beech forests	Inland	Plants (All species)	62	35	192	0.011	4.457
Lawlor (1986)	Worldwide	Continental-shelf & Oceanic	Mammals	404	0	120	0.05	743244
Levenson (1981)	Wisconsin Forest, USA	Inland	Plants (canopy species)	43	2	18	<0.001	0.3996
Levenson (1981)	Wisconsin Forest, USA	Inland	Plants (shrub species)	43	8	35	<0.001	0.3996
Levenson (1981)	Wisconsin Forest, USA	Inland	Plants (total woody species)	43	17	42	<0.001	0.3996
Lomba *et al*. (2013)	Forest patches, Portugal	Inland	Plants (Total species)	50	20	68	26.24	309.07
Loyn (1987)	Victorian forests	Inland	Birds (All species)	56	8	67	0.001	17.71
Morrison (2014)	Abaco archipelago, Bahamas	Continental-shelf	Plants (2012)	41	0	8	<0.001	0.00017
Morrison (2014)	Andros archipelago, Bahamas	Continental-shelf	Plants (1999)	58	0	22	<0.001	0.0047
Morrison (2014)	Andros archipelago, Bahamas	Continental-shelf	Plants (2012)	58	0	21	<0.001	0.0047
Morrison (2014)	Exuma Cays archipelago, Bahamas	Continental-shelf	Plants (2012)	144	0	24	<0.001	0.0112

Niemelä *et al*. (1987)	Southern Fennoscandia	Continental-shelf & Inland	Carabid beetles	54	1	24	<0.001	1.6
Nikolić *et al*. (2008)	Adriatic islands	Continental-shelf	Plants (Total species)	106	3	1300	0.0052	409.93
Panitsa *et al*. (2006)	Aegean Sea, Greece	Continental-shelf	Plants (Gramineae)	86	0	18	<0.001	0.5
Panitsa *et al*. (2006)	Aegean Sea, Greece	Continental-shelf	Plants (halophytes)	86	1	14	<0.001	0.5
Panitsa *et al*. (2006)	Aegean Sea, Greece	Continental-shelf	Plants (Leguminosae)	86	0	16	<0.001	0.5
Panitsa *et al*. (2006)	Aegean Sea, Greece	Continental-shelf	Plants (therophytes)	86	0	75	<0.001	0.5
Panitsa *et al*. (2006)	Aegean Sea, Greece	Continental-shelf	Plants (Total species)	86	1	109	<0.001	0.5
Powell & Henderson (2012); Gao & Perry (2016 a, b)	West Indies, Caribbean	Continental-shelf	Herpetofaunas	1668	0	407	<0.001	105457.3
Reed (1981)	British Islands	Continental-shelf	Birds (Breeding landbird species)	73	1	79	0.008	106.922
Rusterholz & Howe (1979)	Burntside Lake, Minnesota	Inland	Birds	56	2	21	<0.001	0.173
Scanlan (1981)	Alexandria Forest, USA	Inland	Plants	66	12	82	0.0024	7.7522
Scanlan (1981)	Minnesota Forest, USA	Inland	Plants	44	13	58	0.0024	0.1089

Sfenthourakis & Triantis (2009; the largest island removed)	Aegean Sea, Greece	Continental-shelf	Isopods (Terrestrial)	89	1	32	0.0023	477.942
Sfenthourakis & Triantis (2009)	Aegean Sea, Greece	Continental-shelf	Isopods (Terrestrial)	90	1	34	0.0023	8261.183
Simaiakis *et al*. (2012)	Aegean Sea	Continental-shelf	Centipedes (Myriapoda: Chilopoda)	68	2	41	0.003	8264.618
Simaiakis *et al*. (2012)	Italian islands	Continental-shelf	Centipedes (Myriapoda: Chilopoda)	88	1	51	0.002	25662
Suarez *et al*. (1998)	California urban, USA	Inland	Invertebrate (Ants)	40	1	20	0.004	1.0161
Vignoli *et al*. (2009)	Rome metropolis	Inland	Amphibians	62	0	8	0.0113	15.923
Vignoli *et al*. (2009)	Rome metropolis	Inland	Reptiles	62	0	13	0.0113	15.923
Wang *et al*. (2010)	Thousand Island Lake, China	Inland	Birds	42	15	73	0.003	12.8923
Wang *et al*. (2010)	Thousand Island Lake, China	Inland	Lizards	42	0	5	0.003	12.8923
Wang *et al*. (2015)	Thousand Island Lake, China	Inland	Snakes	48	0	12	<0.001	12.8923

Welter-Schultes & Williams (1999)	Aegean Sea, Greece	Continental-shelf	Land snails (Total species)	65	5	135	0.0058	8260
Woinarski *et al*. (1999a)	N. Australian Islands	Continental-shelf	Mammals (Terrestrial)	49	0	15	0.006	209.73
Woinarski *et al*. (1999a)	N. Australian Islands	Continental-shelf	Mammals (Bats)	49	0	7	0.006	209.73
Woinarski *et al*. (1999b)	N. Australian Islands	Continental-shelf	Frogs	60	0	8	0.003	209.73
Woinarski *et al*. (1999b)	N. Australian Islands	Continental-shelf	Lizards	60	0	19	0.003	209.73
Woinarski *et al*. (1999b)	N. Australian Islands	Continental-shelf	Reptiles	60	0	27	0.003	209.73
Woinarski *et al*. (1999b)	N. Australian Islands	Continental-shelf	Snakes	60	0	8	0.003	209.73
Woinarski *et al*. (2000)	N. Australian Islands	Continental-shelf	Plants (Introduced)	57	0	9	0.002	209.73
Woinarski *et al*. (2000)	N. Australian Islands	Continental-shelf	Plants (Threatened)	57	0	11	0.002	209.73

DATA SOURCES USED IN THIS STUDY

Abbott, I. & Black, R. (1980) Changes in species composition of floras on islets near Perth, Western Australia. *Journal of Biogeography*, **7**, 399–410.

Ackerman, J.D., Trejo-Torres, J.C. & Crespo-Chuy, Y. (2007) Orchids of the West Indies: predictability of diversity and endemism. *Journal of Biogeography*, **34**, 779–786.

Buckley, R.C. (1985) Distinguishing the effects of area and habitat type on island plant species richness by separating floristic elements and substrate types and controlling for island isolation. *Journal of Biogeography*, **12**, 527–535.

Caujapé-Castells, J., Tye, A., Crawford, D.J., Santos-Guerra, A., Sakai, A., Beaver, K., Lobin, W., Florens, F.B.V., Moura, M., Jardim,R. & Küffer, C. (2010) Conservation of oceanic island floras: present and future global challenges. *Perspectives in Plant Ecology, Evolution, and Systematics*, **12**, 107–129.

Cody, M.L. (2006) Plants on islands: diversity and dynamics on a continental archipelago. University of California Press, Berkley and Los Angeles, CA.

Corke, D. (1992) The status and conservation needs of the terrestrial herpetofauna of theWindward Islands (West Indies). *Biological Conservation*, **62**, 47–58.

Davies, N. & Smith, D.S. (1998) Munroe revisited: a survey of West Indian butterfly faunas and their species-area relationship. *Global Ecology and Biogeography*, **7**, 285–294.

Dennis, R.L.H. & Shreeve, T.G. (1997) Diversity of butterflies on British islands: ecological influences underlying the roles of area, isolation and the size of the faunal source. *Biological Journal of the Linnean Society*, **60**, 257–275.

Diamond, J.M. & Mayr, E. (1976) Species-area relation for birds of Solomon Archipelago. *Proceedings of the National Academy of Sciences USA*, **73**, 262–266.

Essl, F. & Dirnböck, T. (2012) What determines Orthoptera species distribution and richness in temperate semi-natural dry grassland remnants? *Biodiversity and Conservation*, **21**, 2525–2537.

Flannery, T. (1995) Mammals of the South-West Pacific and Moluccan Islands. Cornell University Press, Ithaca, NY.

Gao, D. & Perry, G. (2016 a) Detecting the small island effect and nestedness of herpetofauna of the West Indies. *Ecology and Evolution*, **6**, 5390–5403.

Gao, D. & Perry, G. (2016 b) Species–area relationships and additive partitioning of diversity of native and nonnative herpetofauna of the West Indies. *Ecology and Evolution*, **6**, 7742–7762.

Hannus, J.J. & von Numers, M. (2008) Vascular plant species richness in relation to habitat diversity and island area in the Finnish Archipelago. *Journal of Biogeography*, **35**, 1077–1086.

Hogg, E. H., Mortonj, K. & Vennj, M. (1989) Biogeography of island floras in the Great Lakes. I. Species richness and composition in relation to gull nesting activities. *Canadian Journal of Botany*, **67**, 961–969.

Hu, G., Wu, J., Feeley, K.J., Xu, G. & Yu, M. (2012) The effects of landscape variables on the species-area relationship during late-stage habitat fragmentation. *PLOS ONE*, **7**, e43894.

Kohn, D.D. & Walsh, D.M. (1994) Plant species richness – the effect of island size and habitat diversity. *Journal of Ecology*, **82**, 367–377.

Lavin, M., Wojciechowski, M.F., Richman, A., Rotella, J., Sanderson, M.J. & Matos, A.B. (2001) Identifying tertiary radiations of Fabaceae in the Greater Antilles: alternatives to cladistic vicariance analysis. *International Journal of Plant Sciences*, **162**, S53–S76.

Lawesson, J.E., de Blust, G., Grashof, C., Firbank, L., Honnay, O., Hermy, M., Hobitz, P. & Jensen, L.M. (1998) Species diversity and area–relationships in Danish beech forests. *Forest Ecology and Management*, **106**, 235–245.

Lawlor, T.E. (1986) Comparative biogeography of mammals on islands. *Biological Journal of the Linnean Society*, **28**, 99–125.

Levenson, J.B. (1981) Woodlots as biogeographic islands in southern Wisconsin. In R.L. Burgess & D.M. (Eds.), *Sharpe Forest island dynamics in man-dominated landscapes* (pp. 13–39). Springer-Verlag, New York.

Loyn, R.H. (1987) Effects of patch area and habitat on bird abundances, species numbers and tree health in fragmented Victorian forests. In D.A. Saunders, G.W. Arnold, A.A. Burbridge & A.J.M. Hopkins (Eds.), *Nature conservation: the role of remnants of native vegetation* (pp. 65–77). Surrey Beatty and Sons, Chipping Norton.

Morrison, L.W. (2014) The small-island effect: empty islands, temporal variability and the importance of species composition. *Journal of Biogeography*, **41**, 1007–1017.

Niemelä, J., Haila, Y., Ranta, E., Tiainen, J., Vepsäläinen, K. & Ås, S. (1987) Distribution of carabid beetles in four boreal archipelagoes. *Annales Zoologici Fennici*, **24**, 89–100.

Nikolić, T., Antonić, O., Alegro ,A.L., Dobrović, I., Bogdanović, S., Liber, Z. & Rešetnik, I. (2008) Plant species diversity of Adriatic islands: An introductory survey. *Plant Biosystems*, **142**, 435–445.

Panitsa, M., Tzanoudakis, D., Triantis, K.A. & Sfenthourakis, S. (2006) Patterns of species richness on very small islands: the plants of the Aegean archipelago. *Journal of Biogeography*, **33**, 1223–1234.

Powell, R. & Henderson, R.W. (2012) Island lists of West Indian amphibians and reptiles. *Bulletin of the Florida Museum of Natural History*, **51**, 85–166.

Reed, T. (1981) The number of breeding land-bird species on British Islands. *Journal of Animal Ecology*, **50**, 613–624.

Scanlan, M.J. (1981) Biogeography of forest plants in the prairie-forest ecotone in western Minnesota. In R.L. Burgess & D.M. (Eds.), *Sharpe Forest island dynamics in man-dominated landscapes* (pp. 13–39). Springer-Verlag, New York.

Sfenthourakis, S. & Triantis K.A. (2009) Habitat diversity, ecological requirements of species and the Small Island Effect. *Diversity and Distributions*, **15**, 131–140.

Simaiakis, S.M., Tjørve, E., Gentile, G., Minelli, A. & Mylonas, M. (2012) The species–area relationship in centipedes (Myriapoda: Chilopoda): A comparison between Mediterranean island groups. *Biological Journal of the Linnean Society*, **105**, 146–159.

Suarez, A.V., Bolger, D.T. & Case, T.J. (1998) Effects of fragmentation and invasion on native ant communities in coastal southern California. *Ecology*, **79**, 2041–2056.

Wang, Y., Bao, Y., Yu, M., Xu, G. & Ding, P. (2010) Nestedness for different reasons: the distributions of birds, lizards and small mammals on islands of an inundated lake. *Diversity and Distributions*, **16**, 862–873.

Wang, Y., Wu, Q., Wang, X., Liu, C., Wu, L., Chen, C., Ge, D., Song, X., Chen, C., Xu, A. & Ding, P. (2015) Small-island effect in snake communities on islands of an inundated lake: the need to include zeroes. *Basic and Applied Ecology*, **16**, 19–27.

Welter-Schultes, F.W. & Williams, M.R. (1999) History, island area and habitat availability determine land snail species richness of Aegean islands. *Journal of Biogeography*, **26**, 239–249.

Woinarski, J.C.Z., Horner, P., Fisher, A., Brennan, K., Lindner, D., Gambold, N., Chatto, R. & Morris, I. (1999 a) Distributional patterning of terrestrial herpetofauna on the Wessel and English Company Island groups, northeastern Arnhem Land, Northern Territory, Australia. *Australian Journal of Ecology*, **24**, 60–79.

Woinarski, J.C.Z., Palmer, C., Fisher, A., Southgate, R., Masters, P. & Brennan, K. (1999 b) Distributional patterning of mammals on the Wessel and English Company islands, Arnhem Land, Northern Territory, Australia. *Australian Journal of Zoology*, **47**, 87–111.

Woinarski, J.C.Z., Brennan, K., Cowie, I., Fisher, A., Latz, P.K. & Russell-Smith, J. (2000) Vegetation of the Wessel and English Company Islands, north-eastern Arnhem Land, northern territory. *Australian Journal of Botany*, **48**, 115–141.

Table S2 Results of piecewise and traditional species–area regression for 68 island datasets. For each model, the fitted parameters (*c*, *z* and *T*), the log-likelihood (log *L*), number of estimable parameters (K), Akaike's information criterion (AIC) or Akaike's information criterion corrected (AIC_c), Akaike differences ($\Delta AIC_{(c)}$), Bayesian information criterion (BIC), and BIC differences (ΔBIC) are presented. *T* is $\log_{10}$ of the area in km^2 of the breakpoint.

Group	Model[1]	Segment	Parameter estimate								Model selection[2]					
			c_1	c_2	c_3	z_1	z_2	z_3	T_1	T_2	*K*	log *L*	$AIC_{(c)}$	$\Delta AIC_{(c)}$	BIC	ΔBIC
Abbott &	(1)	1	2.187			0.418					3	-26.96	60.24	7.27	66.98	11.25
Black	(2)	2	1.825			0.335	0.575		-3.252		5	-25.22	54.06	1.09	59.16	3.43
(1980)	(3)	2	2.632			0.318	0.591		-3.252		5	-25.25	52.97	0.00	55.73	0.00
Western	(4)	2	2.501	2.651		0.475	0.596		-3.824		6	-22.52	58.22	5.25	71.18	15.45
Australia	(5)*	2	0.000			0.438			-5.151		4	-26.44	61.43	8.46	70.30	14.57
plants	(6)*	2	2.257			0.438			-5.150		4	-26.44	61.43	8.46	70.30	14.57
	(7)*	2	0.150	2.335		0.464			-4.699		5	-27.42	65.67	12.70	76.62	20.89
	(8)#	2	2.147			0.409			-0.794		4	-26.99	62.52	9.55	71.40	15.67
	(9)#	2	2.025			0.466			-0.794		4	-27.72	64.00	11.03	72.88	17.15
	(10)#	2	1.818	1.647		0.334			-2.668		5	-26.93	64.69	11.72	75.64	19.91
	(11)*	3	2.430	2.480		0.469	0.522		-5.179	-3.854	7	-25.39	66.37	13.40	81.27	25.54
	(12)*	3	0.182	3.942	2.651	0.801	0.596		-4.155	-3.824	8	-20.78	59.64	6.67	76.41	20.68
	(13)	3	2.035	-51.09	2.621	0.379	-13.51	0.583	-3.854	-3.796	9	-21.10	62.86	9.89	81.42	25.69
	(14)#	3	1.682	1.620	1.647	0.309	0.277		-4.398	-2.668	8	-22.05	62.18	9.21	78.95	23.22
	(15)	3	2.501	2.651		0.475	0.596		-3.853	-3.824	7	-22.52	60.64	7.67	75.53	19.80
Ackerman	(1)	1	-0.084			0.520					3	-28.65	63.83	3.26	68.97	0.00
et al.	(2)	2	6.129			-3.904	0.582		1.427		5	-27.01	64.02	3.45	73.48	4.51
(2007)	(3)	2	-0.451			0.075	0.635		2.088		5	-27.15	65.69	5.12	73.75	4.78
West	(4)	2	0.484	0.422		0.222	0.423		2.790		6	-25.53	65.05	4.48	74.41	5.44
Indies	(5)*	2	0.843			0.635			2.038		4	-27.17	63.24	2.67	69.90	0.93
orchids	(6)*	2	-0.451			0.635			2.038		4	-27.17	63.24	2.67	69.90	0.93
	(7)*	2	0.943	0.422		0.423			2.790		5	-26.61	64.62	4.05	72.68	3.71
	(8)#	2	-0.095			0.525			4.890		4	-28.64	66.18	5.61	72.84	3.87
	(9)#	2	2.474			0.525			4.892		4	-28.64	66.18	5.61	72.84	3.87
	(10)#	2	0.149	2.349		0.406			3.342		5	-27.55	66.49	5.92	74.56	5.59
	(11)*	3	-24.073	-0.098		9.248	0.544		2.702	2.875	7	-21.92	60.57	0.00	71.08	2.11
	(12)*	3	0.878	6.430	0.422	-2.007	0.423		2.478	2.790	8	-23.90	67.39	6.82	78.93	9.96
	(13)	3	0.656	6.430	0.422	0.116	-2.007	0.423	2.477	2.790	9	-23.71	70.03	9.46	82.44	13.47
	(14)#	3	0.484	11.815	1.957	0.222	-3.353		2.790	3.151	8	-20.51	60.61	0.04	72.15	3.18
	(15)	3	0.603	0.480		0.156	0.409		2.697	2.789	7	-24.24	65.22	4.65	75.73	6.76

Group	Model[1]	Segment	Parameter estimate								Model selection[2]					
			c_1	c_2	c_3	z_1	z_2	z_3	T_1	T_2	K	log L	AIC$_{(c)}$	ΔAIC$_{(c)}$	BIC	ΔBIC
Benchimol	(1)	1	1.750			0.058					3	75.66	-144.98	41.81	-138.37	32.00
& Peres	(2)	2	1.927			0.189	0.030		-1.023		5	79.05	-147.23	39.56	-136.51	33.86
(2015)	(3)	2	1.758			0.563	0.040		-1.664		5	76.74	-142.61	44.18	-131.89	38.48
Amazonia	(4)	2	-5.527	1.759		-3.441	0.039		-1.839		6	98.14	-183.04	3.75	-170.37	0.00
n forest	(5)*	2	1.633			0.059			-1.985		4	75.66	-142.75	44.04	-134.05	36.32
plants	(6)*	2	1.750			0.059			-1.992		4	75.66	-142.75	44.04	-134.05	36.32
	(7)*	2	1.390	1.759		0.039			-1.839		5	86.32	-161.77	25.02	-151.05	19.32
	(8)#	2	1.789			0.100			-0.005		4	78.35	-148.12	38.67	-139.42	30.95
	(9)#	2	1.787			0.099			-0.011		4	78.37	-148.16	38.63	-139.46	30.91
	(10)#	2	-5.527	1.762		-3.441			-1.939		5	89.97	-169.07	17.72	-158.35	12.02
	(11)*	3	5.166	1.757		1.978	0.043		-1.941	-1.569	7	81.05	-146.43	40.36	-131.88	38.49
	(12)*	3	1.390	2.279	1.757	0.316	0.043		-1.939	-1.664	8	86.74	-155.30	31.49	-138.94	31.43
	(13)	3	-5.527	1.213	1.765	-3.441	-0.286	0.027	-1.839	-1.233	9	103.78	-186.79	0.00	-168.70	1.67
	(14)#	3	-5.527	1.750	1.762	-3.441	0.059		-1.939	-1.839	8	100.88	-183.57	3.22	-167.21	3.16
	(15)	3	-5.527	1.759		-3.441	0.039		-1.842	-1.839	7	98.14	-180.60	6.19	-166.05	4.32
Brown &	(1)	1	2.223			0.056					3	52.36	-98.09	0.00	-93.50	0.00
Hutchings	(2)	2	2.280			0.091	0.036		-0.990		5	53.44	-95.22	2.87	-88.20	5.30
(1997)	(3)	2	2.225			0.642	0.037		-1.910		5	53.45	-95.22	2.87	-88.20	5.30
Amazonia	(4)	2	2.280	2.225		0.091	0.037		0.000		6	53.45	-92.49	5.60	-84.46	9.04
n forest	(5)*	2	2.097			0.063			-1.999		4	51.95	-94.83	3.26	-88.96	4.54
butterflies	(6)*	2	2.223			0.056			-2.000		4	52.36	-95.64	2.45	-89.77	3.73
	(7)*	2	2.097	2.225		0.037			-1.000		5	53.44	-95.22	2.87	-88.20	5.30
	(8)#	2	2.280			0.091			-0.451		4	52.89	-96.70	1.39	-90.83	2.67
	(9)#	2	2.239			0.091			-0.445		4	52.89	-96.70	1.39	-90.83	2.67
	(10)#	2	2.280	2.239		0.091			-1.000		5	52.89	-94.12	3.97	-87.09	6.41
	(11)*	3	2.097	2.225		0.018	0.037		-2.000	-1.000	7	53.45	-89.60	8.49	-80.73	12.77
	(12)*	3	2.097	2.189	2.225	0.019	0.037		-2.000	0.000	8	53.44	-86.53	11.56	-76.99	16.51
	(13)	3	2.097	2.189	2.225	0.018	0.019	0.037	-1.000	0.000	9	53.45	-83.27	14.82	-73.25	20.25
	(14)#	3	2.097	2.225	2.262	0.018	0.036		-2.000	0.000	8	53.45	-86.53	11.56	-76.99	16.51
	(15)	3	2.280	2.225		0.091	0.037		-0.999	0.000	7	53.45	-89.60	8.49	-80.73	12.77

Group	Model[1]	Segment	Parameter estimate								Model selection[2]					
			c_1	c_2	c_3	z_1	z_2	z_3	T_1	T_2	K	log L	$AIC_{(c)}$	$\Delta AIC_{(c)}$	BIC	ΔBIC
Buckley	(1)	1	1.794			0.317					3	24.41	-42.41	11.16	-36.49	3.31
(1985)	(2)	2	2.238			0.410	0.213		-4.022		5	26.39	-41.69	11.88	-32.22	7.58
Prince	(3)	2	1.481			0.400	0.224		-4.022		5	26.38	-41.66	11.91	-32.20	7.60
Charlotte	(4)	2	1.514	1.329		0.269	0.175		-4.310		6	30.31	-47.06	6.51	-35.95	3.85
plants	(5)*	2	0.000			0.330			-5.580		4	24.99	-41.26	12.31	-33.53	6.27
(Mangrov	(6)*	2	1.840			0.330			-5.582		4	24.99	-41.26	12.31	-33.53	6.27
e species)	(7)*	2	0.000	1.660		0.277			-4.854		5	30.18	-49.27	4.30	-39.80	0.00
	(8)#	2	1.839			0.327			-2.891		4	25.26	-41.81	11.76	-34.08	5.72
	(9)#	2	0.900			0.331			-2.891		4	25.25	-41.79	11.78	-34.06	5.74
	(10)#	2	1.711	0.733		0.307			-4.284		5	24.63	-38.17	15.40	-28.70	11.10
	(11)*	3	3.224	1.535		0.629	0.241		-5.110	-3.896	7	31.84	-47.57	6.00	-34.91	4.89
	(12)*	3	0.000	-0.369	1.329	-0.149	0.175		-4.854	-4.310	8	36.17	-53.57	0.00	-39.45	0.35
	(13)	3	1.601	-28.23	1.329	0.286	-6.522	0.175	-4.444	-4.310	9	35.33	-49.12	4.45	-33.65	6.15
	(14)#	3	1.514	1.211	0.891	0.269	0.144		-4.328	-2.957	8	30.97	-43.17	10.40	-29.05	10.75
	(15)	3	0.000	1.290		0.000	0.162		-5.152	-4.089	7	30.94	-45.76	7.81	-33.10	6.70
Buckley	(1)	1	2.724			0.540					3	2.86	0.70	21.82	6.61	20.00
(1985)	(2)	2	0.285			0.051	0.684		-4.664		5	14.97	-18.85	2.27	-9.38	4.01
Prince	(3)	2	3.236			0.051	0.684		-4.664		5	14.97	-18.85	2.27	-9.38	4.01
Charlotte	(4)	2	1.127	2.783		0.213	0.540		-4.022		6	14.06	-14.56	6.56	-3.45	9.94
plants	(5)*	2	0.000			0.671			-4.758		4	14.92	-21.12	0.00	-13.39	0.00
(Ridge	(6)*	2	3.192			0.671			-4.759		4	14.92	-21.12	0.00	-13.39	0.00
species)	(7)*	2	0.029	3.139		0.655			-4.509		5	14.80	-18.51	2.61	-9.04	4.35
	(8)#	2	2.728			0.541			-2.537		4	3.04	2.64	23.76	10.37	23.76
	(9)#	2	1.392			0.559			-2.537		4	2.91	2.90	24.02	10.63	24.02
	(10)#	2	2.113	1.130		0.412			-3.455		5	4.15	2.79	23.91	12.25	25.64
	(11)*	3	1.811	2.737		0.370	0.525		-4.800	-4.086	7	17.91	-19.70	1.42	-7.04	6.35
	(12)*	3	0.029	0.468	2.783	0.039	0.540		-4.509	-4.022	8	16.93	-15.08	6.04	-0.96	12.43
	(13)	3	0.299	0.468	2.783	0.055	0.039	0.540	-4.509	-4.022	9	17.09	-12.66	8.46	2.81	16.20
	(14)#	3	0.907	2.929	1.279	0.169	0.581		-4.086	-2.537	8	17.64	-16.51	4.61	-2.39	11.00
	(15)	3	0.906	2.737		0.169	0.525		-4.086	-4.023	7	16.83	-17.55	3.57	-4.88	8.51

Group	Model[1]	Segment	Parameter estimate								Model selection[2]					
			c_1	c_2	c_3	z_1	z_2	z_3	T_1	T_2	K	log L	$\text{AIC}_{(c)}$	$\Delta\text{AIC}_{(c)}$	BIC	ΔBIC
Buckley	(1)	1	1.279			0.177					3	21.31	-36.20	1.90	-30.29	0.00
(1985)	(2)	2	1.087			0.136	0.335		-3.495		5	22.72	-34.34	3.76	-24.88	5.41
Prince	(3)	2	1.812			0.131	0.345		-3.495		5	22.72	-34.34	3.76	-24.88	5.41
Charlotte	(4)	2	1.195	1.983		0.158	0.403		-3.564		6	23.26	-32.96	5.14	-21.85	8.44
plants	(5)*	2	0.239			0.179			-5.853		4	21.33	-33.95	4.15	-26.22	4.07
(Saltflat	(6)*	2	1.288			0.179			-5.851		4	21.33	-33.95	4.15	-26.22	4.07
species)	(7)*	2	0.159	1.237		0.165			-5.301		5	22.36	-33.63	4.47	-24.16	6.13
	(8)#	2	1.282			0.178			-2.537		4	21.37	-34.03	4.07	-26.30	3.99
	(9)#	2	0.841			0.182			-2.537		4	17.09	-25.47	12.63	-17.74	12.55
	(10)#	2	1.068	0.849		0.132			-3.130		5	24.13	-37.17	0.93	-27.70	2.59
	(11)*	3	1.759	1.827		0.276	0.352		-5.530	-4.022	7	24.76	-33.40	4.70	-20.74	9.55
	(12)*	3	0.216	0.723	1.543	0.048	0.251		-5.155	-3.428	8	25.68	-32.60	5.50	-18.48	11.81
	(13)	3	1.195	12.270	1.543	0.158	3.385	0.251	-3.564	-3.428	9	25.99	-30.45	7.65	-14.98	15.31
	(14)#	3	1.348	1.136	0.751	0.189	0.202		-3.570	-3.455	8	28.43	-38.10	0.00	-23.98	6.31
	(15)	3	1.576	1.983		0.235	0.403		-4.164	-3.563	7	24.98	-33.85	4.25	-21.19	9.10
Caujapé-	(1)	1	-0.314			0.270					3	-50.69	107.80	0.39	113.77	0.00
Castells et	(2)	2	1.308			-0.440	0.321		2.420		5	-49.59	110.25	2.84	119.81	6.04
al. (2010)	(3)	2	-0.444			-0.248	0.301		2.517		5	-49.73	110.53	3.12	120.10	6.33
Worldwid	(4)	2	-0.699	-0.594		0.688	0.334		2.285		6	-48.55	110.63	3.22	121.86	8.09
e plants	(5)*	2	0.311			0.302			2.510		4	-49.96	108.62	1.21	116.43	2.66
(CR	(6)*	2	-0.444			0.301			2.509		4	-49.96	108.62	1.21	116.43	2.66
endemic)	(7)*	2	0.621	-0.594		0.334			2.285		5	-48.81	108.70	1.29	118.26	4.49
	(8)#	2	-0.306			0.267			5.864		4	-50.67	110.04	2.63	117.85	4.08
	(9)#	2	1.316			0.287			5.864		4	-50.71	110.12	2.71	117.93	4.16
	(10)#	2	-0.413	0.301		0.302			5.865		5	-48.91	108.90	1.49	118.46	4.69
	(11)*	3	-0.450	397.46		0.303	-67.37		2.514	5.769	7	-46.20	108.47	1.06	121.29	7.52
	(12)*	3	0.621	-0.734	0.301	0.377	-		2.285	5.865	8	-46.47	111.67	4.26	125.97	12.20
	(13)	3	-0.159	-11.05	0.301	0.219	2.264	-	5.561	5.865	9	-45.45	112.35	4.94	128.04	14.27
	(14)#	3	-0.213	-15.68	0.301	0.237	3.041		5.471	5.865	8	-44.55	107.81	0.40	122.11	8.34
	(15)	3	-0.159	397.46		0.219	-67.37		5.561	5.864	7	-45.67	107.41	0.00	120.23	6.46

Group	Model[1]	Segment	Parameter estimate								Model selection[2]					
			c_1	c_2	c_3	z_1	z_2	z_3	T_1	T_2	K	log L	$AIC_{(c)}$	$\Delta AIC_{(c)}$	BIC	ΔBIC
Caujapé-Castells et al. (2010) Worldwide plants (EN endemic)	(1)	1	-0.236			0.250					3	-42.60	91.60	0.93	97.57	0.00
	(2)	2	-0.050			0.189	1.184		5.350		5	-41.44	93.95	3.28	103.52	5.95
	(3)	2	-5.367			0.189	1.183		5.349		5	-41.44	93.95	3.28	103.52	5.95
	(4)	2	-0.342	-3.311		0.295	0.821		4.379		6	-39.08	91.68	1.01	102.92	5.35
	(5)*	2	0.383			0.289			2.733		4	-42.12	92.95	2.28	100.76	3.19
	(6)*	2	-0.407			0.289			2.732		4	-42.12	92.95	2.28	100.76	3.19
	(7)*	2	0.496	-0.377		0.283			2.172		5	-41.87	94.81	4.14	104.37	6.80
	(8)#	2	-0.276			0.263			5.865		4	-42.61	93.93	3.26	101.74	4.17
	(9)#	2	1.165			0.282			5.286		4	-43.47	95.65	4.98	103.46	5.89
	(10)#	2	-0.040	1.604		0.186			5.561		5	-40.57	92.21	1.54	101.78	4.21
	(11)*	3	-6.620	-3.311		1.881	0.821		3.794	4.257	7	-37.57	91.21	0.54	104.03	6.46
	(12)*	3	0.467	-3.174	-3.311	1.060	0.821		3.966	4.379	8	-35.98	90.67	0.00	104.97	7.40
	(13)	3	-0.108	-8.012	-3.311	0.207	2.213	0.821	4.018	4.379	9	-37.40	96.26	5.59	111.94	14.37
	(14)#	3	-0.190	-13.28	1.604	0.236	2.577		5.142	5.561	8	-37.05	92.83	2.16	107.13	9.56
	(15)	3	-0.040	228.55		0.186	-38.62		5.561	5.852	7	-38.34	92.75	2.08	105.56	7.99
Caujapé-Castells et al. (2010) Worldwide plants (Total endemic)	(1)	1	-0.359			0.665					3	-48.96	104.34	5.11	110.30	0.60
	(2)	2	1.520			-0.135	0.708		2.442		5	-47.07	105.20	5.97	114.77	5.07
	(3)	2	-0.650			-0.220	0.732		2.442		5	-46.99	105.06	5.83	114.63	4.93
	(4)	2	2.376	-0.201		-0.599	0.635		2.679		6	-43.79	101.11	1.88	112.35	2.65
	(5)*	2	1.238			0.685			2.442		4	-47.32	103.34	4.11	111.15	1.45
	(6)*	2	-0.650			0.732			2.442		4	-47.15	103.00	3.77	110.81	1.11
	(7)*	2	1.113	0.071		0.578			2.899		5	-44.53	100.14	0.91	109.70	0.00
	(8)#	2	-0.302			0.647			5.894		4	-49.02	106.74	7.51	114.54	4.84
	(9)#	2	3.452			0.708			5.560		4	-50.05	108.80	9.57	116.60	6.90
	(10)#	2	-0.232	3.728		0.624			5.646		5	-48.69	108.44	9.21	118.01	8.31
	(11)*	3	-1.397	-2.956		0.990	1.135		2.514	4.257	7	-41.58	99.23	0.00	112.05	2.35
	(12)*	3	1.238	-1.790	-2.956	1.096	1.135		2.420	4.379	8	-40.41	99.55	0.32	113.85	4.15
	(13)	3	1.520	-1.790	-2.956	-0.135	1.096	1.135	2.420	4.379	9	-40.38	102.23	3.00	117.91	8.21
	(14)#	3	2.376	0.049	3.728	-0.599	0.564		2.665	5.646	8	-42.10	102.91	3.68	117.21	7.51
	(15)	3	2.348	0.269		-0.586	0.537		2.508	3.297	7	-43.68	103.44	4.21	116.25	6.55

Group	Model[1]	Segment	Parameter estimate								Model selection[2]					
			c_1	c_2	c_3	z_1	z_2	z_3	T_1	T_2	K	log L	$AIC_{(c)}$	$\Delta AIC_{(c)}$	BIC	ΔBIC
Cody	(1)	1	2.476			0.399					3	-52.31	110.73	31.08	120.70	11.68
(2006)	(2)	2	3.145			0.594	0.290		-2.967		5	-46.55	103.39	23.74	119.91	10.89
Barkley	(3)	2	2.243			0.595	0.290		-2.967		5	-46.55	103.39	23.74	119.91	10.89
Sound	(4)	2	-2.594	2.435		-0.740	0.382		-4.034		6	-49.62	111.66	32.01	131.42	22.40
Islands (E)	(5)[*]	2	0.751			0.406			-4.292		4	-52.21	112.61	32.96	125.86	16.84
plants	(6)[*]	2	2.477			0.400			-4.292		4	-52.18	112.54	32.89	125.80	16.78
	(7)[*]	2	0.588	2.409		0.370			-3.983		5	-48.34	106.97	27.32	123.48	14.46
	(8)[#]	2	2.647			0.453			-1.495		4	-48.59	105.36	25.71	118.62	9.60
	(9)[#]	2	1.969			0.453			-1.494		4	-48.59	105.37	25.72	118.62	9.60
	(10)[#]	2	2.660	1.948		0.458			-1.547		5	-48.61	107.51	27.86	124.03	15.01
	(11)[*]	3	-26.35	2.435		-6.259	0.382		-4.338	-4.201	7	-44.21	102.97	23.32	125.95	16.93
	(12)[*]	3	0.901	11.065	2.413	2.578	0.372		-4.292	-3.873	8	-43.42	103.54	23.89	129.73	20.71
	(13)	3	3.060	-104.3	2.177	0.571	-39.03	0.243	-2.719	-2.660	9	-30.38	79.65	0.00	109.02	0.00
	(14)[#]	3	3.060	2.472	2.013	0.571	0.392		-2.735	-1.335	8	-40.55	97.80	18.15	123.99	14.97
	(15)	3	-17.02	2.409		-4.080	0.370		-4.201	-3.938	7	-44.42	103.38	23.73	126.36	17.34
Corke	(1)	1	0.550			0.279					3	18.41	-30.28	0.00	-25.22	0.00
(1992)	(2)	2	0.520			0.256	0.320		0.001		5	18.63	-25.83	4.45	-17.90	7.32
Grenadine	(3)	2	0.534			0.265	0.309		0.001		5	18.61	-25.78	4.50	-17.86	7.36
Islands	(4)	2	0.072	0.561		0.029	0.255		-1.301		6	20.14	-26.23	4.05	-17.05	8.17
amphibia	(5)[*]	2	0.000			0.280			-1.957		4	18.42	-27.92	2.36	-21.36	3.86
and	(6)[*]	2	0.550			0.281			-1.959		4	18.43	-27.92	2.36	-21.37	3.85
reptiles	(7)[*]	2	0.020	0.561		0.255			-1.301		5	20.13	-28.83	1.45	-20.90	4.32
	(8)[#]	2	0.546			0.275			1.672		4	18.40	-27.87	2.41	-21.32	3.90
	(9)[#]	2	0.979			0.282			1.505		4	18.29	-27.65	2.63	-21.09	4.13
	(10)[#]	2	0.542	0.920		0.272			0.869		5	17.26	-23.08	7.20	-15.15	10.07
	(11)[*]	3	0.072	0.561		0.029	0.255		-2.000	-1.301	7	20.14	-23.48	6.80	-13.18	12.04
	(12)[*]	3	0.020	0.458	0.726	0.129	0.163		-1.301	0.301	8	23.23	-26.76	3.52	-15.48	9.74
	(13)	3	0.072	0.458	0.726	0.029	0.129	0.163	-1.301	0.301	9	23.24	-23.75	6.53	-11.64	13.58
	(14)[#]	3	0.500	0.491	0.907	0.250	0.220		-1.699	0.301	8	22.67	-25.66	4.62	-14.38	10.84
	(15)	3	0.072	0.561		0.029	0.255		-1.397	-1.302	7	20.14	-23.48	6.80	-13.18	12.04

Group	Model[1]	Segment	Parameter estimate								Model selection[2]					
			c_1	c_2	c_3	z_1	z_2	z_3	T_1	T_2	K	log L	$AIC_{(c)}$	$\Delta AIC_{(c)}$	BIC	ΔBIC
Corke	(1)	1	0.580			0.224					3	1.91	2.68	11.89	8.04	7.41
(1992)	(2)	2	0.343			0.070	0.287		-0.552		5	3.92	3.46	12.67	11.91	11.28
Winward	(3)	2	0.542			0.155	0.256		-0.552		5	3.28	4.73	13.94	13.19	12.56
Islands	(4)	2	0.895	0.366		0.385	0.324		-1.000		6	11.54	-9.21	0.00	0.63	0.00
amphibia	(5)*	2	0.243			0.306			-0.553		4	3.37	2.10	11.31	9.06	8.43
and	(6)*	2	0.580			0.224			-2.000		4	1.91	5.03	14.24	11.99	11.36
reptiles	(7)*	2	0.266	0.366		0.324			-1.000		5	4.74	1.82	11.03	10.28	9.65
	(8)#	2	0.582			0.225			2.880		4	1.92	5.01	14.22	11.97	11.34
	(9)#	2	1.230			0.225			2.883		4	1.92	5.02	14.23	11.97	11.34
	(10)#	2	0.374	1.217		0.089			-0.097		5	3.91	3.48	12.69	11.93	11.30
	(11)*	3	0.895	0.366		0.385	0.324		-2.000	-1.046	7	11.54	-6.53	2.68	4.58	3.95
	(12)*	3	0.183	1.224	0.366	0.649	0.324		-1.699	-1.000	8	10.82	-2.30	6.91	9.96	9.33
	(13)	3	0.712	0.753	0.366	0.284	0.000	0.324	-1.097	-1.000	9	13.98	-5.68	3.53	7.59	6.96
	(14)#	3	1.063	0.200	1.217	0.467	-0.086		-1.699	-0.097	8	12.05	-4.76	4.45	7.50	6.87
	(15)	3	0.895	0.366		0.385	0.324		-1.045	-1.001	7	11.54	-6.53	2.68	4.58	3.95
Davies &	(1)	1	1.045			0.205					3	12.26	-18.15	7.83	-11.87	0.98
Smith	(2)	2	1.108			0.160	0.406		3.137		5	16.55	-22.13	3.85	-12.00	0.85
(1998)	(3)	2	0.293			0.154	0.416		3.137		5	16.53	-22.09	3.89	-11.96	0.89
West	(4)	2	1.108	0.803		0.159	0.301		3.342		6	16.42	-19.45	6.53	-7.51	5.34
Indies	(5)*	2	1.177			0.261			1.097		4	14.87	-21.10	4.88	-12.85	0.00
butterfly	(6)*	2	0.891			0.261			1.097		4	14.87	-21.10	4.88	-12.85	0.00
	(7)*	2	1.268	0.865		0.273			2.212		5	13.70	-16.43	9.55	-6.30	6.55
	(8)#	2	1.055			0.198			5.035		4	12.20	-15.77	10.21	-7.53	5.32
	(9)#	2	2.260			0.257			5.035		4	8.77	-8.90	17.08	-0.66	12.19
	(10)#	2	1.122	2.081		0.148			3.226		5	16.96	-22.96	3.02	-12.83	0.02
	(11)*	3	1.135	1.191		0.142	0.215		-0.523	3.226	7	18.39	-20.92	5.06	-7.25	5.60
	(12)*	3	1.268	1.444	0.803	0.051	0.301		2.212	3.342	8	15.78	-13.12	12.86	2.20	15.05
	(13)	3	1.148	1.444	0.803	0.101	0.051	0.301	2.212	3.342	9	19.13	-17.17	8.81	-0.29	12.56
	(14)#	3	1.157	1.822	2.081	0.086	-0.098		2.207	3.226	8	22.21	-25.98	0.00	-10.66	2.19
	(15)	3	1.122	1.191		0.148	0.215		3.226	3.342	7	18.43	-21.00	4.98	-7.33	5.52

Group	Model[1]	Segment	Parameter estimate								Model selection[2]					
			c_1	c_2	c_3	z_1	z_2	z_3	T_1	T_2	K	log L	$AIC_{(c)}$	$\Delta AIC_{(c)}$	BIC	ΔBIC
Dennis &	(1)	1	1.070			0.039					3	8.67	-10.98	4.06	-4.46	1.01
Shreeve	(2)	2	1.112			0.211	0.001		0.153		5	11.31	-11.72	3.32	-1.16	4.31
(1997)	(3)	2	1.158			0.211	-0.008		0.211		5	11.35	-11.80	3.24	-1.24	4.23
British	(4)	2	1.116	1.028		0.219	0.048		0.812		6	13.59	-13.91	1.13	-1.44	4.03
Islands	(5)[*]	2	0.954			0.063			-1.221		4	7.92	-7.26	7.78	1.32	6.79
butterfly	(6)[*]	2	1.070			0.039			-1.222		4	8.67	-8.74	6.30	-0.17	5.30
	(7)[*]	2	0.950	1.160		-0.009			-0.046		5	11.31	-11.73	3.31	-1.17	4.30
	(8)[#]	2	1.112			0.211			0.160		4	11.32	-14.04	1.00	-5.47	0.00
	(9)[#]	2	1.145			0.211			0.157		4	11.32	-14.04	1.00	-5.47	0.00
	(10)[#]	2	1.116	1.121		0.219			0.806		5	12.97	-15.04	0.00	-4.48	0.99
	(11)[*]	3	1.112	1.028		0.232	0.048		-1.102	0.806	7	13.64	-11.55	3.49	2.76	8.23
	(12)[*]	3	0.950	1.196	2.623	-0.054	-0.491		-0.046	2.581	8	14.42	-10.58	4.46	5.49	10.96
	(13)	3	1.116	5.315	1.116	0.219	-5.193	0.012	0.812	0.900	9	16.62	-12.39	2.65	5.37	10.84
	(14)[#]	3	1.116	8.159	1.131	0.219	-8.641		0.806	0.890	8	16.59	-14.93	0.11	1.15	6.62
	(15)	3	1.116	1.018		0.219	0.052		0.807	0.814	7	13.67	-11.62	3.42	2.69	8.16
Diamond	(1)	1	0.050			0.133					3	-9.93	26.37	30.58	31.59	24.75
& Mayr	(2)	2	0.000			0.000	0.468		1.786		5	4.40	2.56	6.77	10.75	3.91
(1976)	(3)	2	-0.837			0.007	0.468		1.829		5	4.37	2.62	6.83	10.81	3.97
Solomon	(4)	2	0.045	-4.879		0.071	1.590		2.848		6	2.45	9.06	13.27	18.58	11.74
Islands	(5)[*]	2	0.000			0.468			1.786		4	4.40	0.08	4.29	6.84	0.00
birds	(6)[*]	2	-0.837			0.468			1.787		4	4.40	0.08	4.29	6.84	0.00
(Mountain	(7)[*]	2	0.033	-0.920		0.498			2.581		5	4.55	2.27	6.48	10.47	3.63
species)	(8)[#]	2	0.050			0.120			3.934		4	-10.13	29.14	33.35	35.90	29.06
	(9)[#]	2	0.387			0.151			1.485		4	-18.63	46.14	50.35	52.90	46.06
	(10)[#]	2	0.036	0.884		0.051			2.835		5	4.96	1.45	5.66	9.64	2.80
	(11)[*]	3	-0.509	-1.703		0.327	0.764		1.555	3.634	7	8.12	0.42	4.63	11.14	4.30
	(12)[*]	3	0.033	2.780	-4.879	-0.826	1.590		2.581	2.848	8	8.89	1.73	5.94	13.52	6.68
	(13)	3	0.045	-5.861	-3.576	0.071	1.861	1.242	2.848	3.490	9	2.68	17.15	21.36	29.85	23.01
	(14)[#]	3	0.036	1.204	0.838	0.051	0.000		2.835	2.848	8	11.86	-4.21	0.00	7.57	0.73
	(15)	3	0.000	-4.178		0.000	1.401		1.507	3.383	7	7.13	2.40	6.61	13.12	6.28

Group	Model[1]	Segment	Parameter estimate								Model selection[2]					
			c_1	c_2	c_3	z_1	z_2	z_3	T_1	T_2	K	log L	AIC$_{(c)}$	ΔAIC$_{(c)}$	BIC	ΔBIC
Diamond	(1)	1	1.291			0.172					3	22.24	-37.95	2.55	-32.74	0.00
& Mayr	(2)	2	1.294			0.186	0.097		2.405		5	23.06	-34.75	5.75	-26.55	6.19
(1976)	(3)	2	1.509			0.186	0.097		2.401		5	23.06	-34.75	5.75	-26.55	6.19
Solomon	(4)	2	1.205	1.450		0.106	0.116		1.045		6	26.57	-39.18	1.32	-29.66	3.08
Islands	(5)[*]	2	0.903			0.176			-2.154		4	22.16	-35.43	5.07	-28.67	4.07
birds (Sea-	(6)[*]	2	1.292			0.171			-2.155		4	22.24	-35.58	4.92	-28.82	3.92
level	(7)[*]	2	1.127	1.450		0.116			1.045		5	22.08	-32.79	7.71	-24.59	8.15
species)	(8)[#]	2	1.293			0.182			3.107		4	22.79	-36.68	3.82	-29.92	2.82
	(9)[#]	2	1.857			0.182			3.103		4	22.79	-36.68	3.82	-29.92	2.82
	(10)[#]	2	1.293	1.857		0.182			2.848		5	22.79	-34.21	6.29	-26.01	6.73
	(11)[*]	3	-2.100	1.346		5.299	0.152		0.605	0.980	7	23.56	-30.45	10.05	-19.74	13.00
	(12)[*]	3	0.841	1.202	1.450	0.075	0.116		-1.794	1.045	8	28.09	-36.66	3.84	-24.88	7.86
	(13)	3	1.217	1.314	1.450	0.116	-0.031	0.116	0.114	1.045	9	26.65	-30.79	9.71	-18.08	14.66
	(14)[#]	3	1.448	1.019	1.815	0.280	0.355		-0.273	2.365	8	30.00	-40.50	0.00	-28.71	4.03
	(15)	3	1.174	1.450		0.081	0.116		1.012	1.054	7	27.75	-38.84	1.66	-28.12	4.62
Dickman	(1)	1	0.902			0.040					3	-0.44	7.40	7.27	12.62	6.90
(1987)	(2)	2	6.474			2.184	-0.043		-2.571		5	4.56	2.24	1.09	10.44	1.74
Oxford	(3)	2	0.593			2.179	-0.133		-2.538		5	5.34	0.68	0.00	8.88	0.18
City	(4)	2	4.345	0.563		1.390	-0.151		-2.444		6	4.96	4.03	5.25	13.55	3.53
mammals	(5)[*]	2	0.821			0.001			-0.700		4	-0.58	10.05	8.46	16.81	9.55
	(6)[*]	2	0.615			-0.106			-2.027		4	-0.22	9.33	8.46	16.09	8.83
	(7)[*]	2	0.605	0.618		-0.119			-2.602		5	4.20	2.96	12.70	11.16	2.46
	(8)[#]	2	6.474			2.184			-2.571		4	4.19	0.50	9.55	7.26	0.00
	(9)[#]	2	0.859			2.184			-2.571		4	4.19	0.50	11.03	7.26	0.00
	(10)[#]	2	2.278	0.792		0.608			-2.046		5	4.92	1.52	11.72	9.72	1.02
	(11)[*]	3	5.040	0.598		1.641	-0.130		-2.796	-2.469	7	5.31	6.05	13.40	16.77	5.55
	(12)[*]	3	0.624	0.645	0.602	-0.108	-		-2.523	-1.131	8	4.27	10.96	6.67	22.75	10.46
	(13)	3	2.278	126.97	0.553	0.608	65.06	-0.16	-1.947	-1.928	9	8.92	4.66	9.89	17.37	4.16
	(14)[#]	3	2.278	0.555	0.792	0.608	-0.151		-2.046	-1.947	8	8.42	2.66	9.21	14.45	2.16
	(15)	3	6.474	0.459		2.184	-0.220		-2.573	-2.098	7	5.60	5.46	7.67	16.18	4.96

Group	Model[1]	Segment	Parameter estimate								Model selection[2]					
			c_1	c_2	c_3	z_1	z_2	z_3	T_1	T_2	K	log L	$AIC_{(c)}$	$\Delta AIC_{(c)}$	BIC	ΔBIC
Essl &	(1)	1	1.483			0.183					3	58.90	-111.38	3.98	-105.53	2.18
Dirnböck	(2)	2	1.637			0.239	0.019		-1.955		5	62.07	-113.03	2.33	-103.67	4.04
(2012)	(3)	2	1.206			0.239	0.019		-1.955		5	62.07	-113.03	2.33	-103.67	4.04
Austria	(4)	2	1.808	1.416		0.295	0.150		-2.959		6	60.70	-107.82	7.54	-96.84	10.87
semi-	(5)*	2	0.845			0.183			-3.490		4	58.90	-109.08	6.28	-101.43	6.28
natural	(6)*	2	1.483			0.183			-3.491		4	58.90	-109.08	6.28	-101.43	6.28
grassland	(7)*	2	0.870	1.416		0.150			-2.959		5	59.24	-107.36	8.00	-98.00	9.71
Orthoptera	(8)#	2	1.645			0.242			-1.943		4	62.04	-115.36	0.00	-107.71	0.00
species	(9)#	2	1.174			0.241			-1.943		4	62.04	-115.36	0.00	-107.71	0.00
	(10)#	2	1.590	1.176		0.223			-2.194		5	62.47	-113.82	1.54	-104.46	3.25
	(11)*	3	7.081	1.419		2.052	0.151		-3.045	-2.886	7	63.28	-110.41	4.95	-97.90	9.81
	(12)*	3	0.870	-0.977	1.361	-0.688	0.119		-2.959	-2.678	8	64.21	-109.59	5.77	-95.66	12.05
	(13)	3	1.808	-0.977	1.361	0.295	-0.688	0.119	-2.959	-2.678	9	65.95	-110.29	5.07	-95.04	12.67
	(14)#	3	0.913	1.270	1.176	0.023	0.094		-3.000	-2.194	8	66.22	-113.61	1.75	-99.68	8.03
	(15)	3	0.913	1.392		0.023	0.138		-3.000	-2.959	7	63.45	-110.76	4.60	-98.25	9.46
Flannery	(1)	1	-0.042			0.320					3	-94.83	195.77	17.94	205.67	11.29
(1995)	(2)	2	0.297			-0.025	0.413		1.359		5	-86.49	183.28	5.45	199.67	5.29
South-	(3)	2	-0.300			-0.001	0.414		1.411		5	-86.52	183.34	5.51	199.73	5.35
West	(4)	2	0.125	0.470		0.185	0.205		2.732		6	-84.81	182.04	4.21	201.65	7.27
Pacific	(5)*	2	0.277			0.413			1.393		4	-86.51	181.23	3.40	194.38	0.00
and	(6)*	2	-0.299			0.413			1.395		4	-86.51	181.23	3.40	194.38	0.00
Moluccan	(7)*	2	0.265	-0.246		0.396			1.538		5	-86.00	182.30	4.47	198.69	4.31
Islands	(8)#	2	-0.050			0.325			4.561		4	-94.62	197.45	19.62	210.60	16.22
mammals	(9)#	2	1.431			0.325			4.559		4	-94.62	197.45	19.62	210.60	16.22
	(10)#	2	0.132	1.158		0.179			2.726		5	-87.07	184.43	6.60	200.82	6.44
	(11)*	3	-0.147	0.466		0.318	0.206		1.378	2.726	7	-81.64	177.83	0.00	200.64	6.26
	(12)*	3	0.265	-0.002	0.470	0.258	0.205		1.538	2.732	8	-81.11	178.95	1.12	204.92	10.54
	(13)	3	0.310	-0.002	0.470	-0.048	0.258	0.205	1.538	2.732	9	-80.98	180.86	3.03	209.99	15.61
	(14)#	3	0.143	36.43	1.158	0.168	-13.26		2.643	2.726	8	-82.62	181.96	4.13	207.93	13.55
	(15)	3	0.132	0.469		0.179	0.205		2.726	2.733	7	-84.28	183.12	5.29	205.92	11.54

Group	Model[1]	Segment	Parameter estimate								Model selection[2]					
			c_1	c_2	c_3	z_1	z_2	z_3	T_1	T_2	K	log L	AIC$_{(c)}$	ΔAIC$_{(c)}$	BIC	ΔBIC
Hannus &	(1)	1	2.405			0.279					3	76.94	-147.57	35.62	-140.65	30.85
von	(2)	2	7.856			2.082	0.249		-2.998		5	96.77	-182.75	0.44	-171.50	0.00
Numers	(3)	2	2.360			2.082	0.249		-2.998		5	96.77	-182.75	0.44	-171.50	0.00
(2008)	(4)	2	6.146	2.354		1.559	0.245		-3.000		6	98.16	-183.19	0.00	-169.87	1.63
Brunskär	(5)*	2	1.707			0.336			-2.251		4	55.01	-101.50	81.69	-92.39	79.11
Islands,	(6)*	2	2.405			0.279			-3.301		4	76.94	-145.35	37.84	-136.25	35.25
S.W.	(7)*	2	1.199	2.354		0.245			-3.046		5	91.34	-171.90	11.29	-160.65	10.85
Finland	(8)#	2	2.407			0.280			-0.214		4	76.97	-145.43	37.76	-136.32	35.18
plants	(9)#	2	2.347			0.280			-0.214		4	76.98	-145.43	37.76	-136.32	35.18
	(10)#	2	2.407	2.347		0.280			-0.185		5	76.97	-143.16	40.03	-131.91	39.59
	(11)*	3	22.008	2.360		6.767	0.249		-3.101	-2.854	7	98.89	-182.27	0.92	-166.94	4.56
	(12)*	3	1.199	2.459	2.377	0.279	0.263		-3.046	-2.244	8	92.48	-166.98	16.21	-149.70	21.80
	(13)	3	6.146	2.459	2.377	1.559	0.279	0.263	-3.000	-2.244	9	99.50	-178.50	4.69	-159.33	12.17
	(14)#	3	6.146	2.344	2.347	1.559	0.240		-3.046	-0.454	8	98.51	-179.04	4.15	-161.76	9.74
	(15)	3	6.146	2.354		1.559	0.245		-3.045	-3.000	7	98.16	-180.80	2.39	-165.47	6.03
Hannus &	(1)	1	2.611			0.376					3	-8.07	22.46	12.03	29.21	0.22
von	(2)	2	9.530			2.025	0.356		-4.173		5	-6.63	24.09	13.66	35.04	6.05
Numers	(3)	2	2.555			1.544	0.352		-4.097		5	-6.52	23.87	13.44	34.82	5.83
(2008)	(4)	2	5.530	2.664		1.081	0.402		-3.721		6	-6.04	25.27	14.84	38.22	9.23
Getskär	(5)*	2	1.064			0.388			-4.045		4	-9.40	27.34	16.91	36.22	7.23
Islands,	(6)*	2	2.590			0.367			-4.398		4	-8.12	24.78	14.35	33.66	4.67
S.W.	(7)*	2	1.110	2.544		0.339			-3.260		5	-6.30	23.44	13.01	34.39	5.40
Finland	(8)#	2	2.632			0.383			-1.117		4	-8.05	24.64	14.21	33.52	4.53
plants	(9)#	2	2.195			0.378			-1.117		4	-8.05	24.65	14.22	33.53	4.54
	(10)#	2	2.625	2.212		0.380			-1.202		5	-8.04	26.92	16.49	37.87	8.88
	(11)*	3	6.602	2.661		1.336	0.401		-4.398	-3.770	7	-5.04	25.69	15.26	40.58	11.59
	(12)*	3	1.141	85.317	2.507	25.852	0.320		-3.292	-3.244	8	2.24	13.61	3.18	30.38	1.39
	(13)	3	2.594	85.317	2.507	0.369	25.852	0.320	-3.292	-3.244	9	5.11	10.43	0.00	28.99	0.00
	(14)#	3	2.373	2.553	2.208	0.318	0.343		-3.022	-0.976	8	-3.79	25.66	15.23	42.43	13.44
	(15)	3	7.878	2.664		1.645	0.402		-3.794	-3.715	7	-4.85	25.29	14.86	40.19	11.20

Group	Model[1]	Segment	Parameter estimate								Model selection[2]					
			c_1	c_2	c_3	z_1	z_2	z_3	T_1	T_2	K	log L	AIC$_{(c)}$	ΔAIC$_{(c)}$	BIC	ΔBIC
Hogg et	(1)	1	2.277			0.287					3	18.02	-29.70	4.39	-23.00	0.00
al. (1989)	(2)	2	2.518			0.424	0.239		-1.364		5	19.86	-28.87	5.22	-17.99	5.01
Great	(3)	2	2.266			0.424	0.239		-1.365		5	19.86	-28.87	5.22	-17.99	5.01
Lakes,	(4)	2	1.907	2.267		0.144	0.225		-1.638		6	22.46	-31.71	2.38	-18.85	4.15
Canada	(5)*	2	1.619			0.298			-2.221		4	17.93	-27.30	6.79	-18.48	4.52
plants	(6)*	2	2.276			0.286			-2.699		4	18.01	-27.47	6.62	-18.65	4.35
	(7)*	2	1.609	2.267		0.225			-1.638		5	21.99	-33.13	0.96	-22.26	0.74
	(8)#	2	2.284			0.293			1.200		4	18.27	-27.98	6.11	-19.17	3.83
	(9)#	2	2.636			0.293			1.201		4	18.27	-27.98	6.11	-19.16	3.84
	(10)#	2	2.285	2.627		0.294			1.171		5	18.27	-25.69	8.40	-14.82	8.18
	(11)*	3	5.894	2.267		2.443	0.234		-1.779	-1.538	7	24.86	-34.09	0.00	-19.31	3.69
	(12)*	3	1.548	2.620	2.267	0.508	0.225		-1.959	-1.638	8	23.93	-29.73	4.36	-13.10	9.90
	(13)	3	1.062	2.620	2.267	-0.218	0.508	0.225	-1.959	-1.638	9	24.36	-28.02	6.07	-9.62	13.38
	(14)#	3	1.633	2.252	2.610	0.022	0.205		-1.770	0.724	8	24.99	-31.87	2.22	-15.24	7.76
	(15)	3	1.633	2.267		0.022	0.221		-1.769	-1.639	7	24.61	-33.59	0.50	-18.81	4.19
Hu et al.	(1)	1	1.968			0.106					3	117.05	-227.93	0.70	-219.02	0.00
(2012)	(2)	2	2.157			0.177	0.093		-2.486		5	117.98	-225.56	3.07	-210.85	8.17
Thousand	(3)	2	1.947			0.177	0.093		-2.484		5	117.98	-225.56	3.07	-210.85	8.17
Island	(4)	2	2.062	1.990		0.143	0.148		-1.650		6	120.22	-227.86	0.77	-210.29	8.73
Lake,	(5)*	2	1.568			0.110			-3.698		4	117.01	-225.74	2.89	-213.92	5.10
China	(6)*	2	1.967			0.106			-3.699		4	117.05	-225.82	2.81	-214.00	5.02
plants	(7)*	2	1.547	1.952		0.096			-2.921		5	119.52	-228.63	0.00	-213.92	5.10
	(8)#	2	1.967			0.106			0.108		4	117.05	-225.83	2.80	-214.01	5.01
	(9)#	2	1.984			0.108			0.108		4	117.03	-225.79	2.84	-213.97	5.05
	(10)#	2	1.944	1.955		0.096			-0.907		5	117.70	-224.98	3.65	-210.27	8.75
	(11)*	3	2.073	1.990		0.148	0.148		-3.299	-1.652	7	120.37	-225.96	2.67	-205.57	13.45
	(12)*	3	1.547	2.018	1.990	0.122	0.148		-2.921	-1.650	8	122.03	-227.06	1.57	-203.88	15.14
	(13)	3	2.062	3.338	1.999	0.143	1.002	0.174	-1.650	-1.428	9	123.62	-227.97	0.66	-202.02	17.00
	(14)#	3	2.061	1.865	1.955	0.143	0.057		-1.695	-0.907	8	121.10	-225.19	3.44	-202.01	17.01
	(15)	3	2.052	1.990		0.139	0.148		-1.652	-1.650	7	120.10	-225.42	3.21	-205.03	13.99

Group	Model[1]	Segment	Parameter estimate								Model selection[2]					
			c_1	c_2	c_3	z_1	z_2	z_3	T_1	T_2	K	log L	AIC$_{(c)}$	ΔAIC$_{(c)}$	BIC	ΔBIC
Kohn & Walsh (1994) North Great Britain plants	(1)	1	2.093			0.511					3	-8.36	23.29	10.75	28.28	6.74
	(2)	2	-24.82			-7.347	0.548		-3.417		5	-5.11	21.69	9.15	29.48	7.94
	(3)	2	1.854			0.679	0.292		-1.764		5	-6.11	23.69	11.15	31.47	9.93
	(4)	2	-1.788	1.998		-0.699	0.429		-2.839		6	0.78	12.54	0.00	21.54	0.00
	(5)[*]	2	0.433			0.570			-3.065		4	-7.17	23.29	10.75	29.74	8.20
	(6)[*]	2	2.145			0.544			-3.066		4	-6.76	22.47	9.93	28.92	7.38
	(7)[*]	2	0.358	1.998		0.429			-2.839		5	-1.27	14.00	1.46	21.79	0.25
	(8)[#]	2	2.266			0.580			-0.883		4	-6.84	22.64	10.10	29.09	7.55
	(9)[#]	2	1.754			0.580			-0.882		4	-6.84	22.64	10.10	29.09	7.55
	(10)[#]	2	2.266	1.754		0.580			-0.937		5	-6.84	25.15	12.61	32.94	11.40
	(11)[*]	3	-0.320	1.998		-0.204	0.429		-3.509	-2.863	7	-0.51	17.88	5.34	27.96	6.42
	(12)[*]	3	0.716	1.138	1.998	0.289	0.429		-3.444	-2.839	8	1.48	16.83	4.29	27.84	6.30
	(13)	3	-3.357	-14.74	1.998	-1.167	-5.190	0.429	-3.013	-2.839	9	2.31	18.24	5.70	30.03	8.49
	(14)[#]	3	-1.788	2.152	1.754	-0.699	0.500		-2.863	-0.937	8	1.69	16.41	3.87	27.42	5.88
	(15)	3	-4.999	1.969		-1.650	0.405		-3.070	-2.687	7	1.70	13.46	0.92	23.54	2.00
Lavin et al. (2001) Various Islands plants (Endemic taxa)	(1)	1	-0.456			0.295					3	-54.12	114.65	6.06	120.72	1.02
	(2)	2	-0.190			0.201	2.378		5.364		5	-49.70	110.44	1.85	120.20	0.50
	(3)	2	-10.33			0.242	2.112		5.364		5	-49.94	110.92	2.33	120.68	0.98
	(4)	2	-0.493	-9.389		0.315	1.927		4.926		6	-47.56	108.59	0.00	120.07	0.37
	(5)[*]	2	0.194			0.331			2.457		4	-53.79	116.26	7.67	124.22	4.52
	(6)[*]	2	-0.636			0.335			2.457		4	-53.79	116.26	7.67	124.22	4.52
	(7)[*]	2	0.165	-0.427		0.291			2.733		5	-53.56	118.15	9.56	127.91	8.21
	(8)[#]	2	-0.396			0.274			5.918		4	-54.22	117.12	8.53	125.07	5.37
	(9)[#]	2	1.020			0.322			4.752		4	-55.89	120.45	11.86	128.41	8.71
	(10)[#]	2	-0.227	1.977		0.214			5.577		5	-49.45	109.94	1.35	119.70	0.00
	(11)[*]	3	-0.790	-9.389		0.392	1.927		2.449	4.882	7	-47.04	110.08	1.49	123.19	3.49
	(12)[*]	3	0.315	2.408	-11.54	-0.347	2.320		3.965	5.432	8	-49.36	117.33	8.74	131.99	12.29
	(13)	3	-0.340	-44.47	-11.54	0.256	8.377	2.320	5.045	5.432	9	-46.78	114.90	6.31	131.00	11.30
	(14)[#]	3	-0.493	2.824	1.407	0.315	-0.476		4.882	5.363	8	-45.84	110.29	1.70	124.95	5.25
	(15)	3	-0.512	-16.89		0.322	3.251		4.760	5.272	7	-46.60	109.19	0.60	122.30	2.60

Group	Model[1]	Segment	Parameter estimate								Model selection[2]					
			c_1	c_2	c_3	z_1	z_2	z_3	T_1	T_2	K	log L	AIC$_{(c)}$	ΔAIC$_{(c)}$	BIC	ΔBIC
Lawesson	(1)	1	2.075			0.156					3	39.06	-71.70	0.00	-65.74	0.00
et al.	(2)	2	2.077			0.158	-1.272		0.621		5	39.10	-67.13	4.57	-57.57	8.17
(1998)	(3)	2	2.074			0.325	0.147		-1.570		5	39.24	-67.41	4.29	-57.84	7.90
Danish	(4)	2	2.511	2.077		0.409	0.187		-1.208		6	41.23	-68.93	2.77	-57.69	8.05
Beech	(5)*	2	1.756			0.164			-1.958		4	39.01	-69.31	2.39	-61.51	4.23
Forests	(6)*	2	2.075			0.155			-1.959		4	39.06	-69.41	2.29	-61.61	4.13
plants (All	(7)*	2	1.805	2.075		0.156			-1.569		5	38.62	-66.16	5.54	-56.59	9.15
species)	(8)#	2	2.076			0.157			0.531		4	39.09	-69.48	2.22	-61.67	4.07
	(9)#	2	2.162			0.159			0.531		4	39.09	-69.47	2.23	-61.67	4.07
	(10)#	2	2.055	2.118		0.139			-0.097		5	39.72	-68.36	3.34	-58.79	6.95
	(11)*	3	2.918	2.077		0.684	0.180		-1.869	-1.244	7	41.02	-65.97	5.73	-53.15	12.59
	(12)*	3	1.846	-0.299	2.076	-1.870	0.155		-1.337	-1.041	8	41.29	-63.87	7.83	-49.57	16.17
	(13)	3	2.016	0.506	2.077	0.135	-1.226	0.187	-1.444	-1.208	9	43.15	-64.84	6.86	-49.16	16.58
	(14)#	3	2.072	2.117	2.095	0.154	0.000		-0.231	-0.210	8	42.17	-65.62	6.08	-51.32	14.42
	(15)	3	2.622	2.077		0.473	0.187		-1.234	-1.186	7	41.51	-66.94	4.76	-54.12	11.62
Lawlor	(1)	1	0.315			0.172					3	-39.43	84.85	19.86	96.86	10.98
(1986)	(2)	2	0.345			0.150	0.910		4.741		5	-27.94	65.87	0.88	85.88	0.00
Worldwid	(3)	2	-3.253			0.150	0.909		4.740		5	-27.94	65.87	0.88	85.88	0.00
e	(4)	2	0.339	-1.073		0.157	0.492		3.422		6	-26.50	64.99	0.00	89.00	3.12
terrestrial	(5)*	2	0.351			0.181			0.327		4	-38.36	84.73	19.74	100.73	14.85
mammals	(6)*	2	0.292			0.181			0.326		4	-38.36	84.73	19.74	100.73	14.85
	(7)*	2	0.355	0.292		0.181			0.279		5	-38.35	86.70	21.71	106.71	20.83
	(8)#	2	0.322			0.167			5.871		4	-39.55	87.09	22.10	103.10	17.22
	(9)#	2	1.160			0.231			3.934		4	-66.87	141.75	76.76	157.75	71.87
	(10)#	2	0.339	1.771		0.155			5.047		5	-29.07	68.15	3.16	88.15	2.27
	(11)*	3	0.336	-0.885		0.157	0.453		-0.221	3.391	7	-28.39	70.78	5.79	98.79	12.91
	(12)*	3	0.365	0.347	-1.073	0.154	0.492		0.716	3.422	8	-25.79	67.59	2.60	99.60	13.72
	(13)	3	0.343	-35.49	-0.587	0.153	11.35	0.390	3.179	3.240	9	-29.06	76.12	11.13	112.13	26.25
	(14)#	3	0.339	0.230	1.771	0.157	0.185		2.580	5.047	8	-24.61	65.23	0.24	97.24	11.36
	(15)	3	0.339	-1.148		0.157	0.508		3.391	3.475	7	-26.27	66.55	1.56	94.56	8.68

Group	Model[1]	Segment	Parameter estimate								Model selection[2]					
			c_1	c_2	c_3	z_1	z_2	z_3	T_1	T_2	K	log L	AIC$_{(c)}$	ΔAIC$_{(c)}$	BIC	ΔBIC
Levenson	(1)	1	1.252			0.129					3	24.46	-42.31	24.94	-37.64	22.95
(1981)	(2)	2	2.841			0.721	0.001		-2.443		5	37.32	-63.01	4.24	-55.82	4.77
Wisconsin	(3)	2	1.076			4.015	0.007		-3.336		5	37.84	-64.06	3.19	-56.87	3.72
forest	(4)	2	2.165	1.043		0.518	-0.017		-1.824		6	38.34	-62.35	4.90	-54.12	6.47
plants	(5)*	2	1.045			0.187			-0.781		4	19.87	-30.70	36.55	-24.70	35.89
(canopy	(6)*	2	1.252			0.129			-3.523		4	24.46	-39.87	27.38	-33.88	26.71
species)	(7)*	2	0.690	1.080		0.010			-2.444		5	26.45	-41.27	25.98	-34.09	26.50
	(8)#	2	2.841			0.721			-2.443		4	37.37	-65.68	1.57	-59.69	0.90
	(9)#	2	1.065			3.170			-3.282		4	37.82	-66.58	0.67	-60.59	0.00
	(10)#	2	2.166	1.071		0.518			-1.854		5	37.38	-63.13	4.12	-55.95	4.64
	(11)*	3	2.166	1.029		0.518	-0.030		-3.523	-1.854	7	37.57	-57.94	9.31	-48.81	11.78
	(12)*	3	0.690	1.304	1.108	0.132	0.074		-2.444	-1.372	8	28.00	-35.76	31.49	-25.91	34.68
	(13)	3	2.165	1.803	1.113	0.518	0.436	0.082	-1.824	-1.384	9	44.08	-64.71	2.54	-54.31	6.28
	(14)#	3	2.166	1.707	1.034	0.518	0.369		-1.854	-1.388	8	43.74	-67.25	0.00	-57.39	3.20
	(15)	3	2.166	1.043		0.518	-0.017		-1.854	-1.851	7	38.34	-59.49	7.76	-50.36	10.23
Levenson	(1)	1	1.288			0.012					3	30.38	-54.15	8.23	-49.48	3.05
(1981)	(2)	2	1.419			0.078	0.001		-1.801		5	31.10	-50.58	11.80	-43.40	9.13
Wisconsin	(3)	2	1.177			0.091	-0.074		-1.660		5	31.80	-51.99	10.39	-44.80	7.73
forest	(4)	2	1.525	1.394		0.120	0.159		-1.489		6	37.33	-60.33	2.05	-52.10	0.43
plants	(5)*	2	1.268			0.048			-0.941		4	30.35	-51.66	10.72	-45.66	6.87
(shrub	(6)*	2	1.207			-0.044			-1.652		4	30.58	-52.10	10.28	-46.11	6.42
species)	(7)*	2	1.299	1.394		0.159			-1.489		5	34.38	-57.13	5.25	-49.95	2.58
	(8)#	2	1.419			0.078			-1.801		4	31.12	-53.19	9.19	-47.20	5.33
	(9)#	2	1.278			0.076			-1.770		4	31.13	-53.20	9.18	-47.21	5.32
	(10)#	2	1.525	1.217		0.120			-1.506		5	35.59	-59.56	2.82	-52.38	0.15
	(11)*	3	1.736	1.394		0.241	0.159		-2.183	-1.506	7	38.50	-59.79	2.59	-50.67	1.86
	(12)*	3	1.252	-1.172	1.321	-1.567	0.070		-1.686	-1.388	8	40.58	-60.92	1.46	-51.07	1.46
	(13)	3	1.437	-1.397	1.321	0.085	-1.714	0.070	-1.660	-1.388	9	41.56	-59.67	2.71	-49.27	3.26
	(14)#	3	1.425	-0.194	1.246	0.081	-0.947		-1.703	-1.384	8	41.31	-62.38	0.00	-52.53	0.00
	(15)	3	1.525	1.394		0.120	0.159		-1.505	-1.490	7	37.33	-57.47	4.91	-48.34	4.19

Group	Model[1]	Segment	Parameter estimate								Model selection[2]					
			c_1	c_2	c_3	z_1	z_2	z_3	T_1	T_2	K	log L	$AIC_{(c)}$	$\Delta AIC_{(c)}$	BIC	ΔBIC
Levenson (1981) Wisconsin forest plants (total woody species)	(1)	1	1.431			0.022					3	45.23	-83.84	14.80	-79.18	12.28
	(2)	2	1.595			0.108	0.001		-1.686		5	48.81	-86.00	12.64	-78.81	12.65
	(3)	2	1.257			0.136	-0.116		-1.622		5	52.68	-93.74	4.90	-86.56	4.90
	(4)	2	1.687	1.346		0.145	-0.018		-1.489		6	55.19	-96.04	2.60	-87.81	3.65
	(5)*	2	1.398			0.001			-0.399		4	44.84	-80.62	18.02	-74.63	16.83
	(6)*	2	1.296			-0.074			-1.537		4	45.95	-82.85	15.79	-76.86	14.60
	(7)*	2	1.316	1.290		-0.086			-1.917		5	52.06	-92.51	6.13	-85.32	6.14
	(8)#	2	1.595			0.108			-1.686		4	48.88	-88.70	9.94	-82.71	8.75
	(9)#	2	1.412			0.108			-1.686		4	48.88	-88.70	9.94	-82.71	8.75
	(10)#	2	1.687	1.366		0.145			-1.506		5	55.13	-98.64	0.00	-91.46	0.00
	(11)*	3	1.781	1.346		0.198	-0.018		-2.823	-1.506	7	56.08	-94.96	3.68	-85.83	5.63
	(12)*	3	1.318	1.885	1.346	0.259	-0.018		-2.215	-1.489	8	55.97	-91.71	6.93	-81.86	9.60
	(13)	3	1.513	1.885	1.346	0.077	0.259	-0.018	-2.215	-1.489	9	56.86	-90.27	8.37	-79.87	11.59
	(14)#	3	1.583	0.915	1.357	0.104	-0.346		-1.686	-1.142	8	57.82	-95.40	3.24	-85.55	5.91
	(15)	3	1.687	1.353		0.145	-0.010		-1.503	-1.484	7	55.27	-93.34	5.30	-84.21	7.25
Lomba et al. (2013) Forest patches plants	(1)	1	1.627			-0.019					3	32.06	-57.61	6.38	-52.39	0.00
	(2)	2	1.388			0.108	-0.046		1.887		5	32.33	-53.29	10.70	-45.09	7.30
	(3)	2	2.580			0.069	-0.449		2.164		5	32.87	-54.38	9.61	-46.19	6.20
	(4)	2	2.991	1.930		-0.912	-0.161		1.772		6	36.49	-59.02	4.97	-49.50	2.89
	(5)*	2	1.589			0.001			2.490		4	32.04	-55.19	8.80	-48.43	3.96
	(6)*	2	2.428			-0.384			2.169		4	32.70	-56.51	7.48	-49.75	2.64
	(7)*	2	1.516	2.064		-0.223			1.829		5	34.68	-58.00	5.99	-49.80	2.59
	(8)#	2	1.388			0.108			1.887		4	32.17	-55.45	8.54	-48.69	3.70
	(9)#	2	1.591			0.105			1.887		4	32.17	-55.45	8.54	-48.69	3.70
	(10)#	2	3.124	1.593		-1.001			1.762		5	34.52	-57.68	6.31	-49.48	2.91
	(11)*	3	-3.195	1.788		2.631	-0.097		1.799	1.921	7	37.40	-58.14	5.85	-47.42	4.97
	(12)*	3	1.551	-1.201	1.834	1.531	-0.118		1.665	1.929	8	35.52	-51.53	12.46	-39.75	12.64
	(13)	3	3.577	-1.201	1.834	-1.303	1.531	-0.12	1.665	1.929	9	37.47	-52.44	11.55	-39.74	12.65
	(14)#	3	1.592	-1.525	1.593	0.057	1.697		1.580	1.762	8	41.75	-63.99	0.00	-52.21	0.18
	(15)	3	3.577	2.072		-1.303	-0.227		1.708	1.840	7	37.06	-57.45	6.54	-46.74	5.65

Group	Model[1]	Segment	Parameter estimate								Model selection[2]					
			c_1	c_2	c_3	z_1	z_2	z_3	T_1	T_2	K	log L	$AIC_{(c)}$	$\Delta AIC_{(c)}$	BIC	ΔBIC
Loyn	(1)	1	1.756			0.238					3	35.58	-64.71	6.77	-59.09	5.07
(1987)	(2)	2	1.841			0.297	0.068		-0.397		5	40.24	-69.28	2.20	-60.35	3.81
Victorian	(3)	2	1.747			0.293	0.072		-0.397		5	40.23	-69.27	2.21	-60.34	3.82
Forests	(4)	2	1.835	1.749		0.293	0.068		-0.301		6	40.25	-66.79	4.69	-56.35	7.81
birds	(5)*	2	1.079			0.241			-2.816		4	35.64	-62.49	8.99	-55.17	8.99
	(6)*	2	1.757			0.241			-2.816		4	35.64	-62.49	8.99	-55.17	8.99
	(7)*	2	1.040	1.748		0.224			-2.000		5	38.02	-64.84	6.64	-55.91	8.25
	(8)#	2	1.826			0.288			-0.130		4	40.13	-71.48	0.00	-64.16	0.00
	(9)#	2	1.788			0.288			-0.131		4	40.13	-71.48	0.00	-64.16	0.00
	(10)#	2	1.826	1.788		0.288			-0.244		5	40.13	-69.06	2.42	-60.13	4.03
	(11)*	3	1.861	1.747		0.315	0.072		-2.490	-0.398	7	41.01	-65.69	5.79	-53.85	10.31
	(12)*	3	1.040	1.830	1.749	0.287	0.068		-2.000	-0.301	8	42.22	-65.38	6.10	-52.24	11.92
	(13)	3	0.828	1.830	1.749	-0.084	0.287	0.068	-2.000	-0.301	9	42.31	-62.70	8.78	-48.39	15.77
	(14)#	3	1.079	1.838	1.788	0.000	0.301		-2.301	-0.244	8	42.20	-65.33	6.15	-52.20	11.96
	(15)	3	1.883	1.772		0.320	0.039		-1.183	-0.101	7	40.52	-64.70	6.78	-52.86	11.30
Morrision	(1)	1	3.567			0.619					3	-3.67	13.99	3.11	18.48	0.15
(2014)	(2)	2	2.180			0.340	1.154		-4.476		5	-0.55	12.81	1.93	19.67	1.34
Abaco	(3)	2	5.953			0.320	1.186		-4.476		5	-0.55	12.82	1.94	19.68	1.35
archipelag	(4)	2	3.309	5.884		0.553	1.168		-4.706		6	1.79	10.88	0.00	18.69	0.36
o plants	(5)*	2	0.486			1.050			-4.678		4	-1.74	12.58	1.70	18.33	0.00
(2012)	(6)*	2	5.784			1.144			-4.678		4	-1.96	13.03	2.15	18.78	0.45
	(7)*	2	0.505	3.559		0.593			-4.362		5	-0.29	12.29	1.41	19.15	0.82
	(8)#	2	3.519			0.609			-3.775		4	-3.68	16.47	5.59	22.21	3.88
	(9)#	2	1.322			0.706			-3.775		4	-4.27	17.64	6.76	23.39	5.06
	(10)#	2	1.776	1.082		0.262			-4.373		5	-1.92	15.56	4.68	22.41	4.08
	(11)*	3	3.655	5.884		0.624	1.168		-5.411	-4.751	7	1.88	13.63	2.75	22.23	3.90
	(12)*	3	0.405	-34.061	5.166	-7.305	0.994		-4.797	-4.612	8	4.21	12.08	1.20	21.28	2.95
	(13)	3	2.180	36.804	4.338	0.340	8.231	0.788	-4.475	-4.314	9	5.56	12.69	1.81	22.31	3.98
	(14)#	3	2.233	17.361	1.082	0.350	3.800		-4.491	-4.373	8	3.75	13.01	2.13	22.21	3.88
	(15)	3	3.309	5.884		0.553	1.168		-4.751	-4.706	7	1.79	13.81	2.93	22.41	4.08

Group	Model[1]	Segment	Parameter estimate								Model selection[2]					
			c_1	c_2	c_3	z_1	z_2	z_3	T_1	T_2	K	log L	AIC$_{(c)}$	ΔAIC$_{(c)}$	BIC	ΔBIC
Morrision	(1)	1	2.260			0.414					3	-14.11	34.66	8.76	40.39	6.30
(2014)	(2)	2	0.648			0.050	0.719		-3.932		5	-8.88	28.92	3.02	38.07	3.98
Andros	(3)	2	3.278			0.051	0.719		-3.932		5	-8.88	28.92	3.02	38.07	3.98
archipelag	(4)	2	0.757	2.460		0.074	0.438		-3.586		6	-7.11	27.87	1.97	38.59	4.50
o plants	(5)*	2	0.420			0.704			-3.996		4	-8.92	26.60	0.70	34.09	0.00
(1999)	(6)*	2	3.232			0.704			-3.996		4	-8.92	26.60	0.70	34.09	0.00
	(7)*	2	0.442	2.460		0.438			-3.586		5	-7.37	25.90	0.00	35.05	0.96
	(8)#	2	2.258			0.414			-2.326		4	-14.11	36.97	11.07	44.45	10.36
	(9)#	2	1.301			0.416			-2.326		4	-14.11	36.97	11.07	44.46	10.37
	(10)#	2	1.197	1.198		0.172			-3.380		5	-8.38	27.91	2.01	37.06	2.97
	(11)*	3	7.021	1.627		1.809	0.139		-3.643	-3.134	7	-6.45	29.14	3.24	41.32	7.23
	(12)*	3	0.448	37.618	2.460	10.279	0.438		-3.728	-3.586	8	-5.56	30.07	4.17	43.61	9.52
	(13)	3	0.954	37.618	2.460	0.117	10.279	0.438	-3.728	-3.586	9	-5.00	31.75	5.85	46.55	12.46
	(14)#	3	0.843	-15.852	1.198	0.093	-4.878		-3.531	-3.380	8	-4.87	28.69	2.79	42.23	8.14
	(15)	3	0.620	1.738		0.044	0.179		-3.641	-3.276	7	-6.09	28.43	2.53	40.61	6.52
Morrision	(1)	1	2.253			0.425					3	-13.37	33.19	9.47	38.92	7.71
(2014)	(2)	2	0.240			-0.026	0.716		-4.036		5	-7.49	26.13	2.41	35.28	4.07
Andros	(3)	2	3.179			-0.001	0.698		-4.036		5	-7.49	26.13	2.41	35.27	4.06
archipelag	(4)	2	0.838	2.324		0.105	0.408		-3.586		6	-6.96	27.58	3.86	38.29	7.08
o plants	(5)*	2	0.355			0.704			-4.036		4	-7.48	23.72	0.00	31.21	0.00
(2012)	(6)*	2	3.179			0.698			-4.036		4	-7.49	23.73	0.01	31.21	0.00
	(7)*	2	0.345	2.825		0.582			-3.848		5	-6.43	24.01	0.29	33.16	1.95
	(8)#	2	2.242			0.422			-2.326		4	-13.37	35.50	11.78	42.99	11.78
	(9)#	2	1.301			0.444			-2.326		4	-13.43	35.62	11.90	43.10	11.89
	(10)#	2	1.298	1.143		0.208			-3.380		5	-8.74	28.63	4.91	37.78	6.57
	(11)*	3	3.651	1.865		0.834	0.243		-3.943	-3.380	7	-5.70	27.65	3.93	39.83	8.62
	(12)*	3	0.345	-2.078	2.324	-0.713	0.408		-3.848	-3.586	8	-4.81	28.55	4.83	42.10	10.89
	(13)	3	0.094	-2.078	2.324	-0.057	-0.713	0.408	-3.848	-3.586	9	-4.71	31.17	7.45	45.97	14.76
	(14)#	3	1.034	2.871	1.143	0.140	0.620		-4.260	-3.380	8	-3.97	26.87	3.15	40.42	9.21
	(15)	3	0.277	1.586		-0.018	0.142		-3.937	-3.144	7	-5.59	27.42	3.70	39.60	8.39

Group	Model[1]	Segment	Parameter estimate								Model selection[2]					
			c_1	c_2	c_3	z_1	z_2	z_3	T_1	T_2	K	log L	$AIC_{(c)}$	$\Delta AIC_{(c)}$	BIC	ΔBIC
Morrison	(1)	1	2.034			0.473					3	-8.14	22.45	14.17	31.18	8.79
(2014)	(2)	2	-0.324			-0.084	0.568		-4.053		5	-1.14	12.72	4.44	27.13	4.74
Exuma	(3)	2	2.312			0.001	0.566		-4.020		5	-1.26	12.95	4.67	27.37	4.98
Cays	(4)	2	0.110	2.082		0.013	0.483		-3.636		6	1.08	10.45	2.17	27.65	5.26
plants	(5)*	2	0.038			0.566			-4.020		4	-1.26	10.80	2.52	22.39	0.00
	(6)*	2	2.312			0.566			-4.021		4	-1.26	10.80	2.52	22.39	0.00
	(7)*	2	0.056	2.082		0.483			-3.636		5	1.08	8.28	0.00	22.70	0.31
	(8)#	2	2.024			0.470			-1.953		4	-8.14	24.57	16.29	36.16	13.77
	(9)#	2	1.092			0.498			-2.078		4	-12.97	34.23	25.95	45.82	23.43
	(10)#	2	1.956	1.115		0.452			-2.502		5	-6.61	23.66	15.38	38.08	15.69
	(11)*	3	12.628	2.176		3.418	0.517		-3.676	-3.520	7	2.09	10.65	2.37	30.62	8.23
	(12)*	3	0.056	13.77	2.248	3.732	0.544		-3.636	-3.456	8	3.26	10.55	2.27	33.24	10.85
	(13)	3	0.110	13.77	2.248	0.013	3.732	0.544	-3.636	-3.456	9	3.27	12.81	4.53	38.19	15.80
	(14)#	3	0.025	1.968	1.115	-0.006	0.449		-3.656	-2.502	8	3.04	10.99	2.71	33.68	11.29
	(15)	3	0.005	2.082		-0.011	0.483		-3.651	-3.635	7	1.62	11.59	3.31	31.56	9.17
Niemelä et	(1)	1	1.267			0.342					3	-6.20	18.89	8.42	24.37	3.64
al. (1987)	(2)	2	1.423			0.419	0.000		-0.823		5	-4.63	20.51	10.04	29.20	8.47
Southern	(3)	2	1.034			0.404	-0.253		-0.544		5	-4.51	20.27	9.80	28.96	8.23
Fennoscan	(4)	2	0.671	1.150		0.100	0.120		-1.569		6	0.04	13.71	3.24	23.85	3.12
dia	(5)*	2	0.376			0.372			-2.488		4	-5.38	19.59	9.12	26.73	6.00
carabid	(6)*	2	1.302			0.372			-2.489		4	-5.38	19.59	9.12	26.73	6.00
beetles	(7)*	2	0.452	1.150		0.120			-1.569		5	-0.39	12.04	1.57	20.73	0.00
	(8)#	2	1.423			0.419			-0.822		4	-4.63	18.07	7.60	25.21	4.48
	(9)#	2	1.079			0.419			-0.823		4	-4.63	18.07	7.60	25.21	4.48
	(10)#	2	0.559	1.017		0.055			-1.620		5	-2.59	16.43	5.96	25.12	4.39
	(11)*	3	2.514	1.098		1.218	0.014		-1.703	-1.301	7	2.28	11.87	1.40	23.36	2.63
	(12)*	3	0.452	1.371	1.088	0.412	-0.011		-1.569	-1.292	8	1.74	15.72	5.25	28.43	7.70
	(13)	3	0.671	1.371	1.088	0.010	0.412	-0.01	-1.569	-1.292	9	2.21	17.67	7.20	31.48	10.75
	(14)#	3	0.655	2.857	1.087	0.092	1.451		-1.699	-1.301	8	4.37	10.47	0.00	23.18	2.45
	(15)	3	0.655	1.071		0.092	-0.053		-1.678	-1.194	7	2.56	11.32	0.85	22.81	2.08

Group	Model[1]	Segment	Parameter estimate								Model selection[2]					
			c_1	c_2	c_3	z_1	z_2	z_3	T_1	T_2	K	log L	AIC$_{(c)}$	ΔAIC$_{(c)}$	BIC	ΔBIC
Nikolić et	(1)	1	2.225			0.343					3	-25.01	56.25	24.05	64.00	14.92
al. (2008)	(2)	2	2.853			0.756	0.270		-1.148		5	-18.48	47.57	15.37	60.28	11.20
Adriatic	(3)	2	2.289			0.739	0.274		-1.148		5	-18.48	47.56	15.36	60.28	11.20
islands	(4)	2	-3.680	2.239		-2.014	0.321		-2.076		6	-12.30	37.44	5.24	52.57	3.49
plants	(5)*	2	1.473			0.382			-1.872		4	-29.62	67.64	35.44	77.90	28.82
	(6)*	2	2.225			0.343			-2.284		4	-25.01	58.41	26.21	68.67	19.59
	(7)*	2	0.693	2.239		0.321			-2.076		5	-12.88	36.36	4.16	49.08	0.00
	(8)#	2	2.270			0.383			1.592		4	-23.96	56.32	24.12	66.58	17.50
	(9)#	2	2.880			0.383			1.594		4	-23.96	56.32	24.12	66.58	17.50
	(10)#	2	2.270	2.880		0.383			1.348		5	-23.96	58.53	26.33	71.24	22.16
	(11)*	3	11.197	2.229		4.842	0.335		-2.154	-1.873	7	-15.74	46.62	14.42	64.13	15.05
	(12)*	3	0.693	-0.117	2.262	-0.906	0.298		-2.076	-1.658	8	-7.36	32.20	0.00	52.03	2.95
	(13)	3	-3.680	-0.117	2.262	-2.014	-0.906	0.298	-2.076	-1.658	9	-6.71	33.30	1.10	55.39	6.31
	(14)#	3	0.903	2.253	2.926	-	0.362		-2.149	1.885	8	-11.90	41.28	9.08	61.10	12.02
	(15)	3	-3.680	2.239		-2.014	0.321		-2.080	-2.076	7	-12.30	39.73	7.53	57.24	8.16
Panitsa et	(1)	1	1.003			0.243					3	-22.22	50.73	3.65	57.80	0.00
al. (2006)	(2)	2	1.236			0.371	0.001		-1.167		5	-20.40	51.55	4.47	63.07	5.27
Aegean	(3)	2	0.764			0.380	-0.044		-1.155		5	-20.37	51.50	4.42	63.02	5.22
Sea plants	(4)	2	1.358	1.072		0.431	0.490		-1.000		6	-17.36	47.78	0.70	61.44	3.64
(Graminea	(5)*	2	0.460			0.269			-2.096		4	-23.68	55.85	8.77	65.17	7.37
e)	(6)*	2	1.003			0.243			-3.301		4	-22.22	52.93	5.85	62.25	4.45
	(7)*	2	0.519	0.773		-0.038			-1.409		5	-22.19	55.14	8.06	66.66	8.86
	(8)#	2	1.236			0.371			-1.167		4	-20.40	49.29	2.21	58.61	0.81
	(9)#	2	0.798			0.366			-1.167		4	-20.39	49.28	2.20	58.61	0.81
	(10)#	2	1.349	0.747		0.427			-1.018		5	-19.26	49.28	2.20	60.80	3.00
	(11)*	3	1.367	1.012		0.439	0.382		-3.111	-1.018	7	-18.20	51.83	4.75	67.58	9.78
	(12)*	3	0.421	1.636	1.072	0.635	0.490		-2.046	-1.000	8	-17.69	53.24	6.16	71.01	13.21
	(13)	3	1.358	1.170	0.796	0.431	0.613	0.318	-1.000	-0.326	9	-17.08	54.52	7.44	74.24	16.44
	(14)#	3	1.121	-0.990	0.820	0.316	-1.678		-1.252	-0.870	8	-14.61	47.08	0.00	64.85	7.05
	(15)	3	1.358	1.075		0.431	0.496		-1.014	-0.999	7	-17.35	50.14	3.06	65.89	8.09

Group	Model[1]	Segment	Parameter estimate								Model selection[2]					
			c_1	c_2	c_3	z_1	z_2	z_3	T_1	T_2	K	log L	AIC$_{(c)}$	ΔAIC$_{(c)}$	BIC	ΔBIC
Panitsa et	(1)	1	0.887			0.119					3	-8.33	22.96	5.39	30.03	0.00
al. (2006)	(2)	2	1.117			0.247	0.001		-1.409		5	-6.42	23.58	6.01	35.11	5.08
Aegean	(3)	2	0.721			0.289	-0.061		-1.398		5	-6.22	23.18	5.61	34.71	4.68
Sea plants	(4)	2	1.146	0.834		0.258	0.115		-1.108		6	-6.00	25.07	7.50	38.73	8.70
(halophyte	(5)*	2	0.650			0.106			-2.046		4	-9.35	27.19	9.62	36.51	6.48
s)	(6)*	2	0.887			0.119			-3.301		4	-8.33	25.16	7.59	34.48	4.45
	(7)*	2	0.349	0.848		0.086			-2.699		5	-8.01	26.78	9.21	38.30	8.27
	(8)#	2	1.117			0.247			-1.409		4	-6.42	21.32	3.75	30.65	0.62
	(9)#	2	0.784			0.265			-1.409		4	-6.36	21.22	3.65	30.54	0.51
	(10)#	2	1.227	0.764		0.297			-1.284		5	-6.04	22.84	5.27	34.36	4.33
	(11)*	3	1.132	0.781		0.251	0.026		-3.301	-1.143	7	-6.47	28.37	10.80	44.11	14.08
	(12)*	3	0.628	-32.267	0.756	-19.696	-0.017		-1.699	-1.620	8	-4.98	27.83	10.26	45.59	15.56
	(13)	3	1.612	29.391	0.804	0.437	14.545	0.039	-2.046	-1.959	9	-4.41	29.19	11.62	48.92	18.89
	(14)#	3	1.858	-7.386	0.762	0.530	-3.817		-2.155	-2.000	8	0.15	17.57	0.00	35.33	5.30
	(15)	3	1.227	0.841		0.297	0.127		-1.288	-0.987	7	-5.71	26.85	9.28	42.60	12.57
Panitsa et	(1)	1	0.896			0.271					3	-25.00	56.30	3.67	63.37	0.00
al. (2006)	(2)	2	1.032			0.345	0.121		-1.167		5	-24.55	59.86	7.23	71.38	8.01
Aegean	(3)	2	-0.219			0.331	-2.609		-0.414		5	-24.35	59.44	6.81	70.96	7.59
Sea plants	(4)	2	1.180	1.098		0.419	0.686		-1.018		6	-21.11	55.28	2.65	68.94	5.57
(Legumin	(5)*	2	0.000			0.272			-3.299		4	-25.01	58.50	5.87	67.83	4.46
osae)	(6)*	2	0.896			0.271			-3.301		4	-25.00	58.50	5.87	67.83	4.46
	(7)*	2	0.298	0.831		0.202			-1.699		5	-25.72	62.19	9.56	73.71	10.34
	(8)#	2	0.910			0.280			-0.455		4	-24.78	58.05	5.42	67.37	4.00
	(9)#	2	0.771			0.271			-0.455		4	-24.79	58.07	5.44	67.39	4.02
	(10)#	2	0.938	0.656		0.296			-0.413		5	-24.42	59.59	6.96	71.11	7.74
	(11)*	3	1.496	1.098		0.641	0.686		-1.991	-1.046	7	-19.84	55.12	2.49	70.87	7.50
	(12)*	3	0.275	1.591	1.098	0.712	0.686		-2.046	-1.018	8	-19.51	56.89	4.26	74.65	11.28
	(13)	3	1.180	1.157	0.812	0.419	0.757	0.335	-1.018	-0.326	9	-21.01	62.38	9.75	82.10	18.73
	(14)#	3	1.032	-1.805	0.712	0.345	-2.374		-1.155	-0.854	8	-17.38	52.63	0.00	70.40	7.03
	(15)	3	1.180	1.098		0.419	0.686		-1.045	-1.018	7	-21.11	57.65	5.02	73.39	10.02

Group	Model[1]	Segment	Parameter estimate								Model selection[2]					
			c_1	c_2	c_3	z_1	z_2	z_3	T_1	T_2	K	log L	$AIC_{(c)}$	$\Delta AIC_{(c)}$	BIC	ΔBIC
Panitsa et al. (2006) Aegean Sea plants (therophytes)	(1)	1	1.810			0.439					3	-33.77	73.83	4.46	80.90	0.00
	(2)	2	2.042			0.568	0.157		-1.149		5	-32.19	75.13	5.76	86.65	5.75
	(3)	2	0.504			0.440	-2.468		-0.449		5	-31.74	74.23	4.86	85.76	4.86
	(4)	2	1.830	1.521		0.451	0.242		-0.326		6	-33.56	80.18	10.81	93.84	12.94
	(5)*	2	0.806			0.486			-2.154		4	-35.86	80.21	10.84	89.53	8.63
	(6)*	2	1.810			0.439			-3.301		4	-33.77	76.03	6.66	85.36	4.46
	(7)*	2	0.389	1.786		0.418			-2.699		5	-34.30	79.36	9.99	90.88	9.98
	(8)#	2	2.113			0.603			-1.107		4	-32.43	73.35	3.98	82.68	1.78
	(9)#	2	1.441			0.598			-1.107		4	-32.43	73.35	3.98	82.67	1.77
	(10)#	2	1.750	1.458		0.430			-1.398		5	-30.65	72.05	2.68	83.57	2.67
	(11)*	3	1.903	0.504		0.494	-2.468		-3.301	-0.413	7	-31.32	78.07	8.70	93.82	12.92
	(12)*	3	0.389	1.806	1.521	0.431	0.242		-2.699	-0.326	8	-34.14	86.14	16.77	103.91	23.01
	(13)	3	2.165	1.899	1.521	0.629	0.692	0.242	-1.000	-0.326	9	-30.26	80.88	11.51	100.61	19.71
	(14)#	3	2.938	2.690	1.458	0.869	1.035		-2.097	-1.398	8	-25.75	69.37	0.00	87.13	6.23
	(15)	3	1.903	12.575		0.494	36.720		-0.414	-0.346	7	-28.44	72.32	2.95	88.07	7.17
Panitsa et al. (2006) Aegean Sea plants (Total species)	(1)	1	2.019			0.397					3	-28.96	64.21	7.15	71.28	0.26
	(2)	2	2.322			0.564	0.040		-1.148		5	-26.56	63.87	6.81	75.39	4.37
	(3)	2	1.956			2.017	0.342		-2.808		5	-26.33	63.41	6.35	74.93	3.91
	(4)	2	2.043	0.411		1.709	0.278		-0.326		6	-28.61	70.29	13.23	83.95	12.93
	(5)*	2	1.183			0.411			-2.046		4	-32.24	72.97	15.91	82.29	11.27
	(6)*	2	2.019			0.397			-3.301		4	-28.96	66.41	9.35	75.74	4.72
	(7)*	2	0.602	1.967		0.352			-2.699		5	-29.42	69.60	12.54	81.12	10.10
	(8)#	2	2.342			0.573			-1.142		4	-26.60	61.70	4.64	71.02	0.00
	(9)#	2	1.681			0.566			-1.142		4	-26.60	61.70	4.64	71.02	0.00
	(10)#	2	2.391	1.656		0.598			-1.018		5	-26.18	63.11	6.05	74.63	3.61
	(11)*	3	2.096	0.567		0.443	-3.101		-3.301	-0.413	7	-26.79	69.02	11.96	84.76	13.74
	(12)*	3	1.183	0.000	1.967	-	0.351		-2.097	-2.000	8	-25.87	69.61	12.55	87.38	16.36
	(13)	3	2.891	12.463	1.908	0.776	5.794	0.288	-2.046	-1.959	9	-26.14	72.65	15.59	92.37	21.35
	(14)#	3	3.285	2.046	1.607	0.925	0.402		-2.155	-0.413	8	-19.59	57.06	0.00	74.82	3.80
	(15)	3	2.100	9.740		0.445	26.677		-0.413	-0.327	7	-24.98	65.40	8.34	81.15	10.13

Group	Model[1]	Segment	Parameter estimate								Model selection[2]					
			c_1	c_2	c_3	z_1	z_2	z_3	T_1	T_2	K	log L	$AIC_{(c)}$	$\Delta AIC_{(c)}$	BIC	ΔBIC
Powell &	(1)	1	0.397			0.168					3	-417.79	841.57	274.92	857.83	250.48
Henderson	(2)	2	0.242			0.075	0.371		-0.112		5	-285.13	580.25	13.60	607.35	0.00
(2012);	(3)	2	0.275			0.076	0.370		-0.111		5	-285.13	580.25	13.60	607.35	0.00
Gao &	(4)	2	0.267	0.296		0.086	0.355		-0.603		6	-284.21	580.42	13.770	612.94	5.59
Perry	(5)*	2	0.113			0.359			-0.496		4	-316.80	641.61	74.95	663.28	55.93
(2016 a, b)	(6)*	2	0.295			0.356			-0.515		4	-316.80	641.61	74.95	663.28	55.93
West	(7)*	2	0.113	0.292		0.358			-0.467		5	-316.76	643.53	76.87	670.62	63.27
Indies	(8)#	2	0.394			0.165			5.022		4	-417.90	843.81	277.15	865.49	258.14
herpetofau	(9)#	2	0.447			0.210			-0.542		4	-673.97	1355.9	789.28	1377.6	770.25
na	(10)#	2	0.331	1.226		0.122			1.670		5	-346.08	702.16	135.51	729.26	121.91
	(11)*	3	0.308	0.296		0.117	0.355		-2.495	-0.603	7	-281.59	577.17	10.52	615.11	7.76
	(12)*	3	0.026	0.321	0.296	0.128	0.355		-2.119	-0.603	8	-281.55	579.10	12.45	622.46	15.11
	(13)	3	0.242	0.363	0.179	0.076	0.167	0.428	-0.022	1.302	9	-274.33	566.65	0.00	615.43	8.08
	(14)#	3	0.268	0.298	2.362	0.087	0.344		-0.605	4.049	8	-280.11	576.22	9.56	619.57	12.22
	(15)	3	0.244	0.091		0.076	0.460		-0.318	1.125	7	-278.58	571.15	4.50	609.09	1.74
Reed	(1)	1	1.155			0.341					3	-5.36	17.08	0.00	23.60	0.00
(1981)	(2)	2	1.218			0.405	0.276		-0.046		5	-4.64	20.17	3.09	30.73	7.13
British	(3)	2	1.213			0.405	0.276		-0.041		5	-4.64	20.17	3.09	30.73	7.13
Islands	(4)	2	0.914	1.213		0.208	0.276		-0.730		6	-3.12	19.50	2.42	31.97	8.37
birds	(5)*	2	0.452			0.370			-1.853		4	-6.41	21.40	4.32	29.98	6.38
	(6)*	2	1.160			0.333			-2.097		4	-5.41	19.41	2.33	27.99	4.39
	(7)*	2	0.634	1.213		0.276			-0.730		5	-4.18	19.26	2.18	29.82	6.22
	(8)#	2	1.167			0.358			1.499		4	-5.08	18.75	1.67	27.32	3.72
	(9)#	2	1.707			0.360			1.499		4	-5.08	18.75	1.67	27.32	3.72
	(10)#	2	1.170	1.690		0.362			1.500		5	-5.06	21.02	3.94	31.57	7.97
	(11)*	3	0.707	1.205		0.034	0.285		-2.097	-0.750	7	-4.29	24.30	7.22	38.61	15.01
	(12)*	3	0.301	0.581	1.213	-0.088	0.276		-1.854	-0.730	8	-1.01	20.27	3.19	36.35	12.75
	(13)	3	0.914	-2.793	1.212	0.208	-5.358	0.278	-0.730	-0.676	9	-2.65	26.17	9.09	43.92	20.32
	(14)#	3	0.907	1.204	1.720	0.203	0.294		-0.860	1.803	8	-3.03	24.32	7.24	40.39	16.79
	(15)	3	0.907	1.213		0.203	0.276		-0.751	-0.737	7	-3.11	21.95	4.87	36.26	12.66

Group	Model[1]	Segment	Parameter estimate								Model selection[2]					
			c_1	c_2	c_3	z_1	z_2	z_3	T_1	T_2	K	log L	$AIC_{(c)}$	$\Delta AIC_{(c)}$	BIC	ΔBIC
Rusterholz	(1)	1	1.741			0.448					3	46.66	-86.86	12.90	-81.24	4.21
& Howe	(2)	2	1.804			0.475	0.300		-1.397		5	47.56	-83.92	15.84	-75.00	10.45
(1979)	(3)	2	1.765			0.126	0.463		-3.000		5	47.49	-83.77	15.99	-74.85	10.60
Burntside	(4)	2	1.619	1.517		0.404	0.255		-1.620		6	52.16	-90.60	9.16	-80.16	5.29
Lake birds	(5)*	2	0.360			0.466			-3.025		4	47.38	-85.98	13.78	-78.67	6.78
	(6)*	2	1.770			0.466			-3.025		4	47.38	-85.98	13.78	-78.67	6.78
	(7)*	2	0.376	1.760		0.460			-2.699		5	46.94	-82.68	17.08	-73.76	11.69
	(8)#	2	1.772			0.462			-1.008		4	47.34	-85.89	13.87	-78.57	6.88
	(9)#	2	1.306			0.462			-1.008		4	47.34	-85.89	13.87	-78.57	6.88
	(10)#	2	1.780	1.286		0.465			-1.102		5	47.12	-83.04	16.72	-74.12	11.33
	(11)*	3	1.762	1.611		0.466	0.341		-3.008	-1.678	7	49.03	-81.73	18.03	-69.89	15.56
	(12)*	3	0.376	1.626	1.517	0.406	0.255		-2.699	-1.620	8	52.13	-85.19	14.57	-72.06	13.39
	(13)	3	1.389	0.797	1.517	0.329	-0.036	0.255	-2.301	-1.620	9	60.84	-99.76	0.00	-85.45	0.00
	(14)#	3	1.008	1.838	1.286	0.206	0.498		-2.523	-1.102	8	53.80	-88.54	11.22	-75.40	10.05
	(15)	3	1.619	1.503		0.404	0.243		-1.641	-1.617	7	52.20	-88.06	11.70	-76.22	9.23
Scanlan	(1)	1	1.844			0.231					3	28.35	-50.31	6.66	-44.13	3.07
(1981)	(2)	2	1.976			0.304	0.002		-0.783		5	31.98	-52.96	4.01	-43.01	4.19
Alexandri	(3)	2	1.742			0.310	-0.003		-0.783		5	31.97	-52.94	4.03	-42.99	4.21
a Forest	(4)	2	1.837	1.725		0.237	0.038		-1.356		6	33.32	-53.21	3.76	-41.50	5.70
plants	(5)*	2	1.248			0.256			-2.443		4	27.63	-46.61	10.36	-38.51	8.69
	(6)*	2	1.844			0.231			-2.620		4	28.35	-48.04	8.93	-39.94	7.26
	(7)*	2	1.269	1.801		0.192			-2.071		5	29.28	-47.56	9.41	-37.61	9.59
	(8)#	2	1.976			0.304			-0.783		4	31.98	-55.30	1.67	-47.20	0.00
	(9)#	2	1.743			0.308			-0.783		4	31.97	-55.29	1.68	-47.19	0.01
	(10)#	2	1.804	1.689		0.220			-1.420		5	33.35	-55.70	1.27	-45.75	1.45
	(11)*	3	1.804	1.727		0.220	0.043		-2.620	-1.420	7	33.56	-51.19	5.78	-37.80	9.40
	(12)*	3	1.269	0.836	1.740	-0.318	0.076		-2.071	-1.433	8	36.27	-54.01	2.96	-39.02	8.18
	(13)	3	1.801	0.836	1.740	0.234	-0.318	0.076	-2.071	-1.433	9	36.89	-52.57	4.40	-36.08	11.12
	(14)#	3	1.485	1.290	1.689	0.103	-0.077		-2.086	-1.420	8	37.75	-56.97	0.00	-41.98	5.22
	(15)	3	1.759	1.725		0.199	0.038		-1.485	-1.303	7	33.72	-51.52	5.45	-38.12	9.08

Group	Model[1]	Segment	Parameter estimate								Model selection[2]					
			c_1	c_2	c_3	z_1	z_2	z_3	T_1	T_2	K	log L	AIC$_{(c)}$	ΔAIC$_{(c)}$	BIC	ΔBIC
Scanlan (1981) Minnesota Forest plants	(1)	1	1.734			0.154					3	25.50	-44.40	4.16	-39.65	2.23
	(2)	2	1.542			0.053	0.830		-1.382		5	28.76	-45.95	2.61	-38.61	3.27
	(3)	2	2.624			0.049	0.837		-1.382		5	28.76	-45.95	2.61	-38.61	3.27
	(4)	2	1.542	2.624		0.053	0.837		-1.387		6	28.77	-43.26	5.30	-34.83	7.05
	(5)*	2	1.446			0.891			-1.387		4	28.51	-47.99	0.57	-41.88	0.00
	(6)*	2	2.624			0.837			-1.387		4	28.21	-47.39	1.17	-41.28	0.60
	(7)*	2	1.444	2.241		0.477			-1.318		5	29.26	-46.94	1.62	-39.60	2.28
	(8)#	2	1.665			0.118			-0.964		4	25.31	-41.59	6.97	-35.48	6.40
	(9)#	2	1.526			0.149			-1.388		4	24.15	-39.28	9.28	-33.17	8.71
	(10)#	2	1.535	1.617		0.049			-1.380		5	26.32	-41.07	7.49	-33.72	8.16
	(11)*	3	1.832	2.882		0.211	1.078		-1.960	-1.419	7	30.84	-44.57	3.99	-35.19	6.69
	(12)*	3	1.418	0.309	2.241	-0.764	0.477		-1.684	-1.318	8	34.34	-48.56	0.00	-38.40	3.48
	(13)	3	1.355	0.309	2.241	-0.032	-0.764	0.477	-1.684	-1.318	9	34.39	-45.48	3.08	-34.72	7.16
	(14)#	3	1.361	0.587	1.617	-0.030	-0.578		-1.686	-1.380	8	33.58	-47.06	1.50	-36.90	4.98
	(15)	3	1.660	2.882		0.112	1.078		-1.419	-1.388	7	30.05	-43.00	5.56	-33.62	8.26
Sfenthourakis & Triantis (2009) Aegean Sea isopod (the largest island removed)	(1)	1	8.671			4.884					3	-236.24	478.78	86.59	485.96	74.38
	(2)	2	5.492			1.320	8.118		0.221		5	-200.35	411.42	19.23	423.14	11.56
	(3)	2	-1.496			1.928	10.836		0.867		5	-197.44	405.61	13.42	417.33	5.75
	(4)	2	6.185	-0.965		1.891	10.585		0.914		6	-197.29	407.61	15.42	421.52	9.94
	(5)*	2	4.047			7.899			-0.044		4	-202.93	414.34	22.15	423.82	12.24
	(6)*	2	4.399			7.898			-0.044		4	-202.93	414.34	22.15	423.82	12.24
	(7)*	2	4.077	4.521		7.832			-0.244		5	-202.73	416.20	24.01	427.92	16.34
	(8)#	2	8.604			4.772			2.679		4	-236.36	481.22	89.02	490.69	79.11
	(9)#	2	21.667			6.243			1.871		4	-257.87	524.23	132.04	533.71	122.13
	(10)#	2	8.179	27.667		4.129			2.294		5	-217.50	445.72	53.53	457.44	45.86
	(11)*	3	5.211	-6.075		6.687	13.485		-0.178	2.294	7	-190.07	395.54	3.35	411.58	0.00
	(12)*	3	4.083	5.213	-6.075	6.683	13.485		-0.301	2.294	8	-190.11	398.03	5.83	416.14	4.56
	(13)	3	6.179	5.213	-6.075	1.755	6.683	13.48	-0.301	2.294	9	-185.95	392.19	0.00	412.31	0.73
	(14)#	3	6.179	5.213	27.667	1.755	6.683		-0.301	2.295	8	-190.41	398.62	6.43	416.73	5.15
	(15)	3	5.219	-18.835		1.118	18.334		-0.000	2.043	7	-190.48	396.36	4.17	412.40	0.82

Group	Model[1]	Segment	Parameter estimate								Model selection[2]					
			c_1	c_2	c_3	z_1	z_2	z_3	T_1	T_2	K	log L	$AIC_{(c)}$	$\Delta AIC_{(c)}$	BIC	ΔBIC
Sfenthoura	(1)	1	8.714			5.008					3	-239.87	486.03	88.70	493.26	76.51
kis &	(2)	2	5.492			1.320	8.074		0.227		5	-202.33	415.38	18.04	427.16	10.41
Triantis	(3)	2	1.943			1.694	9.023		0.548		5	-201.75	414.22	16.89	426.01	9.26
(2009)	(4)	2	6.185	1.445		1.891	9.256		0.914		6	-201.40	415.83	18.50	429.81	13.06
Aegean	(5)[*]	2	4.047			7.817			-0.055		4	-204.84	418.15	20.82	427.68	10.93
Sea isopod	(6)[*]	2	4.482			7.815			-0.055		4	-204.84	418.15	20.82	427.68	10.93
	(7)[*]	2	4.077	4.577		7.767			-0.244		5	-204.62	419.96	22.62	431.75	15.00
	(8)[#]	2	8.671			4.884			3.917		4	-240.01	488.50	91.16	498.03	81.28
	(9)[#]	2	22.316			6.483			1.871		4	-267.15	542.78	145.44	552.31	135.56
	(10)[#]	2	8.179	28.571		4.129			2.294		5	-221.62	453.97	56.63	465.75	49.00
	(11)[*]	3	5.211	15.126		6.687	4.972		-0.178	2.294	7	-192.62	400.61	3.27	416.75	0.00
	(12)[*]	3	4.083	5.213	15.126	6.683	4.972		-0.301	2.294	8	-192.65	403.10	5.76	421.32	4.57
	(13)	3	6.179	5.213	15.126	1.755	6.683	4.972	-0.301	2.294	9	-188.54	397.34	0.00	417.59	0.84
	(14)[#]	3	6.179	5.213	28.571	1.755	6.683		-0.301	2.295	8	-192.04	401.87	4.53	420.09	3.34
	(15)	3	5.154	1.245		1.072	9.349		-0.134	1.076	7	-199.01	413.39	16.05	429.52	12.77
Simaiakis	(1)	1	7.912			2.739					3	-209.4	425.12	30.56	431.40	26.43
et al.	(2)	2	8.041			1.964	22.175		2.581		5	-191.9	394.84	0.28	404.97	0.00
(2012)	(3)	2	-43.29			2.051	21.947		2.581		5	-192.0	394.93	0.37	405.06	0.09
Aegean	(4)	2	8.033	41.000		2.221	-		3.149		6	-197.8	408.97	14.41	420.91	15.94
Sea	(5)[*]	2	6.909			9.019			1.297		4	-200.3	409.30	14.74	417.55	12.58
centipedes	(6)[*]	2	-4.788			9.018			1.297		4	-200.3	409.30	14.74	417.55	12.58
	(7)[*]	2	6.897	-4.064		8.712			1.075		5	-200.3	411.49	16.93	421.62	16.65
	(8)[#]	2	8.033			2.221			3.917		4	-210.2	429.03	34.47	437.28	32.31
	(9)[#]	2	14.478			3.017			1.869		4	-216.1	440.80	46.24	449.04	44.07
	(10)[#]	2	8.041	27.667		1.964			2.634		5	-202.3	415.60	21.04	425.73	20.76
	(11)[*]	3	3.123	-32.02		5.268	18.662		0.617	2.084	7	-189.3	394.56	0.00	408.23	3.26
	(12)[*]	3	6.850	2.655	-32.02	5.541	18.662		0.248	2.115	8	-188.7	395.92	1.36	411.24	6.27
	(13)	3	8.832	2.655	-32.02	2.024	5.541	18.66	0.248	2.115	9	-187.0	395.02	0.46	411.90	6.93
	(14)[#]	3	8.007	102.24	27.667	1.867	-35.23		2.443	2.634	8	-190.1	398.62	4.06	413.93	8.96
	(15)	3	7.219	-43.29		0.918	21.95		0.620	2.633	7	-190.4	396.61	2.05	410.28	5.31

Group	Model[1]	Segment	Parameter estimate								Model selection[2]					
			c_1	c_2	c_3	z_1	z_2	z_3	T_1	T_2	K	log L	$AIC_{(c)}$	$\Delta AIC_{(c)}$	BIC	ΔBIC
Simaiakis	(1)	1	8.408			5.227					3	-278.9	564.15	40.79	571.30	32.00
et al.	(2)	2	5.296			1.693	8.663		0.239		5	-263.4	537.59	14.23	549.24	9.94
(2012)	(3)	2	3.633			1.695	8.662		0.239		5	-263.4	537.59	14.23	549.24	9.94
Italian	(4)	2	8.073	-14.84		4.025	13.98		1.707		6	-262.5	538.03	14.67	551.86	12.56
islands	(5)*	2	3.143			8.437			-0.114		4	-264.2	536.94	13.58	546.37	7.07
centipedes	(6)*	2	4.107			8.436			-0.114		4	-264.2	536.94	13.58	546.37	7.07
	(7)*	2	3.342	3.860		8.554			0.050		5	-264.7	540.13	16.77	551.79	12.49
	(8)#	2	8.278			4.825			4.409		4	-279.4	567.35	43.99	576.77	37.47
	(9)#	2	47.000			8.510			4.409		4	-303.8	616.08	92.72	625.51	86.21
	(10)#	2	8.070	49.000		4.195			3.939		5	-265.0	540.67	17.31	552.32	13.02
	(11)*	3	5.269	-14.85		8.166	13.98		-0.399	1.667	7	-254.0	523.36	0.00	539.30	0.00
	(12)*	3	3.342	4.524	-14.84	8.442	13.98		0.050	1.707	8	-256.5	530.80	7.44	548.79	9.49
	(13)	3	8.162	-39.79	-11.90	4.127	27.69	13.23	1.667	1.833	9	-261.5	543.25	19.89	563.24	23.94
	(14)#	3	5.143	6.338	49.000	1.594	6.117		0.279	3.939	8	-257.9	533.63	10.27	551.63	12.33
	(15)	3	4.973	-112.6		1.480	36.75		-0.134	3.909	7	-258.3	532.04	8.68	547.98	8.68
Suarez et	(1)	1	1.089			0.381					3	7.72	-8.78	1.87	-4.38	0.65
al. (1998)	(2)	2	0.221			-0.120	0.444		-1.623		5	9.66	-7.56	3.09	-0.88	4.15
California	(3)	2	1.119			0.001	0.413		-1.787		5	9.89	-8.02	2.63	-1.34	3.69
ants	(4)	2	0.221	1.136		-0.120	0.441		-1.623		6	9.66	-4.78	5.87	2.81	7.84
	(5)*	2	0.451			0.444			-1.546		4	9.58	-10.02	0.63	-4.41	0.62
	(6)*	2	1.119			0.413			-1.787		4	9.90	-10.65	0.00	-5.03	0.00
	(7)*	2	0.301	1.119		0.413			-1.788		5	10.07	-8.38	2.27	-1.70	3.33
	(8)#	2	1.086			0.378			0.006		4	7.72	-6.30	4.35	-0.69	4.34
	(9)#	2	1.114			0.399			0.006		4	7.67	-6.19	4.46	-0.58	4.45
	(10)#	2	1.074	1.080		0.369			-0.141		5	7.39	-3.01	7.64	3.67	8.70
	(11)*	3	1.232	1.203		0.506	1.790		-1.778	-0.352	7	12.61	-7.73	2.92	0.59	5.62
	(12)*	3	0.301	1.158	1.208	0.445	1.897		-1.788	-0.249	8	11.29	-1.93	8.72	6.94	11.97
	(13)	3	0.221	1.200	1.208	-0.120	0.496	1.897	-1.623	-0.249	9	11.03	1.95	12.60	11.15	16.18
	(14)#	3	-1.764	1.232	1.000	-0.987	0.506		-1.788	-0.352	8	13.63	-6.62	4.03	2.25	7.28
	(15)	3	-1.764	1.119		-0.987	0.413		-1.787	-1.624	7	12.82	-8.13	2.52	0.19	5.22

Group	Model[1]	Segment	Parameter estimate								Model selection[2]					
			c_1	c_2	c_3	z_1	z_2	z_3	T_1	T_2	K	log L	$AIC_{(c)}$	$\Delta AIC_{(c)}$	BIC	ΔBIC
Vignoli et	(1)	1	0.503			0.316					3	20.35	-34.29	10.24	-28.33	4.97
al. (2009)	(2)	2	0.290			0.162	0.443		-0.725		5	24.49	-37.90	6.63	-28.34	4.96
Rome	(3)	2	0.496			0.168	0.439		-0.725		5	24.49	-37.90	6.63	-28.34	4.96
metropolis	(4)	2	0.209	0.583		0.111	0.260		-0.492		6	29.03	-44.53	0.00	-33.30	0.00
amphibian	(5)*	2	0.000			0.367			-1.387		4	22.64	-36.57	7.96	-28.76	4.54
s	(6)*	2	0.509			0.367			-1.387		4	22.64	-36.57	7.96	-28.76	4.54
	(7)*	2	0.065	0.583		0.260			-0.492		5	26.96	-42.85	1.68	-33.29	0.01
	(8)#	2	0.498			0.312			1.201		4	20.34	-31.98	12.55	-24.17	9.13
	(9)#	2	0.954			0.346			1.201		4	19.63	-30.57	13.96	-22.76	10.54
	(10)#	2	0.234	0.613		0.127			-0.526		5	17.61	-24.16	20.37	-14.59	18.71
	(11)*	3	0.242	0.545		0.134	0.334		-1.797	-0.526	7	26.15	-36.22	8.31	-23.40	9.90
	(12)*	3	0.041	-0.154	0.583	-0.334	0.260		-1.094	-0.492	8	28.79	-38.86	5.67	-24.56	8.74
	(13)	3	0.421	-0.154	0.583	0.243	-0.334	0.260	-1.094	-0.492	9	31.27	-41.07	3.46	-25.39	7.91
	(14)#	3	0.234	0.557	0.795	0.127	0.391		-0.526	0.585	8	29.53	-40.34	4.19	-26.04	7.26
	(15)	3	0.209	0.583		0.111	0.260		-0.526	-0.493	7	29.03	-41.98	2.55	-29.17	4.13
Vignoli et	(1)	1	0.799			0.395					3	14.96	-23.50	4.61	-17.54	1.00
al. (2009)	(2)	2	0.964			0.514	0.337		-0.971		5	16.67	-22.27	5.84	-12.70	5.84
Rome	(3)	2	0.800			0.557	0.313		-0.971		5	16.77	-22.47	5.64	-12.90	5.64
metropolis	(4)	2	0.041	0.798		-0.011	0.323		-1.439		6	19.59	-25.65	2.46	-14.42	4.12
reptiles	(5)*	2	0.086			0.403			-1.772		4	15.01	-21.32	6.79	-13.51	5.03
	(6)*	2	0.800			0.403			-1.772		4	15.01	-21.32	6.79	-13.51	5.03
	(7)*	2	0.060	0.798		0.323			-1.439		5	19.59	-28.11	0.00	-18.54	0.00
	(8)#	2	0.859			0.443			0.348		4	16.39	-24.07	4.04	-16.27	2.27
	(9)#	2	1.013			0.443			0.347		4	16.39	-24.07	4.04	-16.27	2.27
	(10)#	2	0.832	1.008		0.423			-0.049		5	16.71	-22.34	5.77	-12.78	5.76
	(11)*	3	1.542	0.789		0.934	0.348		-1.607	-0.943	7	20.58	-25.09	3.02	-12.28	5.76
	(12)*	3	0.060	-0.635	0.800	-0.703	0.313		-1.439	-1.260	8	20.14	-21.56	6.55	-7.26	11.28
	(13)	3	0.937	0.528	0.907	0.498	-0.030	0.161	-1.094	-0.164	9	20.52	-19.58	8.53	-3.89	14.65
	(14)#	3	0.742	0.492	0.973	0.383	-0.094		-1.110	-0.164	8	21.88	-25.05	3.06	-10.75	7.79
	(15)	3	0.041	0.798		-0.011	0.323		-1.484	-1.439	7	19.59	-23.11	5.00	-10.29	8.25

Group	Model[1]	Segment	Parameter estimate								Model selection[2]					
			c_1	c_2	c_3	z_1	z_2	z_3	T_1	T_2	K	log L	$AIC_{(c)}$	$\Delta AIC_{(c)}$	BIC	ΔBIC
Wang et	(1)	1	1.664			0.144					3	56.87	-107.11	1.52	-102.53	0.00
al. (2010)	(2)	2	0.748			-0.271	0.154		-2.174		5	59.42	-107.18	1.45	-100.16	2.37
Thousand	(3)	2	1.671			0.001	0.153		-2.042		5	58.73	-105.79	2.84	-98.77	3.76
Island	(4)	2	0.924	1.668		-0.198	0.145		-2.013		6	60.79	-107.17	1.46	-99.15	3.38
Lake birds	(5)*	2	1.364			0.150			-2.042		4	58.72	-108.36	0.27	-102.49	0.04
	(6)*	2	1.671			0.153			-2.042		4	58.74	-108.40	0.23	-102.53	0.00
	(7)*	2	1.356	1.666		0.137			-1.900		5	59.60	-107.54	1.09	-100.51	2.02
	(8)#	2	1.652			0.138			1.110		4	56.73	-104.39	4.24	-98.52	4.01
	(9)#	2	1.863			0.157			1.110		4	56.26	-103.45	5.18	-97.58	4.95
	(10)#	2	1.652	1.863		0.138			0.156		5	57.17	-102.67	5.96	-95.64	6.89
	(11)*	3	-1.803	1.669		-1.513	0.149		-2.093	-2.018	7	59.98	-102.68	5.95	-93.81	8.72
	(12)*	3	1.361	-0.680	1.668	-0.983	0.145		-2.066	-2.013	8	60.42	-100.48	8.15	-90.94	11.59
	(13)	3	1.013	-0.680	1.668	-0.159	-0.983	0.145	-2.066	-2.013	9	61.05	-98.47	10.16	-88.46	14.07
	(14)#	3	0.959	1.660	1.863	-0.183	0.143		-2.018	0.156	8	63.36	-106.35	2.28	-96.82	5.71
	(15)	3	0.736	1.667		-0.281	0.142		-2.017	-2.014	7	62.96	-108.63	0.00	-99.76	2.77
Wang et	(1)	1	0.665			0.204					3	14.57	-22.51	1.29	-17.93	0.00
al. (2010)	(2)	2	-0.646			-0.401	0.218		-2.136		5	15.61	-19.55	4.25	-12.53	5.40
Thousand	(3)	2	0.675			0.001	0.214		-2.082		5	15.21	-18.75	5.05	-11.73	6.20
Island	(4)	2	-1.242	0.663		-0.661	0.183		-1.996		6	19.10	-23.80	0.00	-15.77	2.16
Lake	(5)*	2	0.232			0.213			-2.082		4	15.21	-21.34	2.46	-15.47	2.46
lizards	(6)*	2	0.675			0.214			-2.082		4	15.21	-21.34	2.46	-15.47	2.46
	(7)*	2	0.176	0.663		0.183			-1.996		5	17.06	-22.45	1.35	-15.43	2.50
	(8)#	2	0.699			0.222			0.358		4	14.90	-20.71	3.09	-14.84	3.09
	(9)#	2	0.778			0.222			0.360		4	14.90	-20.71	3.09	-14.84	3.09
	(10)#	2	0.574	0.683		0.159			-0.491		5	14.92	-18.17	5.63	-11.15	6.78
	(11)*	3	-4.451	0.663		-2.214	0.183		-2.133	-2.013	7	19.33	-21.36	2.44	-12.49	5.44
	(12)*	3	0.259	-4.451	0.663	-2.214	0.183		-2.137	-1.996	8	19.35	-18.33	5.47	-8.79	9.14
	(13)	3	-1.829	-6.541	0.663	-0.897	-3.234	0.183	-2.174	-1.996	9	21.54	-19.46	4.34	-9.45	8.48
	(14)#	3	-1.242	0.361	0.683	-0.661	0.017		-2.013	-0.491	8	19.75	-19.14	4.66	-9.61	8.32
	(15)	3	-1.242	0.663		-0.661	0.183		-2.013	-1.996	7	19.10	-20.91	2.89	-12.04	5.89

Group	Model[1]	Segment	Parameter estimate								Model selection[2]					
			c_1	c_2	c_3	z_1	z_2	z_3	T_1	T_2	K	log L	$AIC_{(c)}$	$\Delta AIC_{(c)}$	BIC	ΔBIC
Wang et	(1)	1	0.846			0.301					3	11.74	-16.94	1.86	-11.88	0.00
al. (2015)	(2)	2	0.296			0.079	0.323		-2.377		5	13.04	-14.66	4.14	-6.73	5.15
Thousand	(3)	2	0.879			0.076	0.325		-2.377		5	13.05	-14.66	4.14	-6.74	5.14
Island	(4)	2	0.299	0.850		0.087	0.275		-1.996		6	15.44	-16.83	1.97	-7.65	4.23
Lake	(5)*	2	0.000			0.322			-2.720		4	12.96	-16.98	1.82	-10.43	1.45
snakes	(6)*	2	0.875			0.322			-2.721		4	12.96	-16.98	1.82	-10.43	1.45
	(7)*	2	0.099	0.850		0.275			-1.996		5	15.12	-18.80	0.00	-10.88	1.00
	(8)#	2	0.864			0.311			0.804		4	11.83	-14.74	4.06	-8.18	3.70
	(9)#	2	1.114			0.311			0.801		4	11.83	-14.74	4.06	-8.18	3.70
	(10)#	2	0.864	1.114		0.311			0.156		5	11.83	-12.24	6.56	-4.31	7.57
	(11)*	3	0.679	0.856		0.276	0.291		-2.299	-2.081	7	15.00	-13.21	5.59	-2.91	8.97
	(12)*	3	0.065	-12.604	0.850	-6.236	0.275		-2.081	-1.996	8	19.24	-18.78	0.02	-7.50	4.38
	(13)	3	0.168	-12.604	0.850	0.043	-6.236	0.275	-2.081	-1.996	9	19.31	-15.89	2.91	-3.78	8.10
	(14)#	3	0.299	0.866	1.114	0.087	0.284		-2.013	0.156	8	15.48	-11.27	7.53	0.00	11.88
	(15)	3	0.299	0.850		0.087	0.275		-2.013	-1.996	7	15.44	-14.07	4.73	-3.78	8.10
Welter-	(1)	1	14.500			9.638					3	-258.34	523.07	118.04	529.20	110.91
Schultes	(2)	2	14.574			6.831	91.832		2.863		5	-214.16	439.35	34.32	449.20	30.91
&	(3)	2	-228.70			6.839	91.800		2.863		5	-214.16	439.35	34.32	449.20	30.91
Williams	(4)	2	14.574	-228.70		6.831	91.800		2.925		6	-214.16	441.78	36.75	453.38	35.09
(1999)	(5)*	2	10.067			27.521			1.176		4	-234.35	477.37	72.34	485.40	67.11
Aegean	(6)*	2	-22.303			27.519			1.176		4	-234.35	477.37	72.34	485.40	67.11
Sea land	(7)*	2	10.194	-22.880		27.749			1.190		5	-234.36	479.74	74.71	489.59	71.30
snails	(8)#	2	14.700			7.754			3.916		4	-260.23	529.13	124.10	537.16	118.87
	(9)#	2	82.333			19.999			3.145		4	-300.92	610.50	205.47	618.53	200.24
	(10)#	2	14.700	135.00		7.754			3.212		5	-220.21	451.44	46.41	461.29	43.00
	(11)*	3	-2.127	-279.72		16.390	105.71		0.723	3.146	7	-197.27	410.51	5.48	423.77	5.48
	(12)*	3	9.714	-4.130	135.00	17.615	-		0.778	3.212	8	-196.84	412.26	7.23	427.08	8.79
	(13)	3	11.426	-3.309	135.00	2.570	17.276	-	1.246	3.212	9	-194.26	409.79	4.76	426.09	7.80
	(14)#	3	11.439	-4.664	135.00	2.582	17.847		1.236	3.212	8	-193.98	406.54	1.51	421.36	3.07
	(15)	3	10.969	-326.3		2.152	117.77		0.956	3.211	7	-194.50	405.03	0.00	418.29	0.00

Group	Model[1]	Segment	Parameter estimate								Model selection[2]					
			c_1	c_2	c_3	z_1	z_2	z_3	T_1	T_2	K	log L	AIC$_{(c)}$	ΔAIC$_{(c)}$	BIC	ΔBIC
Woinarski	(1)	1	0.329			0.206					3	7.53	-8.52	10.70	-3.37	9.19
et al.	(2)	2	0.162			0.047	0.401		0.053		5	14.41	-17.42	1.80	-9.35	3.21
(1999a)	(3)	2	0.144			0.047	0.401		0.053		5	14.41	-17.42	1.80	-9.35	3.21
Australian	(4)	2	0.224	0.167		0.091	0.382		-0.284		6	14.54	-15.07	4.14	-5.72	6.84
Islands	(5)*	2	0.114			0.401			-0.073		4	14.06	-19.22	0.00	-12.56	0.00
mammals	(6)*	2	0.144			0.401			-0.073		4	14.06	-19.22	0.00	-12.56	0.00
(Terrestria	(7)*	2	0.118	0.167		0.382			-0.284		5	13.72	-16.04	3.18	-7.97	4.59
l)	(8)#	2	0.314			0.187			2.321		4	7.20	-5.50	13.72	1.16	13.72
	(9)#	2	0.841			0.275			1.614		4	2.46	3.99	23.21	10.65	23.21
	(10)#	2	0.194	0.704		0.073			0.575		5	10.27	-9.15	10.07	-1.08	11.48
	(11)*	3	0.576	0.167		0.594	0.382		-0.832	-0.284	7	16.49	-16.24	2.98	-5.73	6.83
	(12)*	3	0.080	0.473	0.167	0.422	0.382		-1.041	-0.284	8	16.01	-12.41	6.81	-0.88	11.68
	(13)	3	0.011	0.473	0.167	-0.045	0.422	0.382	-1.041	-0.284	9	16.07	-9.52	9.70	2.89	15.45
	(14)#	3	0.224	0.153	0.704	0.091	0.241		-0.284	0.575	8	10.83	-2.06	17.16	9.48	22.04
	(15)	3	0.271	0.118		0.124	0.420		-0.284	-0.260	7	16.69	-16.64	2.58	-6.13	6.43
Woinarski	(1)	1	0.214			0.155					3	17.30	-28.06	3.94	-22.92	1.16
et al.	(2)	2	0.108			0.057	0.245		-0.149		5	20.24	-29.09	2.91	-21.02	3.06
(1999a)	(3)	2	0.133			0.054	0.247		-0.149		5	20.24	-29.09	2.91	-21.02	3.06
Australian	(4)	2	0.154	0.390		0.096	0.089		0.593		6	20.22	-26.44	5.56	-17.09	6.99
Islands	(5)*	2	0.036			0.232			-0.502		4	19.83	-30.74	1.26	-24.08	0.00
mammals	(6)*	2	0.153			0.232			-0.502		4	19.83	-30.74	1.26	-24.08	0.00
(Bats)	(7)*	2	0.062	0.131		0.244			0.384		5	17.43	-23.46	8.54	-15.39	8.69
	(8)#	2	0.201			0.139			2.321		4	16.97	-25.03	6.97	-18.37	5.71
	(9)#	2	0.527			0.179			1.614		4	15.53	-22.15	9.85	-15.49	8.59
	(10)#	2	0.125	0.499		0.072			0.575		5	20.88	-30.37	1.63	-22.31	1.77
	(11)*	3	-0.144	-0.071		0.859	0.336		0.248	0.928	7	24.21	-31.70	0.30	-21.18	2.90
	(12)*	3	0.046	-0.144	-0.071	0.859	0.336		0.190	1.103	8	24.64	-29.69	2.31	-18.15	5.93
	(13)	3	0.154	0.674	0.903	0.096	-0.143	-	0.593	1.973	9	23.82	-25.02	6.98	-12.61	11.47
	(14)#	3	0.125	0.493	0.903	0.072	-0.028		0.575	1.973	8	25.80	-32.00	0.00	-20.46	3.62
	(15)	3	0.125	0.408		0.072	0.079		0.561	0.591	7	22.12	-27.51	4.49	-17.00	7.08

Group	Model[1]	Segment	Parameter estimate								Model selection[2]					
			c_1	c_2	c_3	z_1	z_2	z_3	T_1	T_2	K	log L	$AIC_{(c)}$	$\Delta AIC_{(c)}$	BIC	ΔBIC
Woinarski	(1)	1	0.125			0.104					3	36.62	-66.82	52.42	-60.96	44.35
et al.	(2)	2	0.065			0.048	0.721		1.320		5	58.91	-106.70	12.54	-97.34	7.97
(1999b)	(3)	2	-0.822			0.048	0.720		1.319		5	58.91	-106.70	12.54	-97.34	7.97
Australian	(4)	2	0.067	-0.844		0.050	0.730		1.359		6	58.50	-103.41	15.83	-92.42	12.89
Islands	(5)[*]	2	0.000			0.328			0.284		4	57.06	-105.40	13.84	-97.75	7.56
frogs	(6)[*]	2	-0.093			0.328			0.284		4	57.06	-105.40	13.84	-97.75	7.56
	(7)[*]	2	0.006	-0.105		0.334			0.504		5	55.59	-100.06	19.18	-90.70	14.61
	(8)[#]	2	0.101			0.080			2.321		4	35.13	-61.53	57.71	-53.88	51.43
	(9)[#]	2	0.251			0.138			0.383		4	23.59	-38.46	80.78	-30.81	74.50
	(10)[#]	2	0.082	0.619		0.063			1.913		5	47.69	-84.28	34.96	-74.92	30.39
	(11)[*]	3	0.016	-0.105		0.101	0.334		-0.293	0.575	7	56.20	-96.25	22.99	-83.74	21.57
	(12)[*]	3	0.000	-0.212	-0.342	0.754	0.471		0.486	0.593	8	64.01	-109.19	10.05	-95.26	10.05
	(13)	3	0.000	-0.212	-0.342	0.000	0.754	0.471	0.486	0.593	9	64.01	-106.41	12.83	-91.16	14.15
	(14)[#]	3	0.000	0.045	0.954	0.000	0.191		0.486	1.973	8	69.03	-119.24	0.00	-105.31	0.00
	(15)	3	0.000	-2.269		0.000	1.396		0.063	1.912	7	66.37	-116.59	2.65	-104.08	1.23
Woinarski	(1)	1	0.758			0.285					3	18.08	-29.74	3.08	-23.88	0.00
et al.	(2)	2	-1.367			-0.673	0.298		-2.185		5	19.79	-28.46	4.36	-19.10	4.78
(1999b)	(3)	2	0.971			0.317	0.127		0.928		5	19.23	-27.34	5.48	-17.98	5.90
Australian	(4)	2	0.110	0.773		-0.021	0.264		-1.495		6	21.98	-30.37	2.45	-19.39	4.49
Islands	(5)[*]	2	0.151			0.294			-2.060		4	19.07	-29.41	3.41	-21.76	2.12
lizards	(6)[*]	2	0.757			0.294			-2.059		4	19.07	-29.41	3.41	-21.76	2.12
	(7)[*]	2	0.151	0.773		0.264			-1.495		5	21.96	-32.82	0.00	-23.46	0.42
	(8)[#]	2	0.778			0.304			1.483		4	18.91	-29.10	3.72	-21.45	2.43
	(9)[#]	2	1.228			0.304			1.480		4	18.91	-29.10	3.72	-21.45	2.43
	(10)[#]	2	0.778	1.228		0.304			1.359		5	18.91	-26.71	6.11	-17.35	6.53
	(11)[*]	3	-1.765	0.770		-1.130	0.268		-1.683	-1.538	7	23.28	-30.41	2.41	-17.91	5.97
	(12)[*]	3	0.151	0.718	0.931	0.213	0.151		-1.495	0.391	8	24.08	-29.34	3.48	-15.40	8.48
	(13)	3	0.110	0.718	0.931	-0.021	0.213	0.151	-1.495	0.391	9	24.09	-26.59	6.23	-11.34	12.54
	(14)[#]	3	-0.190	0.786	1.228	-0.159	0.290		-1.538	1.359	8	23.89	-28.95	3.87	-15.02	8.86
	(15)	3	-0.190	0.773		-0.159	0.264		-1.517	-1.488	7	23.45	-30.75	2.07	-18.24	5.64

Group	Model[1]	Segment	Parameter estimate								Model selection[2]					
			c_1	c_2	c_3	z_1	z_2	z_3	T_1	T_2	K	log L	AIC$_{(c)}$	ΔAIC$_{(c)}$	BIC	ΔBIC
Woinarski et al. (1999b) Australian Islands reptiles	(1)	1	0.786			0.307					3	17.97	-29.51	2.40	-23.65	0.00
	(2)	2	-1.367			-0.673	0.323		-2.158		5	20.17	-29.23	2.68	-19.87	3.78
	(3)	2	0.783			-0.673	0.323		-2.158		5	20.17	-29.23	2.68	-19.87	3.78
	(4)	2	0.110	0.798		-0.021	0.294		-1.495		6	21.52	-29.46	2.45	-18.48	5.17
	(5)[*]	2	0.151			0.320			-1.978		4	19.35	-29.97	1.94	-22.32	1.33
	(6)[*]	2	0.784			0.320			-1.977		4	19.35	-29.97	1.94	-22.32	1.33
	(7)[*]	2	0.151	0.798		0.294			-1.495		5	21.51	-31.91	0.00	-22.55	1.10
	(8)[#]	2	0.799			0.319			1.690		4	18.24	-27.74	4.17	-20.09	3.56
	(9)[#]	2	1.338			0.319			1.690		4	18.24	-27.74	4.17	-20.09	3.56
	(10)[#]	2	0.802	1.317		0.321			1.614		5	18.22	-25.32	6.59	-15.96	7.69
	(11)[*]	3	-1.765	0.796		-1.130	0.297		-1.683	-1.538	7	22.90	-29.64	2.27	-17.13	6.52
	(12)[*]	3	0.151	0.729	0.937	0.225	0.199		-1.495	0.391	8	23.48	-28.15	3.76	-14.21	9.44
	(13)	3	0.110	0.729	0.937	-0.021	0.225	0.199	-1.495	0.391	9	23.50	-25.40	6.51	-10.15	13.50
	(14)[#]	3	-0.190	0.810	1.317	-0.159	0.316		-1.538	1.359	8	23.08	-27.34	4.57	-13.41	10.24
	(15)	3	-0.190	0.798		-0.159	0.294		-1.528	-1.487	7	22.97	-29.79	2.12	-17.29	6.36
Woinarski et al. (1999b) Australian Islands snakes	(1)	1	0.185			0.145					3	25.66	-44.90	44.01	-39.04	35.94
	(2)	2	0.000			0.000	0.317		-0.154		5	40.58	-70.04	18.87	-60.68	14.30
	(3)	2	0.049			0.001	0.317		-0.150		5	40.58	-70.04	18.87	-60.68	14.30
	(4)	2	0.124	0.212		0.090	0.241		1.326		6	34.46	-55.33	33.58	-44.35	30.63
	(5)[*]	2	0.000			0.317			-0.154		4	40.58	-72.43	16.48	-64.78	10.20
	(6)[*]	2	0.049			0.317			-0.153		4	40.58	-72.43	16.48	-64.78	10.20
	(7)[*]	2	0.007	0.049		0.316			0.384		5	38.67	-66.23	22.68	-56.87	18.11
	(8)[#]	2	0.167			0.128			2.321		4	25.09	-41.46	47.45	-33.81	41.17
	(9)[#]	2	0.628			0.196			1.972		4	20.23	-31.73	57.18	-24.08	50.90
	(10)[#]	2	0.103	0.579		0.073			1.103		5	32.24	-53.37	35.54	-44.01	30.97
	(11)[*]	3	0.056	-0.995		0.308	0.819		-0.183	1.913	7	41.41	-66.67	22.24	-54.17	20.81
	(12)[*]	3	0.007	0.123	0.212	0.194	0.241		0.384	1.326	8	39.35	-59.87	29.04	-45.94	29.04
	(13)	3	0.031	0.123	0.212	0.021	0.194	0.241	0.384	1.326	9	39.66	-57.71	31.20	-42.46	32.52
	(14)[#]	3	0.000	0.385	0.579	0.000	-0.253		0.190	1.103	8	53.87	-88.91	0.00	-74.98	0.00
	(15)	3	0.093	0.495		0.065	0.103		1.103	1.326	7	43.28	-70.41	18.50	-57.90	17.08

Group	Model[1]	Segment	Parameter estimate								Model selection[2]					
			c_1	c_2	c_3	z_1	z_2	z_3	T_1	T_2	K	log L	AIC$_{(c)}$	ΔAIC$_{(c)}$	BIC	ΔBIC
Woinarski	(1)	1	0.146			0.100					3	13.30	-20.15	21.43	-14.47	13.77
et al.	(2)	2	0.017			0.007	0.258		0.039		5	18.40	-25.63	15.95	-16.59	11.65
(2000)	(3)	2	0.007			0.007	0.258		0.037		5	18.40	-25.63	15.95	-16.59	11.65
Australian	(4)	2	0.087	0.666		0.054	-0.074		1.326		6	18.62	-23.55	18.03	-12.97	15.27
Islands	(5)*	2	0.008			0.258			0.002		4	18.38	-28.00	13.58	-20.60	7.64
plants	(6)*	2	0.007			0.258			0.001		4	18.38	-28.00	13.58	-20.60	7.64
(Introduce	(7)*	2	0.042	0.666		-0.074			1.326		5	16.07	-20.96	20.62	-11.93	16.31
d)	(8)#	2	0.133			0.089			2.321		4	13.10	-17.43	24.15	-10.02	18.22
	(9)#	2	0.349			0.102			1.971		4	12.98	-17.18	24.40	-9.78	18.46
	(10)#	2	0.068	0.465		0.039			1.103		5	18.14	-25.10	16.48	-16.06	12.18
	(11)*	3	-3.717	-1.226		3.355	0.844		1.101	1.359	7	25.69	-35.10	6.48	-23.09	5.15
	(12)*	3	0.031	-3.717	-1.226	3.355	0.844		1.103	1.614	8	29.01	-39.02	2.56	-25.68	2.56
	(13)	3	0.068	-3.717	-1.226	0.039	3.355	0.844	1.103	1.614	9	30.74	-39.65	1.93	-25.09	3.15
	(14)#	3	0.068	-3.717	0.425	0.039	3.355		1.103	1.359	8	30.29	-41.58	0.00	-28.24	0.00
	(15)	3	0.061	0.705		0.035	-0.093		1.104	1.326	7	22.45	-28.61	12.97	-16.60	11.64
Woinarski	(1)	1	0.256			0.130					3	10.77	-15.09	11.63	-9.41	9.91
et al.	(2)	2	0.161			0.056	0.465		0.739		5	18.74	-26.30	0.42	-17.26	2.06
(2000)	(3)	2	-0.141			0.056	0.465		0.741		5	18.74	-26.30	0.42	-17.26	2.06
Australian	(4)	2	0.154	0.168		0.051	0.300		1.103		6	19.97	-26.25	0.47	-15.68	3.64
Islands	(5)*	2	0.092			0.406			0.319		4	17.75	-26.72	0.00	-19.32	0.00
plants	(6)*	2	-0.038			0.406			0.320		4	17.75	-26.72	0.00	-19.32	0.00
(Threatene	(7)*	2	0.088	0.021		0.370			0.450		5	18.11	-25.05	1.67	-16.01	3.31
d)	(8)#	2	0.234			0.111			2.321		4	10.28	-11.79	14.93	-4.39	14.93
	(9)#	2	0.389			0.160			0.383		4	3.25	2.27	28.99	9.67	28.99
	(10)#	2	0.154	0.666		0.051			0.928		5	18.29	-25.40	1.32	-16.36	2.96
	(11)*	3	-0.738	-1.341		1.054	1.039		0.831	1.359	7	19.05	-21.82	4.90	-9.80	9.52
	(12)*	3	0.082	3.260	0.028	11.727	0.361		-0.287	-0.222	8	21.27	-23.55	3.17	-10.20	9.12
	(13)	3	0.154	0.433	-1.341	0.051	0.159	1.039	1.103	1.614	9	23.21	-24.60	2.12	-10.04	9.28
	(14)#	3	0.154	0.558	1.079	0.051	0.025		0.928	1.973	8	21.72	-24.44	2.28	-11.10	8.22
	(15)	3	0.154	0.168		0.051	0.300		0.929	1.103	7	19.97	-23.65	3.07	-11.63	7.69

[1] * refers to models that have a leftmost horizontal segment; # refers to models that have a rightmost horizontal segment.

[2] AIC and ΔAIC were applied for Worldwide terrestrial mammals and West Indies herpetofauna; and AIC_c and ΔAIC_c were applied for the rest groups.
