# Supplementary material for: On piecewise models and species–area patterns
Source: Ecol Evol. 2019 Jul 2;9(14):8351–61. doi: 10.1002/ece3.5417 (PMC6662316; doi:10.1002/ece3.5417)
Supplement: Supplementary file 2 [file ECE3-9-8351-s002.pdf]

Ecology and Evolution

SUPPORTING INFORMATION

On piecewise models and species–area patterns

De Gao, Zhen Cao, Peng Xu, Gad Perry

Appendix S2 Supplementary results, including Fig. S1–S15.

Figure S1. Results of Model (1), linear regression analyses for six sample datasets in accordance with Fig. 2.

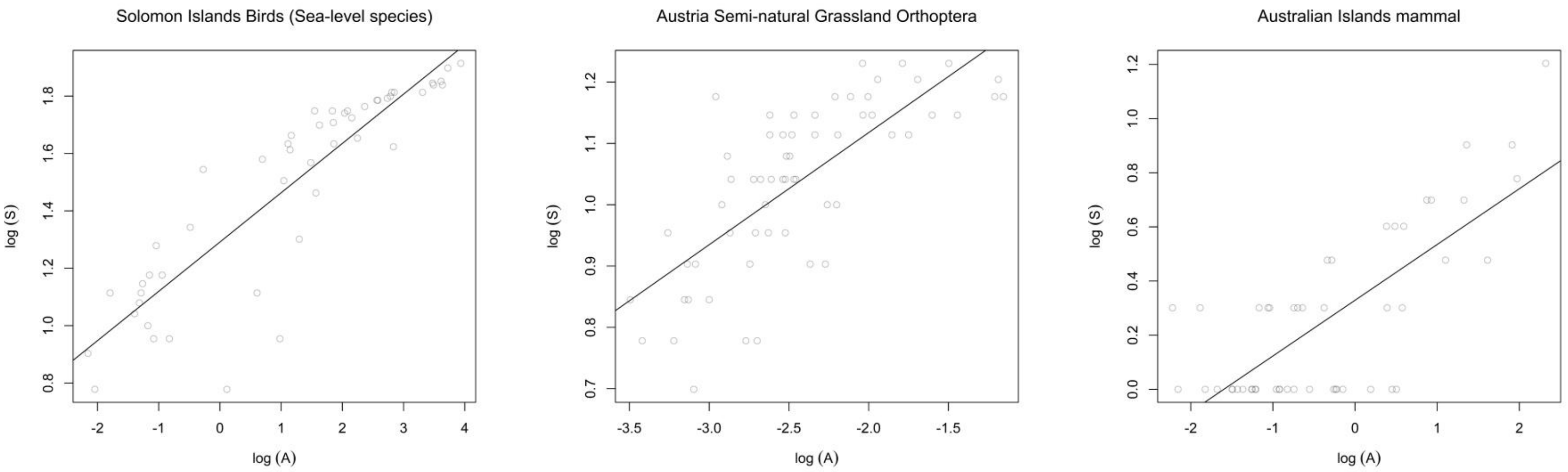

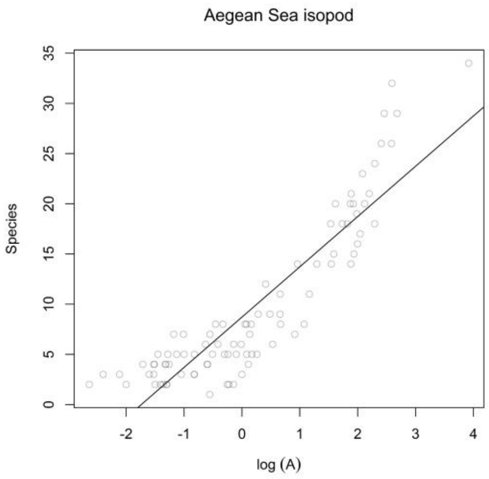
Aegean Sea isopod
Species
0
5
10
15
20
25
30
35
-2
-1
0
1
2
3
4
log (A)

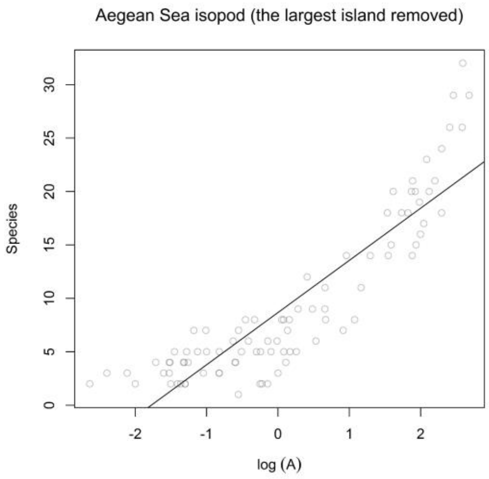
Aegean Sea isopod (the largest island removed)
Species
0
5
10
15
20
25
30
-2
-1
0
1
2
log (A)

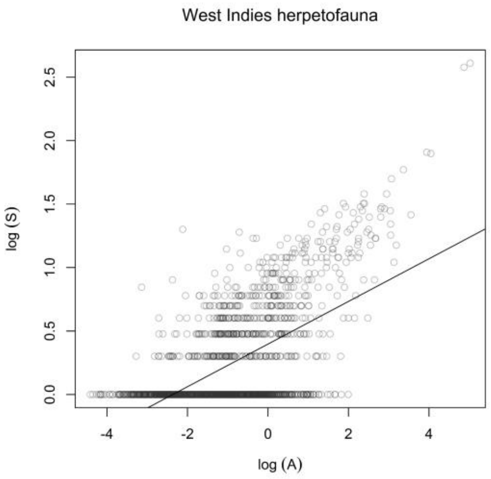
West Indies herpetofauna
log (S)
0.0
0.5
1.0
1.5
2.0
2.5
-4
-2
0
2
4
log (A)

Figure S2. Results and the double iterative processes used in Model (2) regression analyses for six sample datasets in accordance with Fig. 2. The breakpoint (T_1) that returns a minimal residual sum of squares (RSS) was chosen. After T_1 was determined, we run iterative process of z_2 again to look for the z_2 that produced the minimum RSS value.

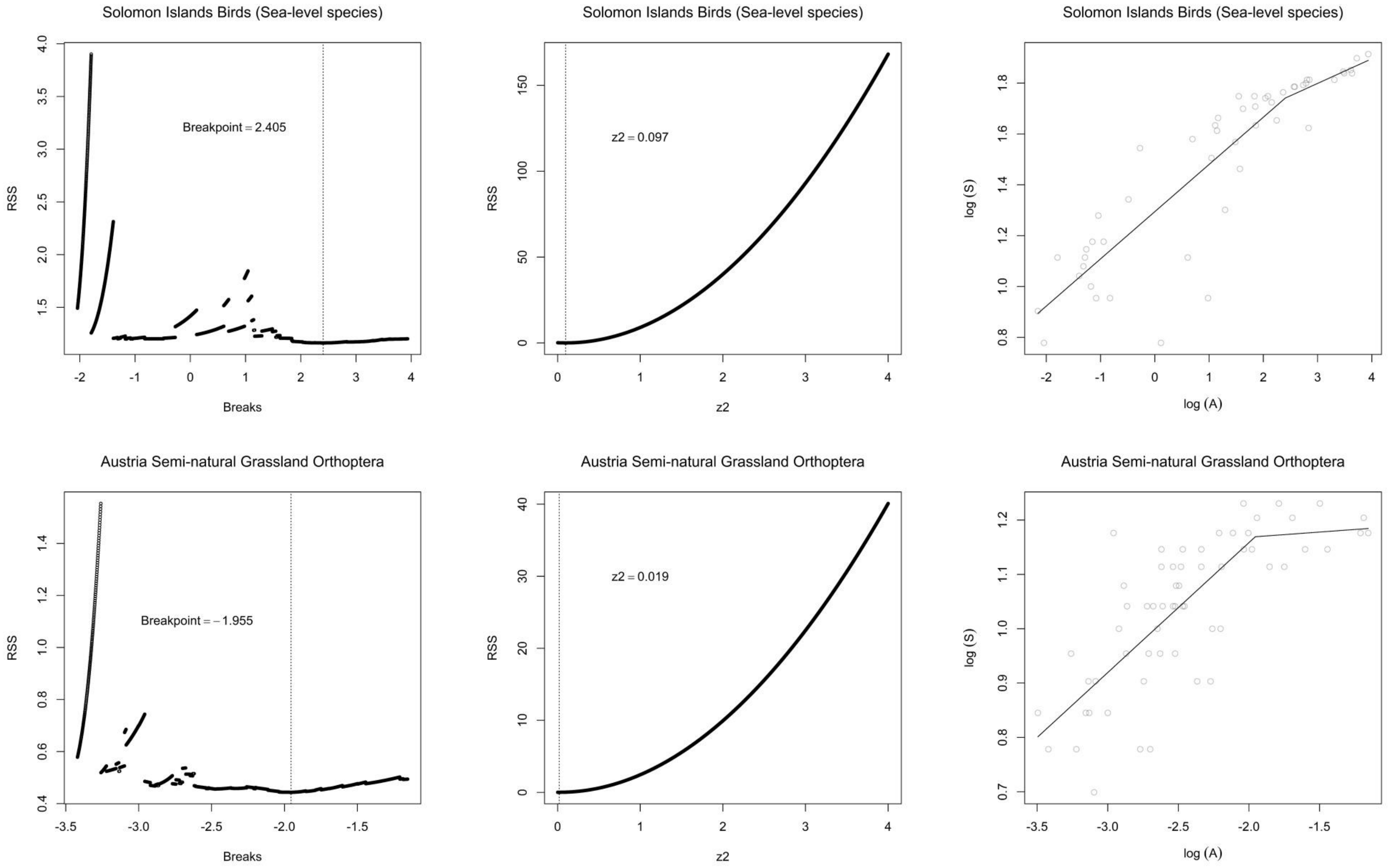

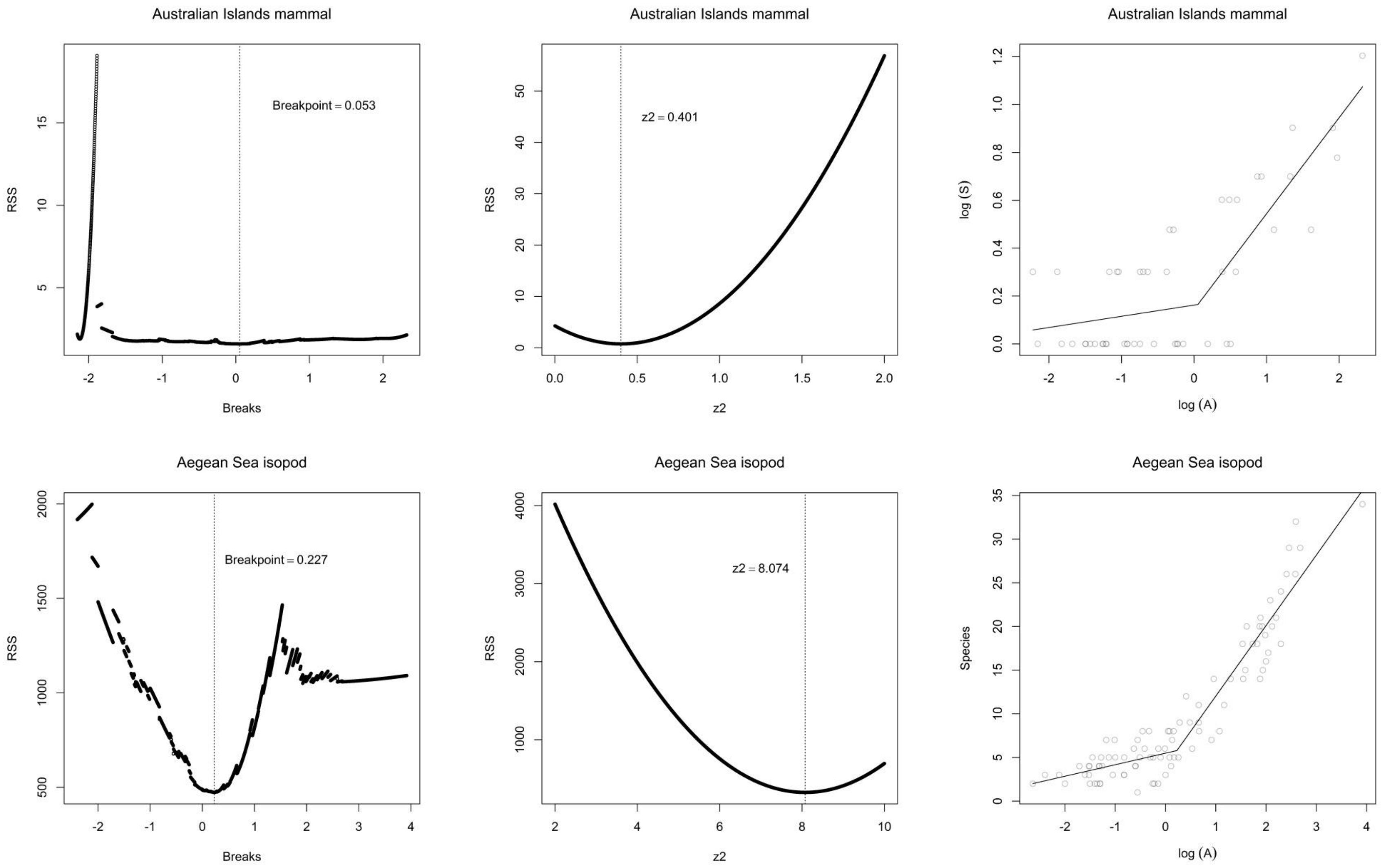
Australian Islands mammal
RSS
Breaks
Breakpoint = 0.053
Australian Islands mammal
RSS
z2
z2 = 0.401
Australian Islands mammal
log (S)
log (A)
Aegean Sea isopod
RSS
Breaks
Breakpoint = 0.227
Aegean Sea isopod
RSS
z2
z2 = 8.074
Aegean Sea isopod
Species
log (A)

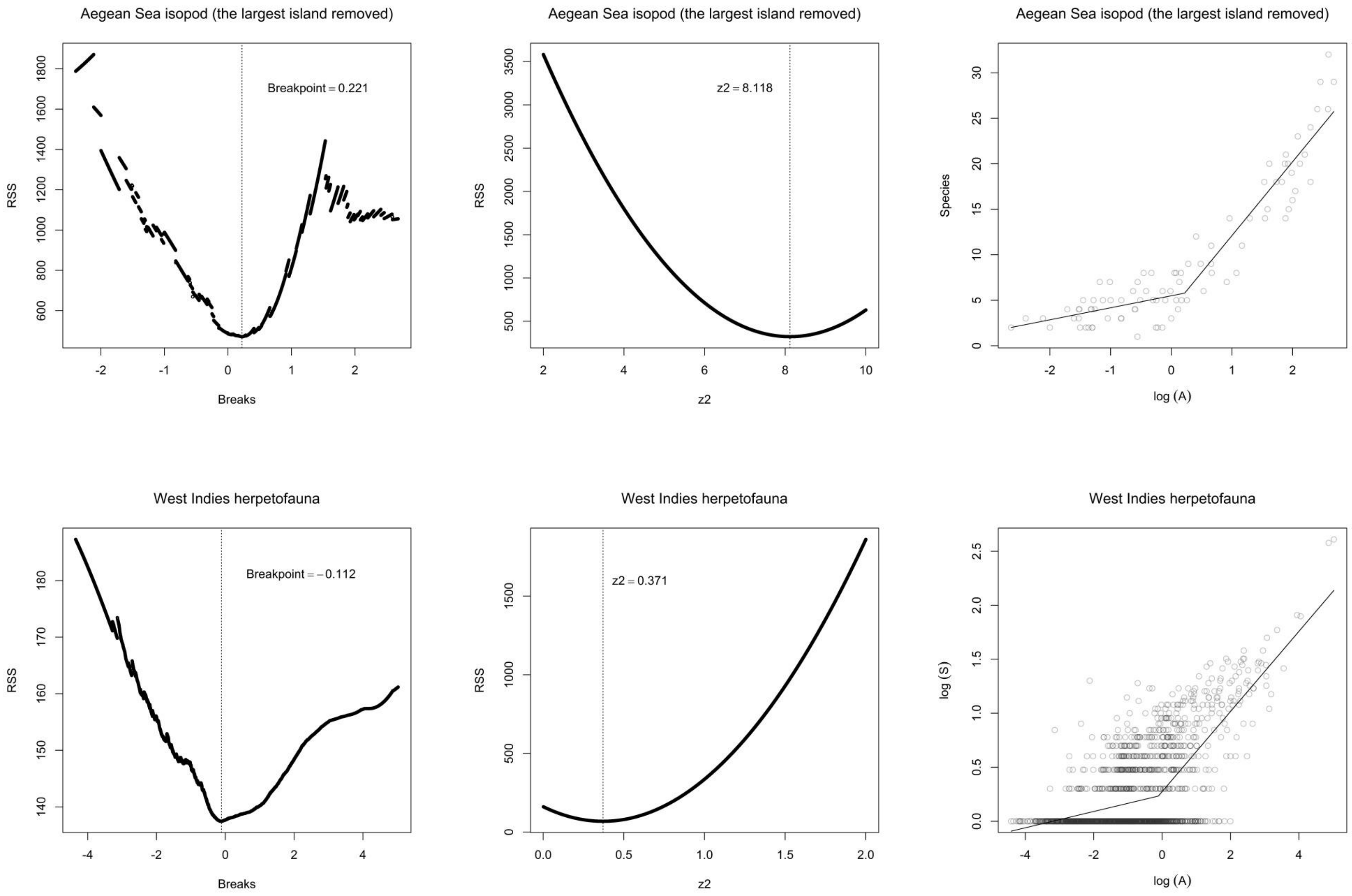
Aegean Sea isopod (the largest island removed)
Breakpoint = 0.221
RSS
Breaks
Aegean Sea isopod (the largest island removed)
z2 = 8.118
RSS
z2
Aegean Sea isopod (the largest island removed)
Species
log (A)
West Indies herpetofauna
Breakpoint = −0.112
RSS
Breaks
West Indies herpetofauna
z2 = 0.371
RSS
z2
West Indies herpetofauna
log (S)
log (A)

Figure S3. Results and the double iterative processes used in Model (3) regression analyses for six sample datasets in accordance with Fig. 2. The breakpoint (T_1) that returns a minimal residual sum of squares (RSS) was chosen. After T_1 was determined, we run iterative process of z_1 again to look for the z_1 that produced the minimum RSS value.

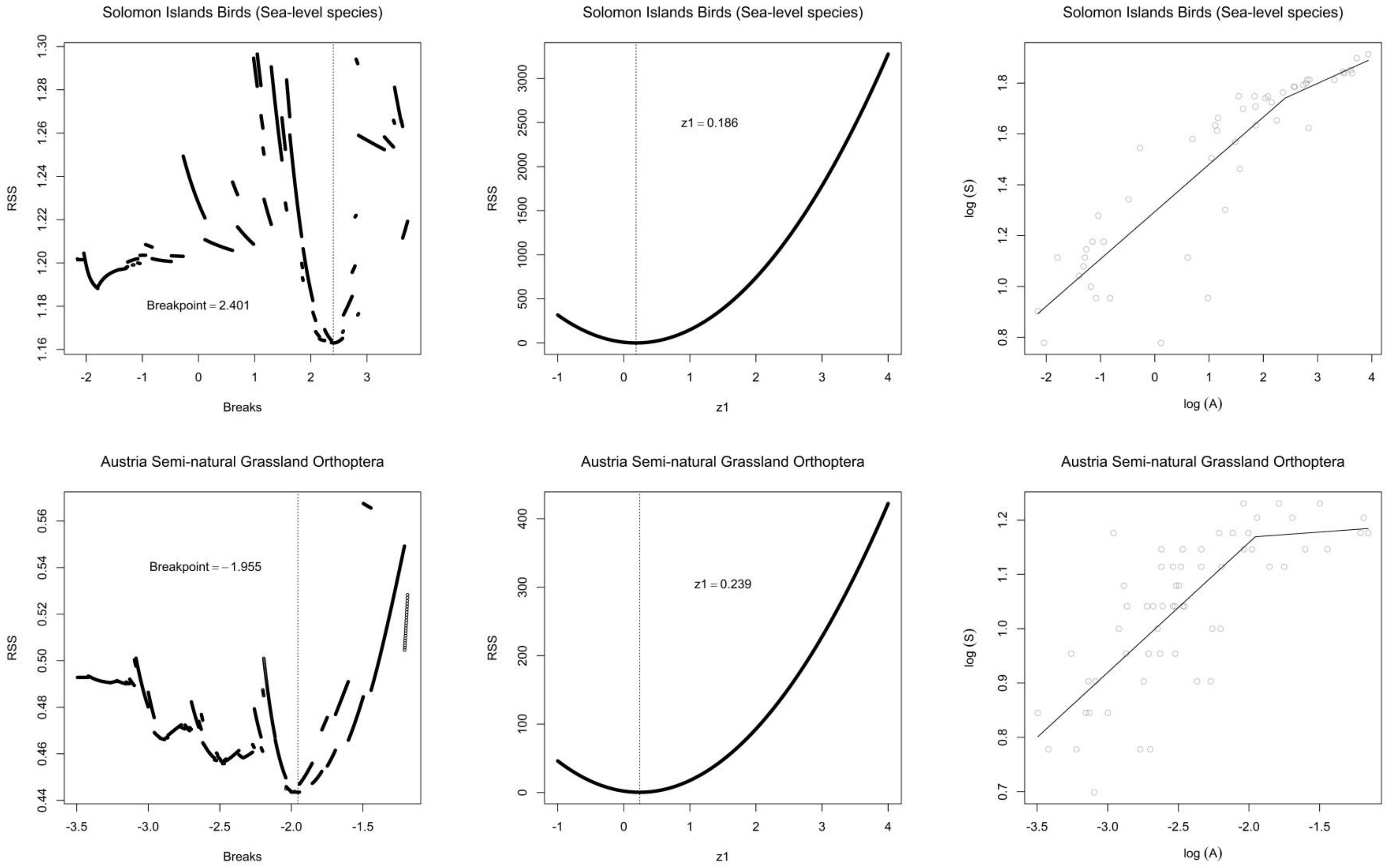

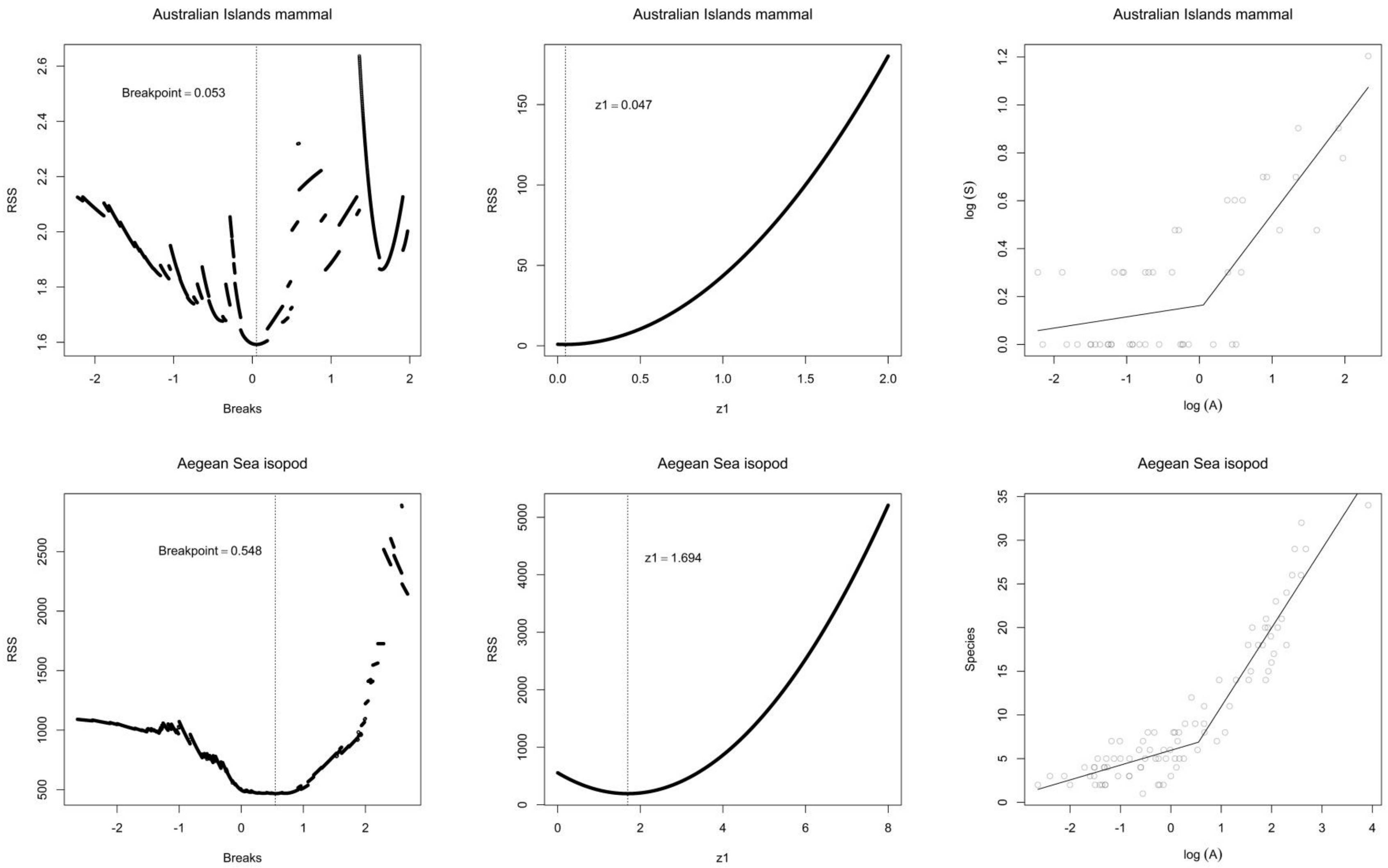
Australian Islands mammal
Breakpoint = 0.053
RSS
Breaks
Australian Islands mammal
z1 = 0.047
RSS
z1
Australian Islands mammal
log (S)
log (A)
Aegean Sea isopod
Breakpoint = 0.548
RSS
Breaks
Aegean Sea isopod
z1 = 1.694
RSS
z1
Aegean Sea isopod
Species
log (A)

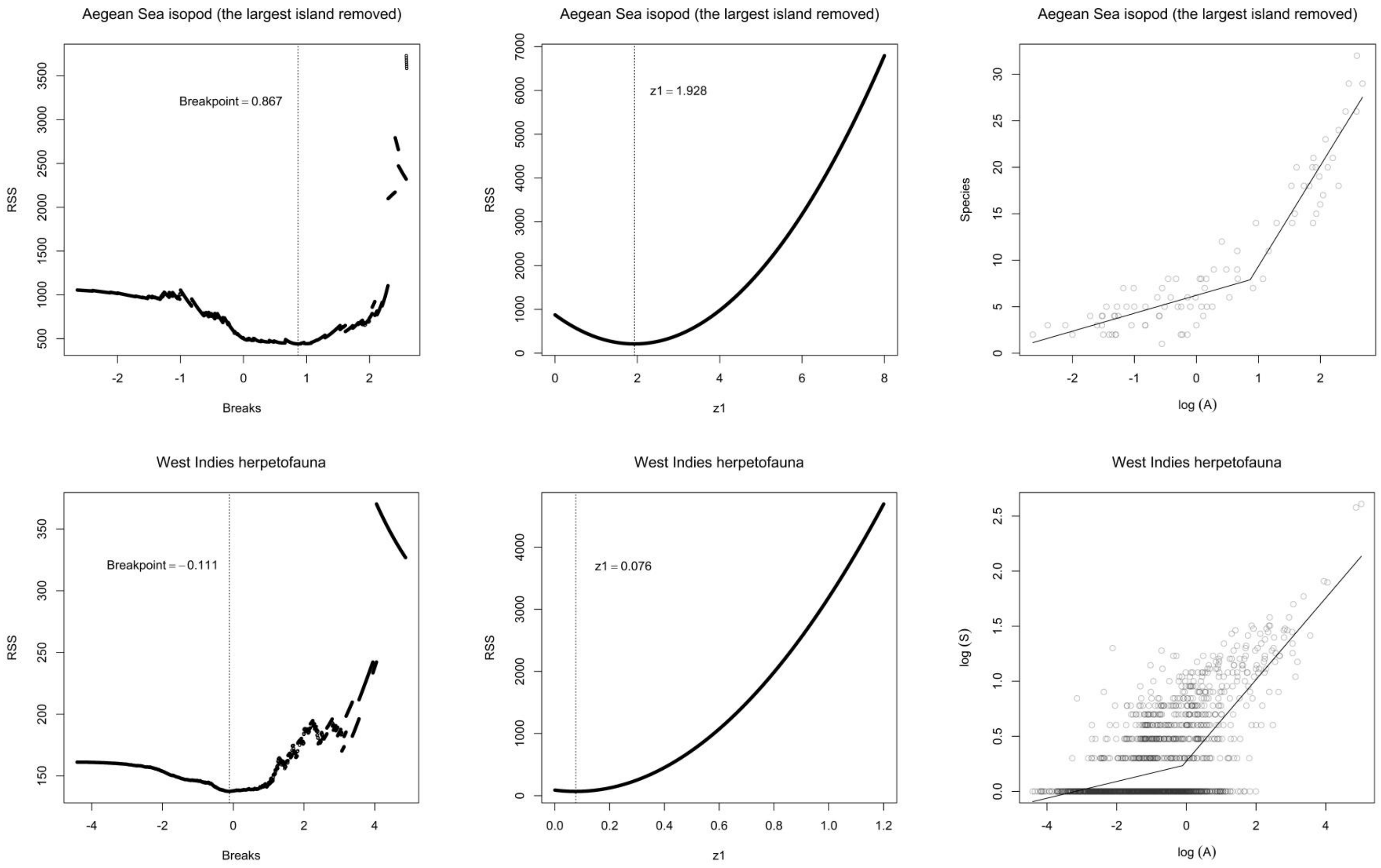
Aegean Sea isopod (the largest island removed)
Breakpoint = 0.867
RSS
Breaks
Aegean Sea isopod (the largest island removed)
z1 = 1.928
RSS
z1
Aegean Sea isopod (the largest island removed)
Species
log (A)
West Indies herpetofauna
Breakpoint = −0.111
RSS
Breaks
West Indies herpetofauna
z1 = 0.076
RSS
z1
West Indies herpetofauna
log (S)
log (A)

Figure S4. Results and the iterative processes used in Model (4) regression analyses for six sample datasets in accordance with Fig. 2. The breakpoint (T_1) that returns a minimal residual sum of squares (RSS) was chosen.

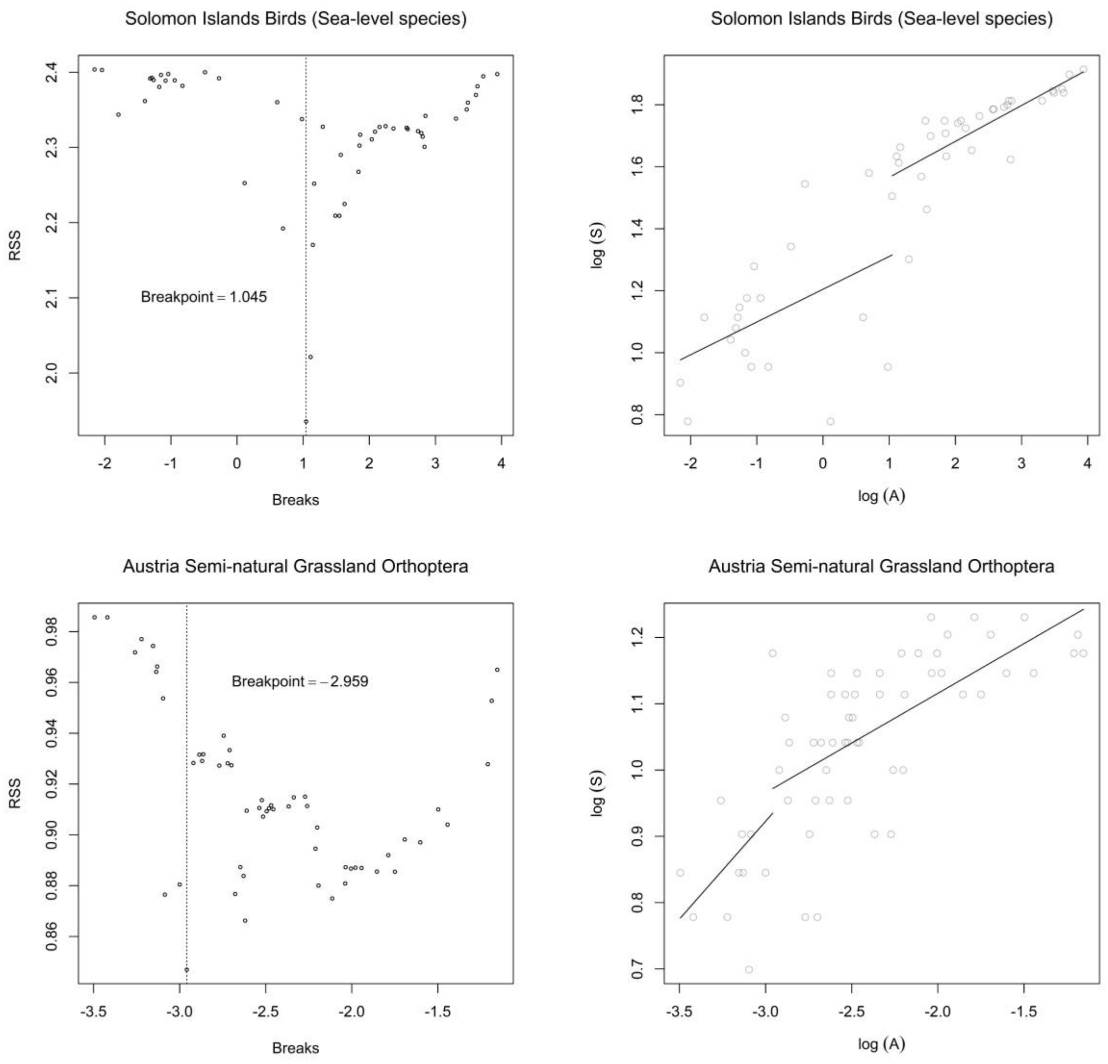

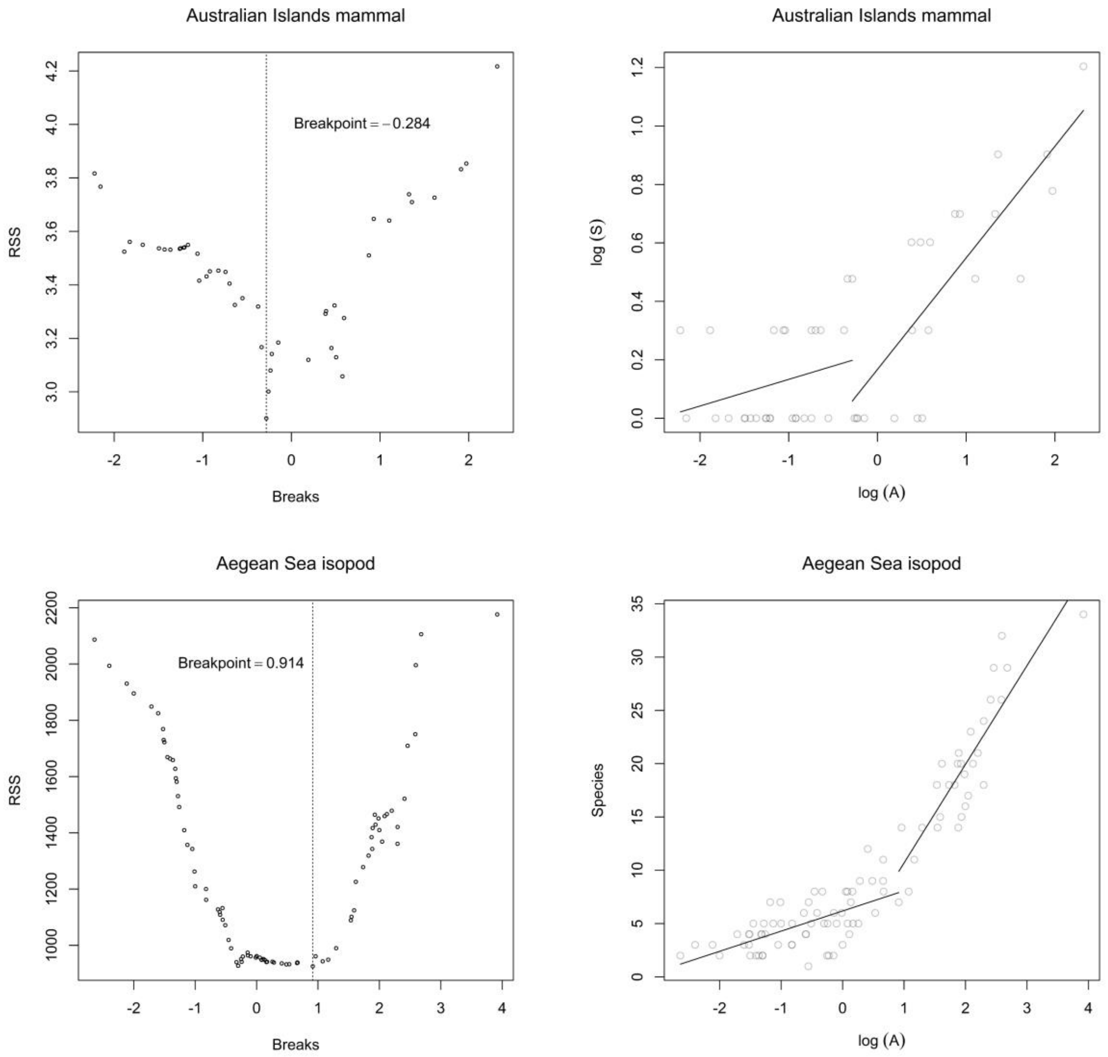
Australian Islands mammal
Breakpoint = −0.284
RSS
Breaks
Australian Islands mammal
log (S)
log (A)
Aegean Sea isopod
Breakpoint = 0.914
RSS
Breaks
Aegean Sea isopod
Species
log (A)

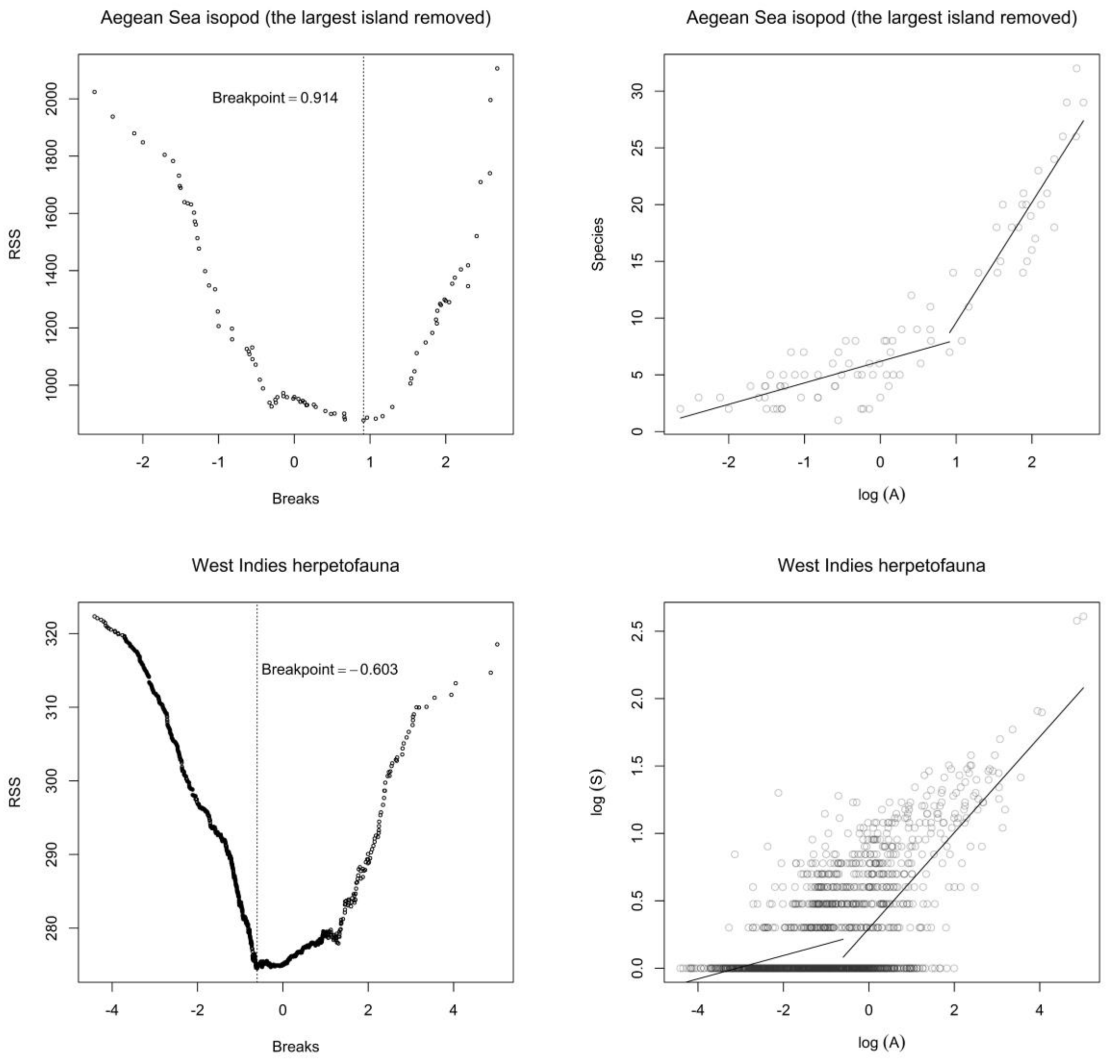

Aegean Sea isopod (the largest island removed)
Breakpoint = 0.914
RSS
Breaks
Aegean Sea isopod (the largest island removed)
Species
log (A)
West Indies herpetofauna
Breakpoint = −0.603
RSS
Breaks
West Indies herpetofauna
log (S)
log (A)

Figure S5. Results and the double iterative processes used in Model (5) regression analyses for six sample datasets in accordance with Fig. 2. The breakpoint (T_1) that returns a minimal residual sum of squares (RSS) was chosen. After T_1 was determined, we run iterative processes of c_1 and z_1 again to look for the c_1 and z_1 that produced the minimum RSS value.

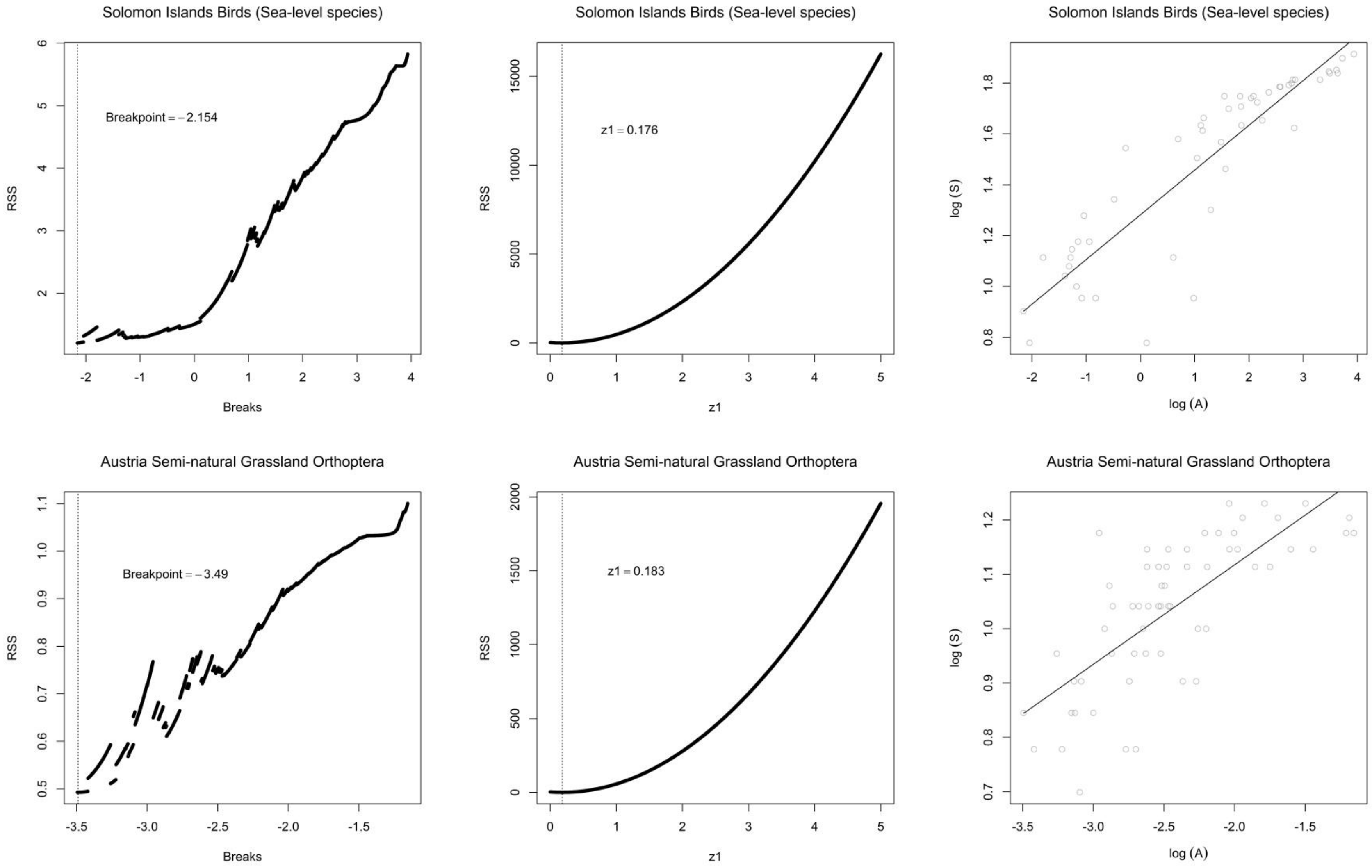

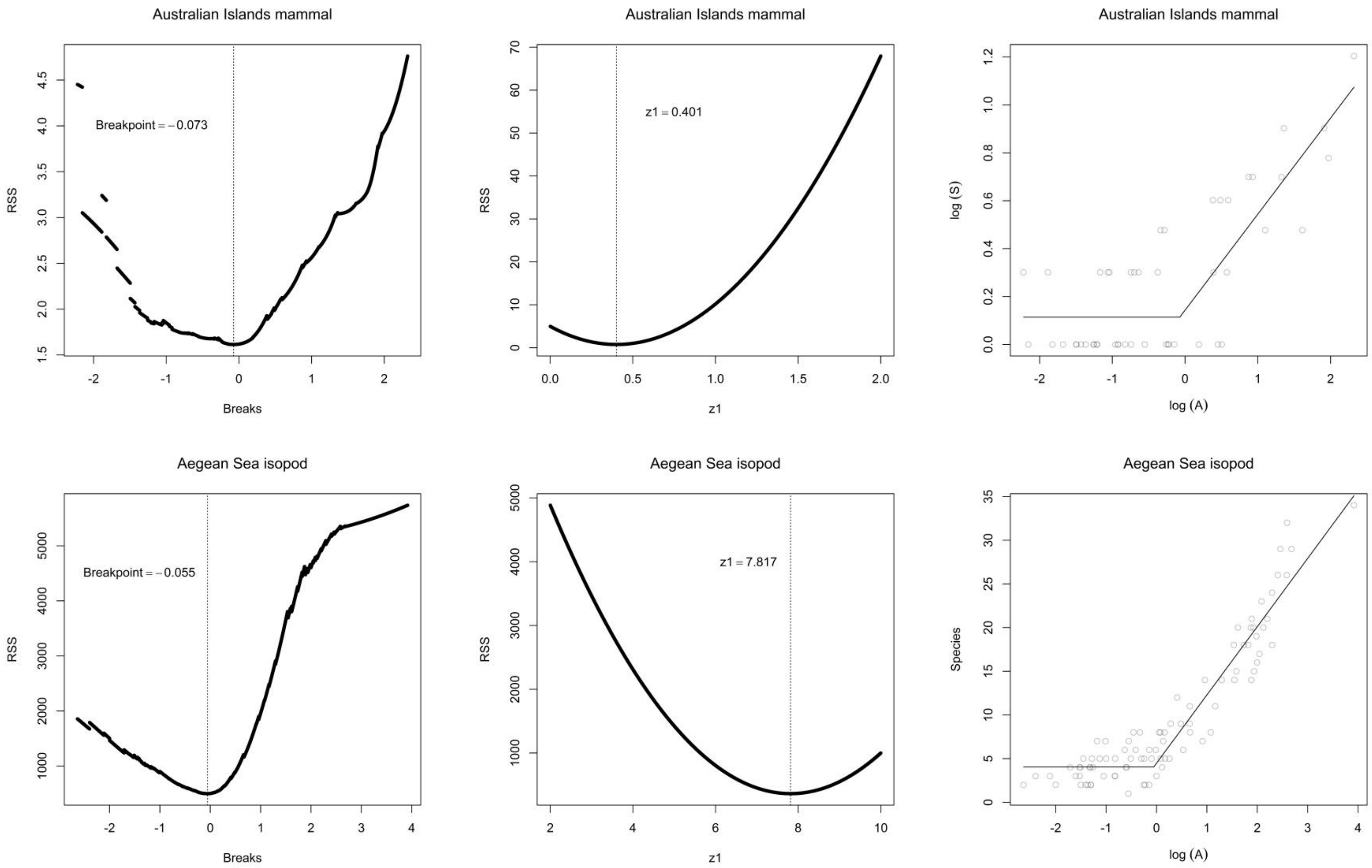
Australian Islands mammal
RSS
Breaks
Breakpoint = −0.073
Australian Islands mammal
RSS
z1
z1 = 0.401
Australian Islands mammal
log (S)
log (A)
Aegean Sea isopod
RSS
Breaks
Breakpoint = −0.055
Aegean Sea isopod
RSS
z1
z1 = 7.817
Aegean Sea isopod
Species
log (A)

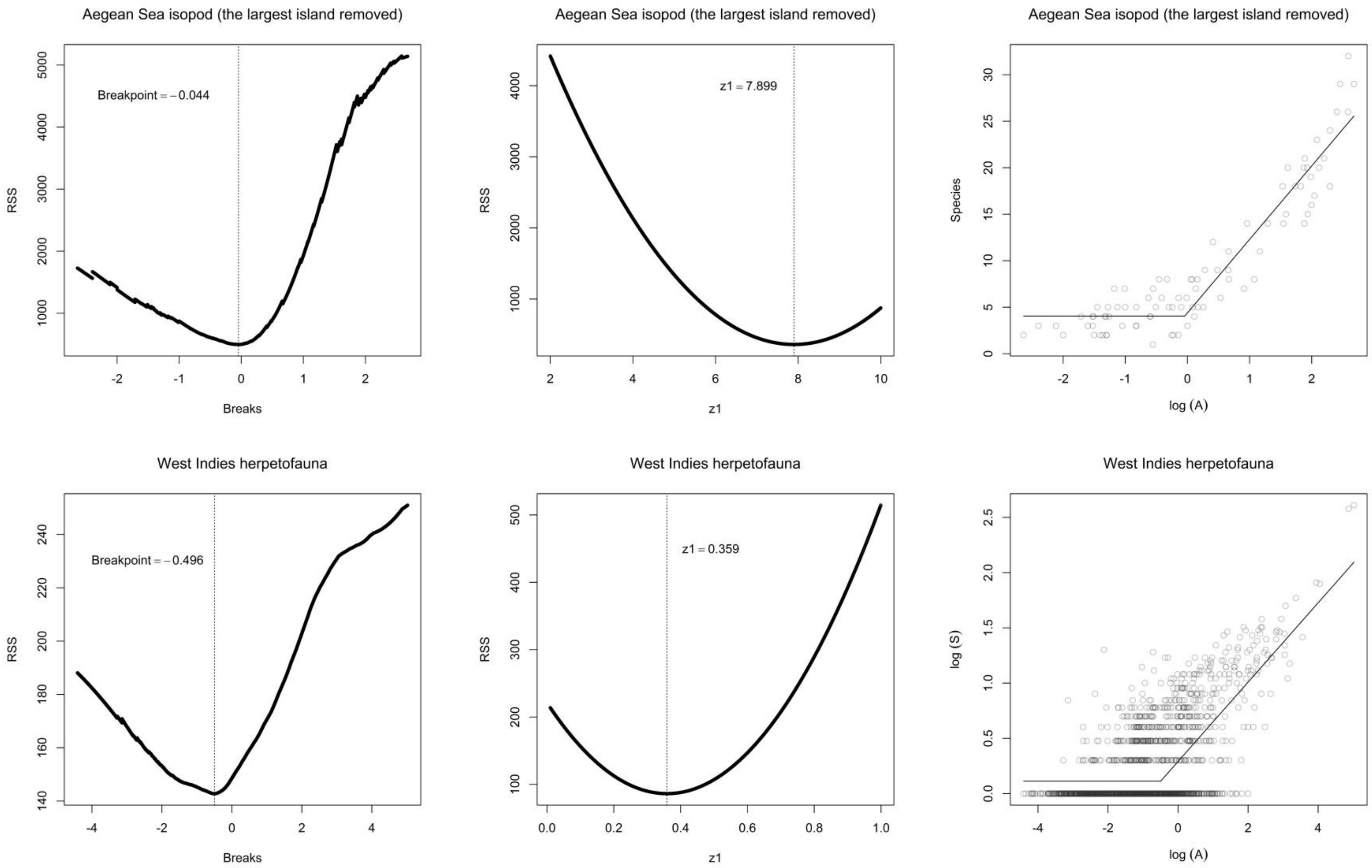
Aegean Sea isopod (the largest island removed)
Breakpoint = −0.044
RSS
Breaks
Aegean Sea isopod (the largest island removed)
z1 = 7.899
RSS
z1
Aegean Sea isopod (the largest island removed)
Species
log (A)
West Indies herpetofauna
Breakpoint = −0.496
RSS
Breaks
West Indies herpetofauna
z1 = 0.359
RSS
z1
West Indies herpetofauna
log (S)
log (A)

Figure S6. Results and the iterative processes used in Model (6) regression analyses for six sample datasets in accordance with Fig. 2. The breakpoint (T_1) that returns a minimal residual sum of squares (RSS) was chosen.

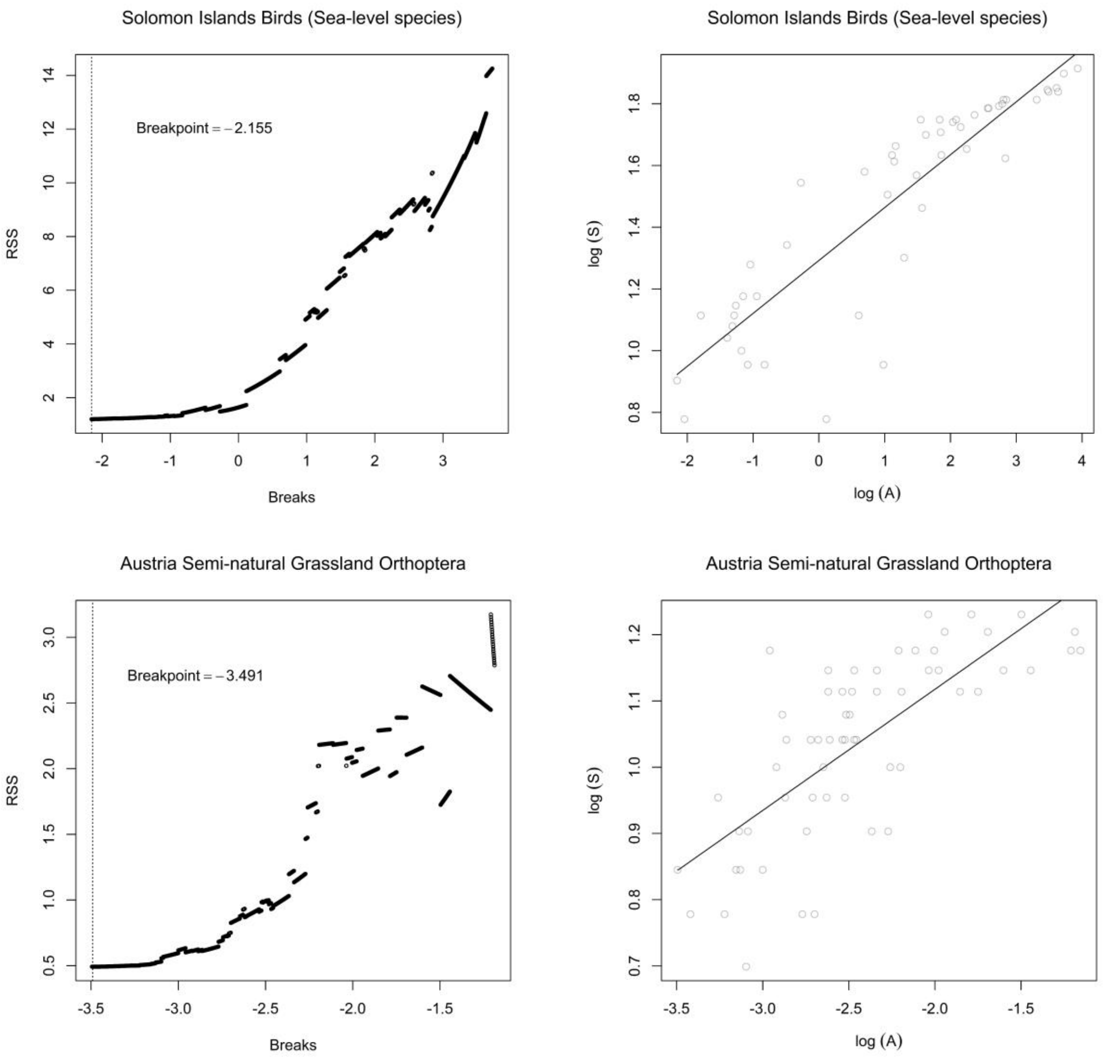

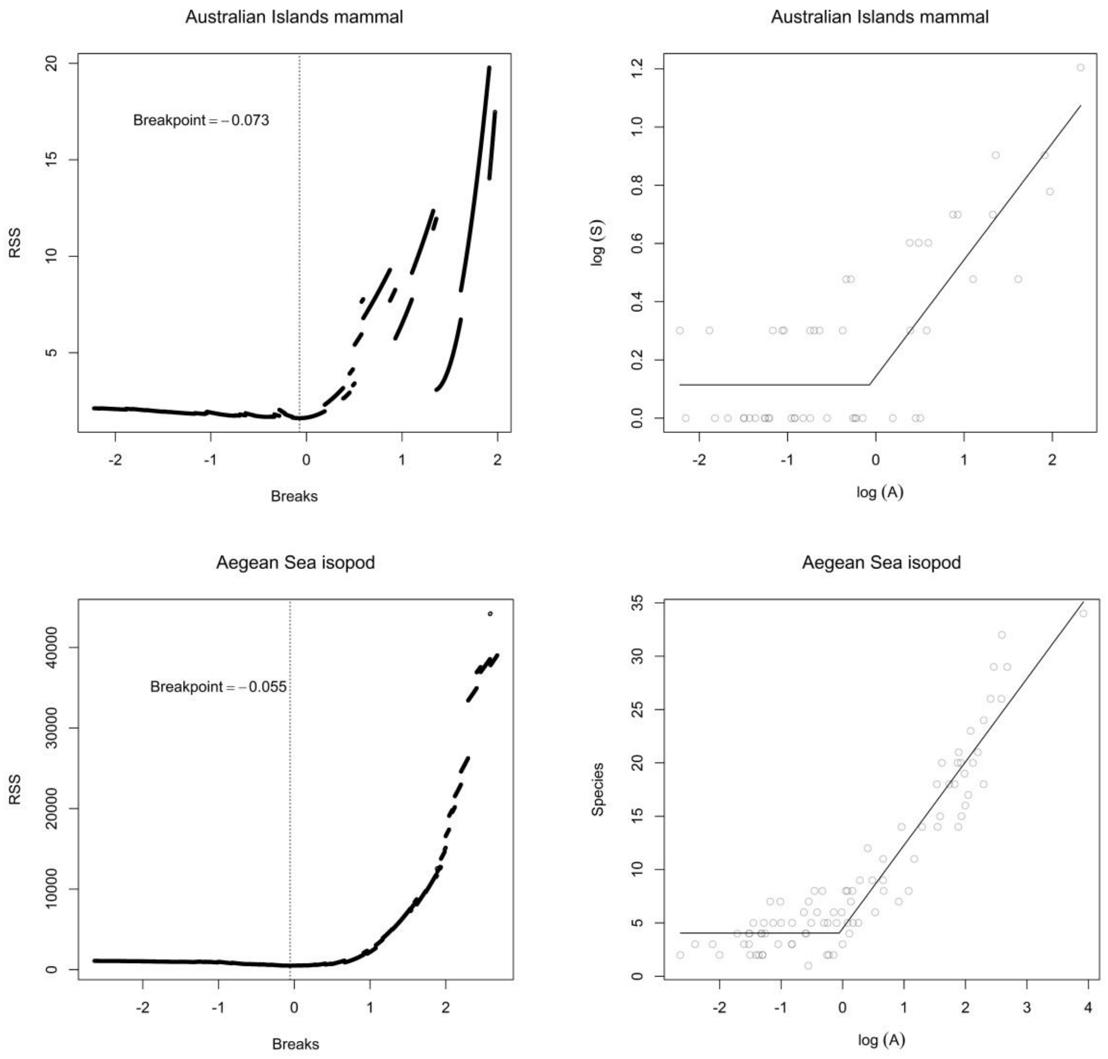
Australian Islands mammal
RSS
Breaks
Breakpoint = −0.073
Australian Islands mammal
log (S)
log (A)
Aegean Sea isopod
RSS
Breaks
Breakpoint = −0.055
Aegean Sea isopod
Species
log (A)

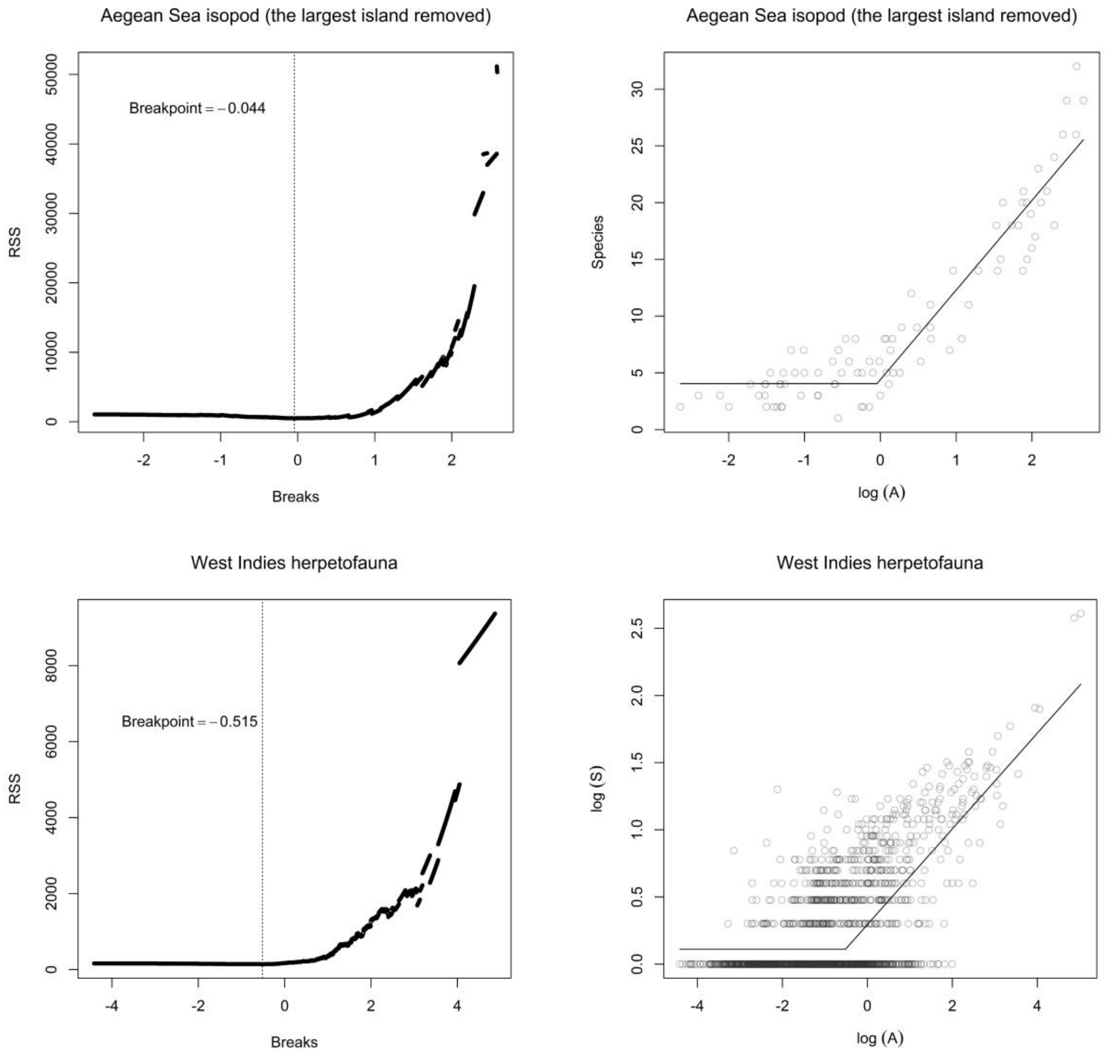
Aegean Sea isopod (the largest island removed)
Breakpoint = −0.044
RSS
Breaks
Aegean Sea isopod (the largest island removed)
Species
log (A)
West Indies herpetofauna
Breakpoint = −0.515
RSS
Breaks
West Indies herpetofauna
log (S)
log (A)

Figure S7. Results and the iterative processes used in Model (7) regression analyses for six sample datasets in accordance with Fig. 2. The breakpoint (T_1) that returns a minimal residual sum of squares (RSS) was chosen. After T_1 was determined, we run iterative process of c_1 again to look for the c_1 that produced the minimum RSS value.

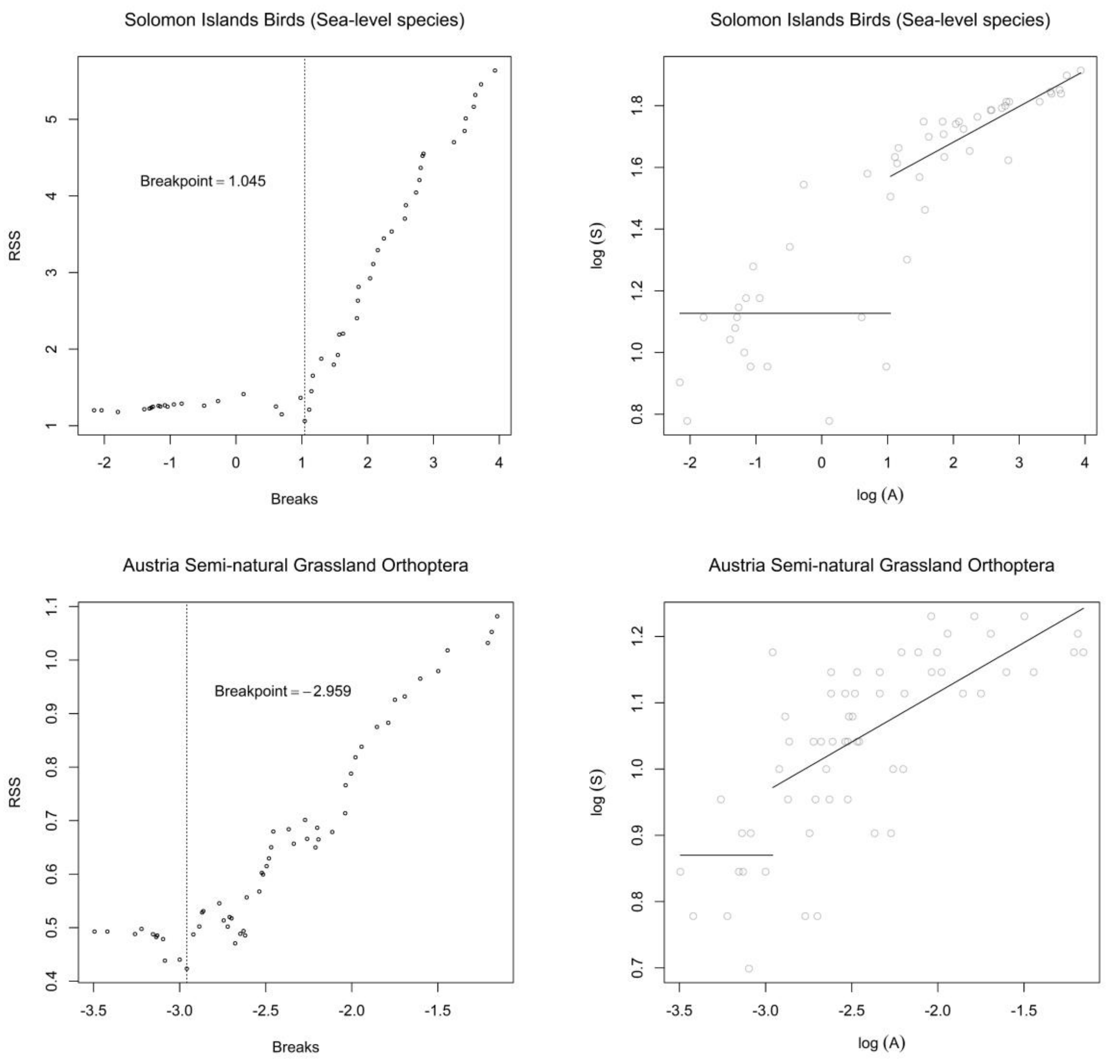

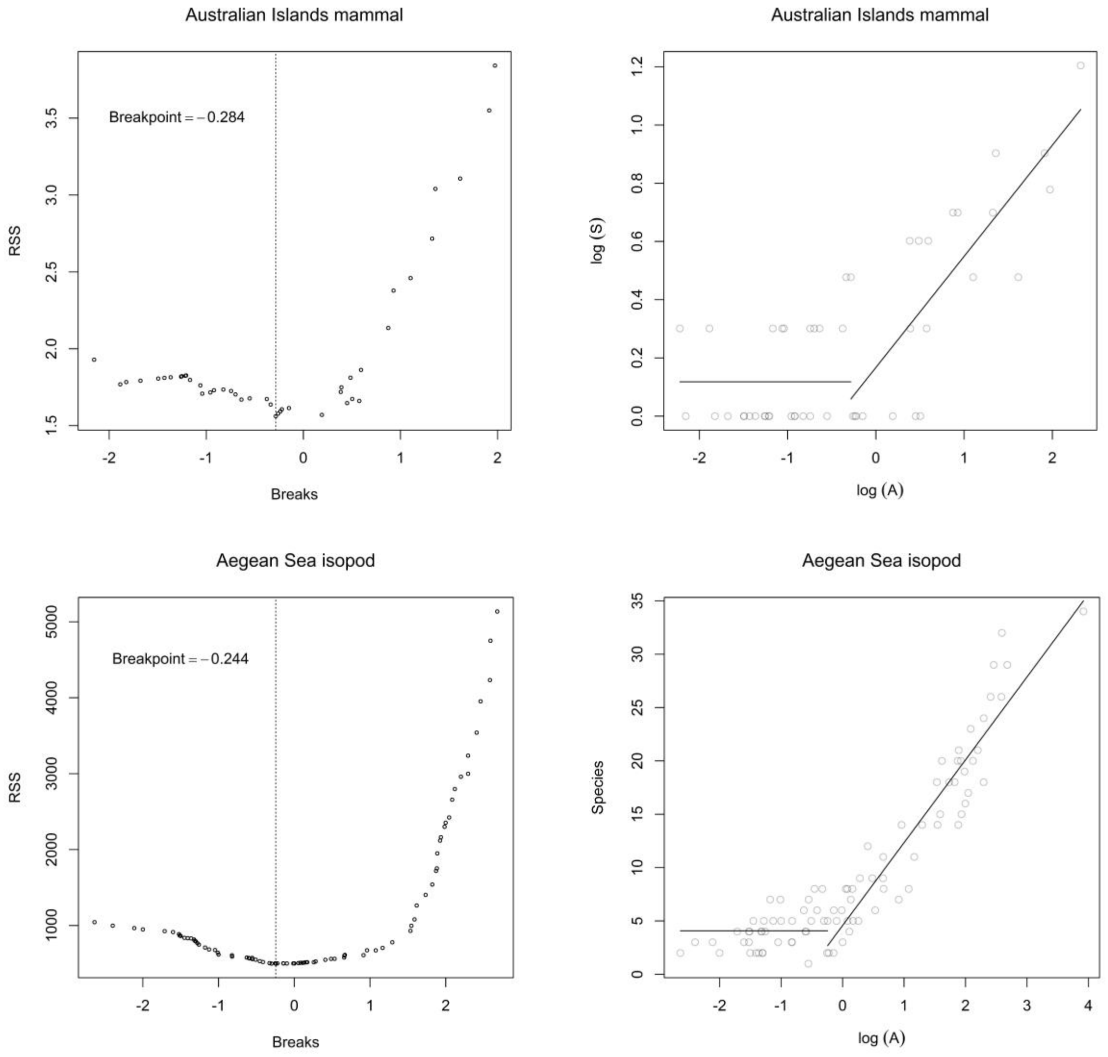
Australian Islands mammal
Breakpoint = −0.284
RSS
Breaks
Australian Islands mammal
log (S)
log (A)
Aegean Sea isopod
Breakpoint = −0.244
RSS
Breaks
Aegean Sea isopod
Species
log (A)

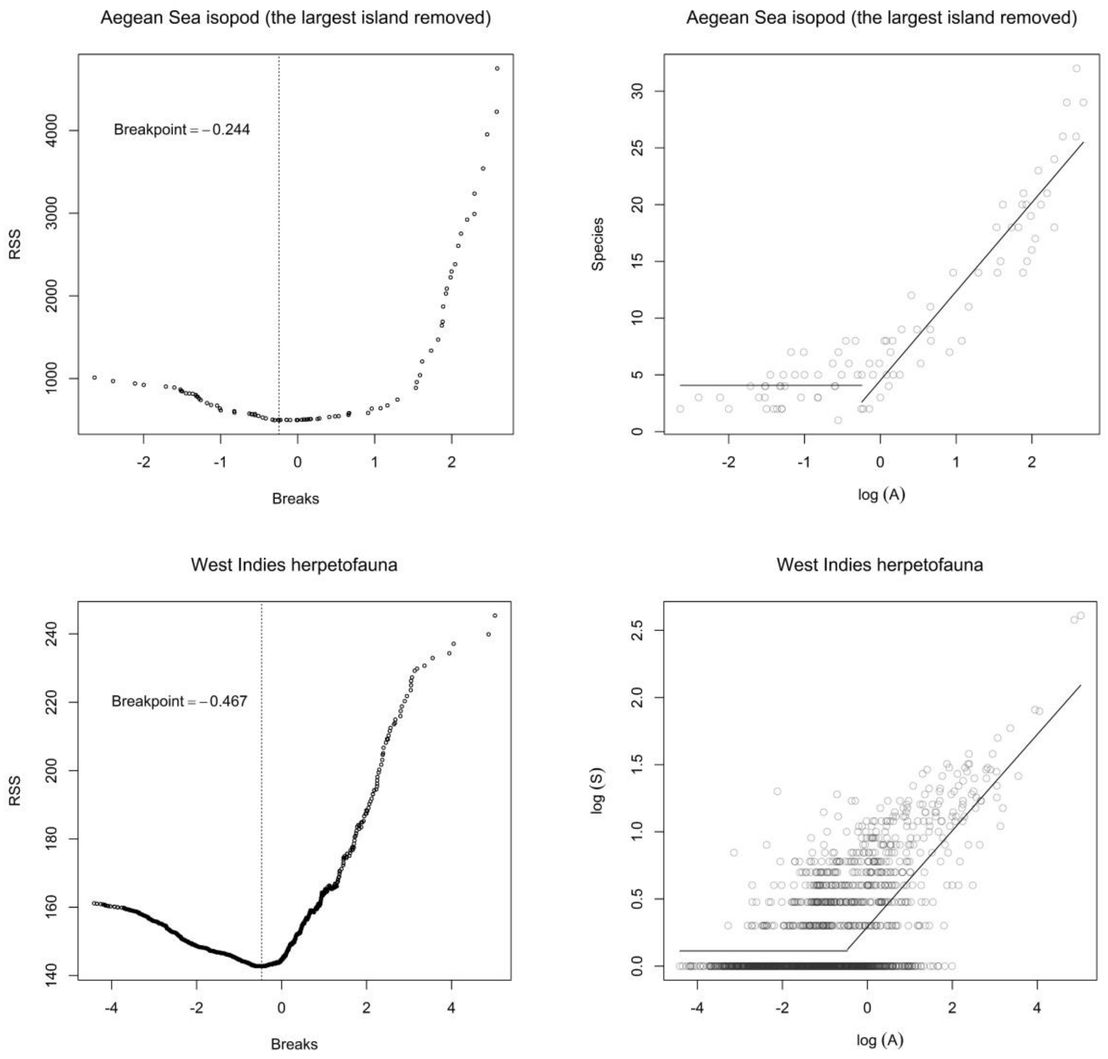
Aegean Sea isopod (the largest island removed)
Breakpoint = −0.244
RSS
Breaks
Aegean Sea isopod (the largest island removed)
Species
log (A)
West Indies herpetofauna
Breakpoint = −0.467
RSS
Breaks
West Indies herpetofauna
log (S)
log (A)

Figure S8. Results and the iterative processes used in Model (8) regression analyses for six sample datasets in accordance with Fig. 2. The breakpoint (T_1) that returns a minimal residual sum of squares (RSS) was chosen.

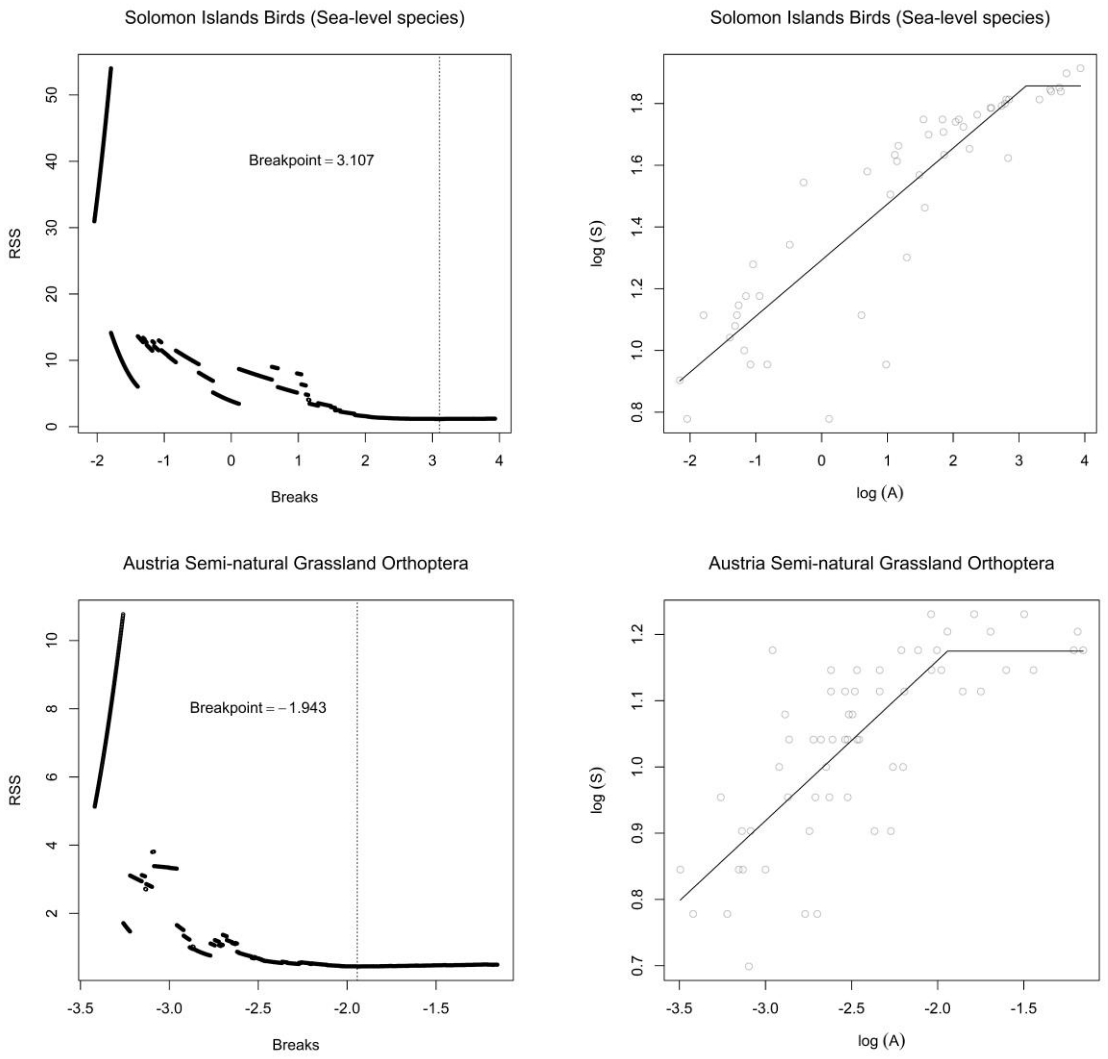

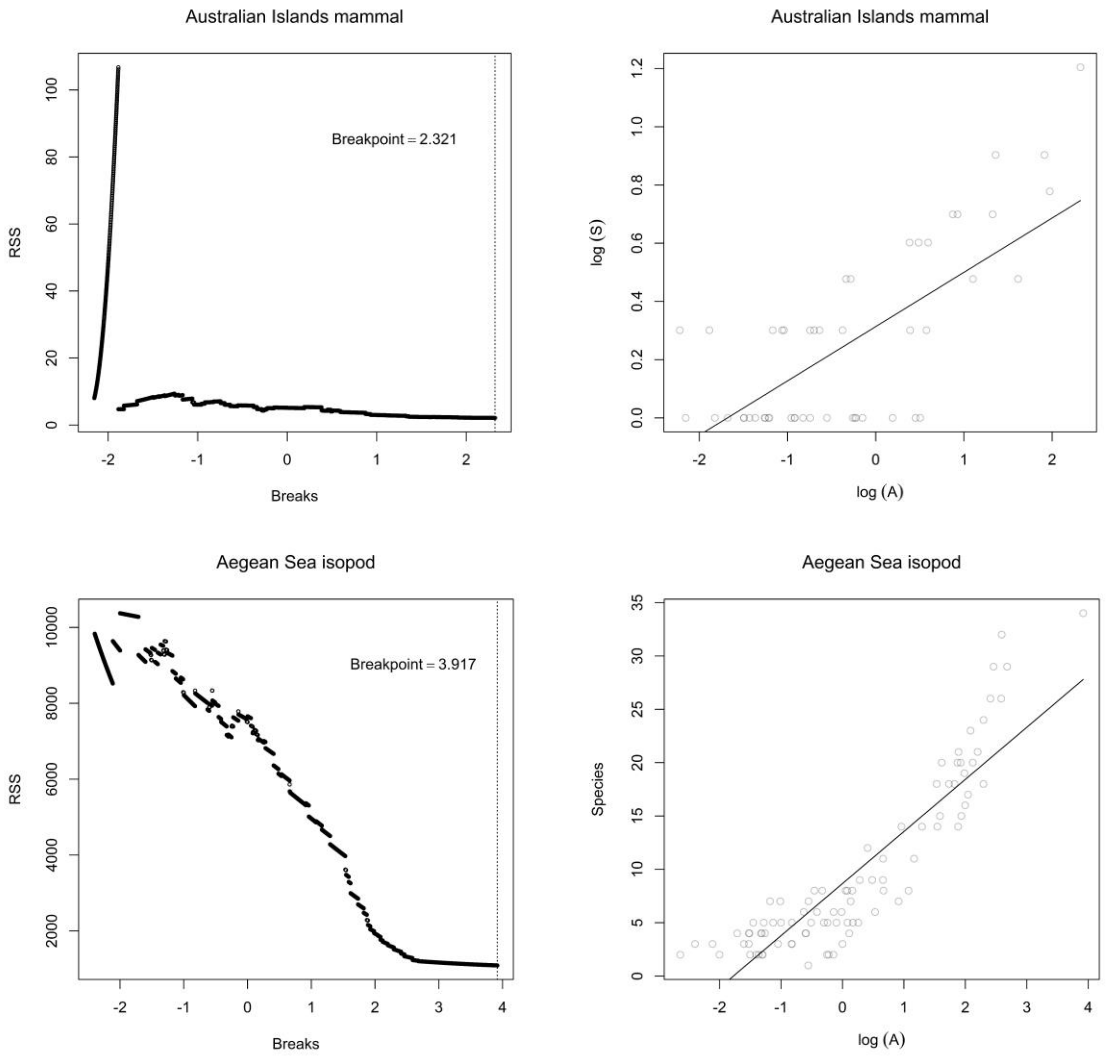
Australian Islands mammal
Breakpoint = 2.321
RSS
Breaks
Australian Islands mammal
log (S)
log (A)
Aegean Sea isopod
Breakpoint = 3.917
RSS
Breaks
Aegean Sea isopod
Species
log (A)

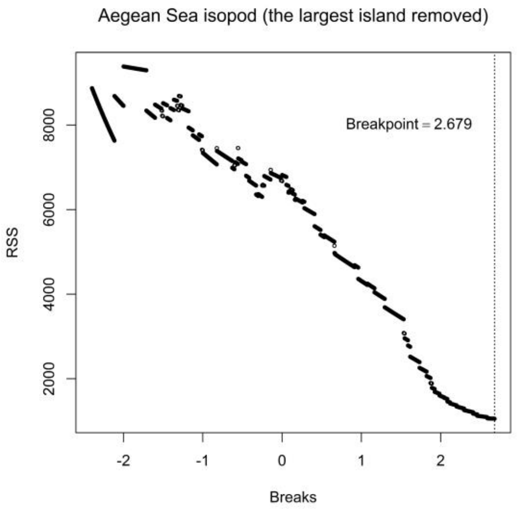
Aegean Sea isopod (the largest island removed)
Breakpoint = 2.679
RSS
2000
4000
6000
8000
-2
-1
0
1
2
Breaks

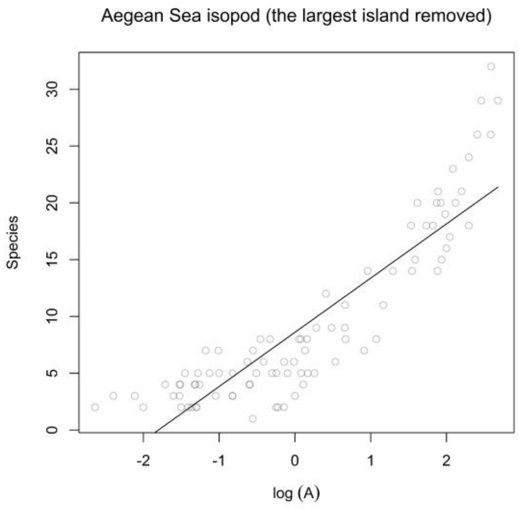
Aegean Sea isopod (the largest island removed)
Species
0
5
10
15
20
25
30
-2
-1
0
1
2
log (A)

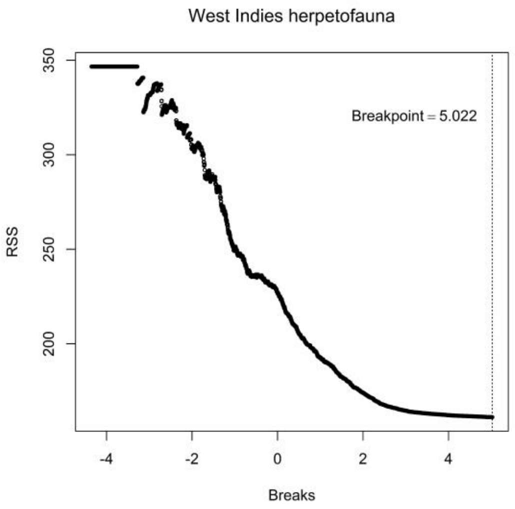
West Indies herpetofauna
Breakpoint = 5.022
RSS
200
250
300
350
-4
-2
0
2
4
Breaks

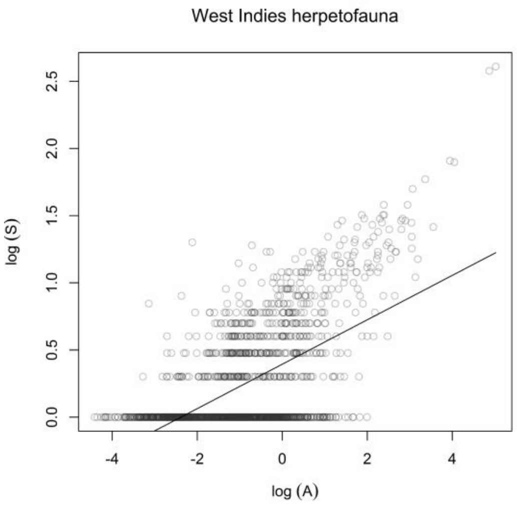
West Indies herpetofauna
log (S)
0.0
0.5
1.0
1.5
2.0
2.5
-4
-2
0
2
4
log (A)

Figure S9. Results and the double iterative processes used in Model (9) regression analyses for six sample datasets in accordance with Fig. 2. The breakpoint (T_1) that returns a minimal residual sum of squares (RSS) was chosen. After T_1 was determined, we run iterative processes of c_1 and z_1 again to look for the c_1 and z_1 that produced the minimum RSS value.

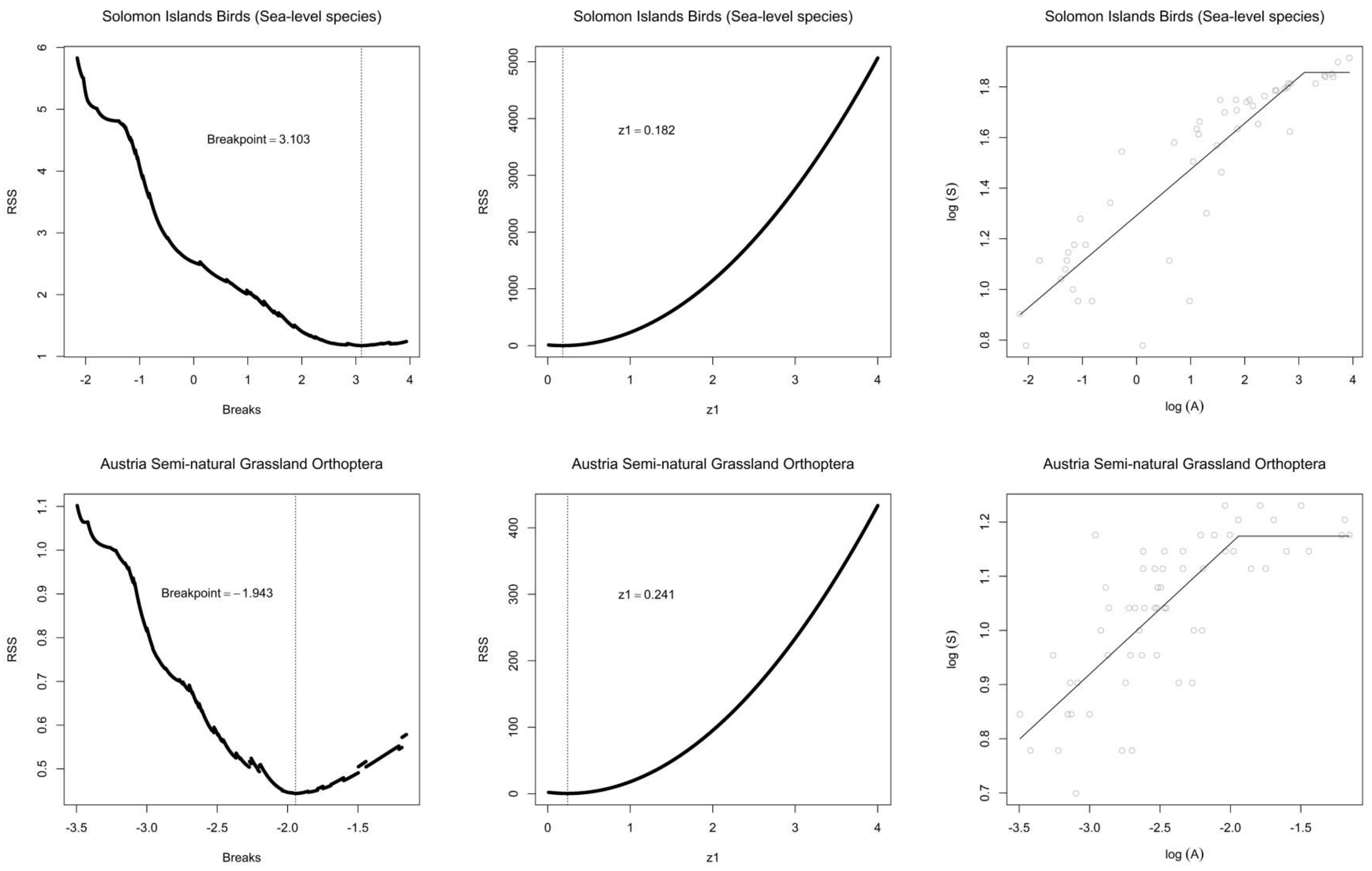

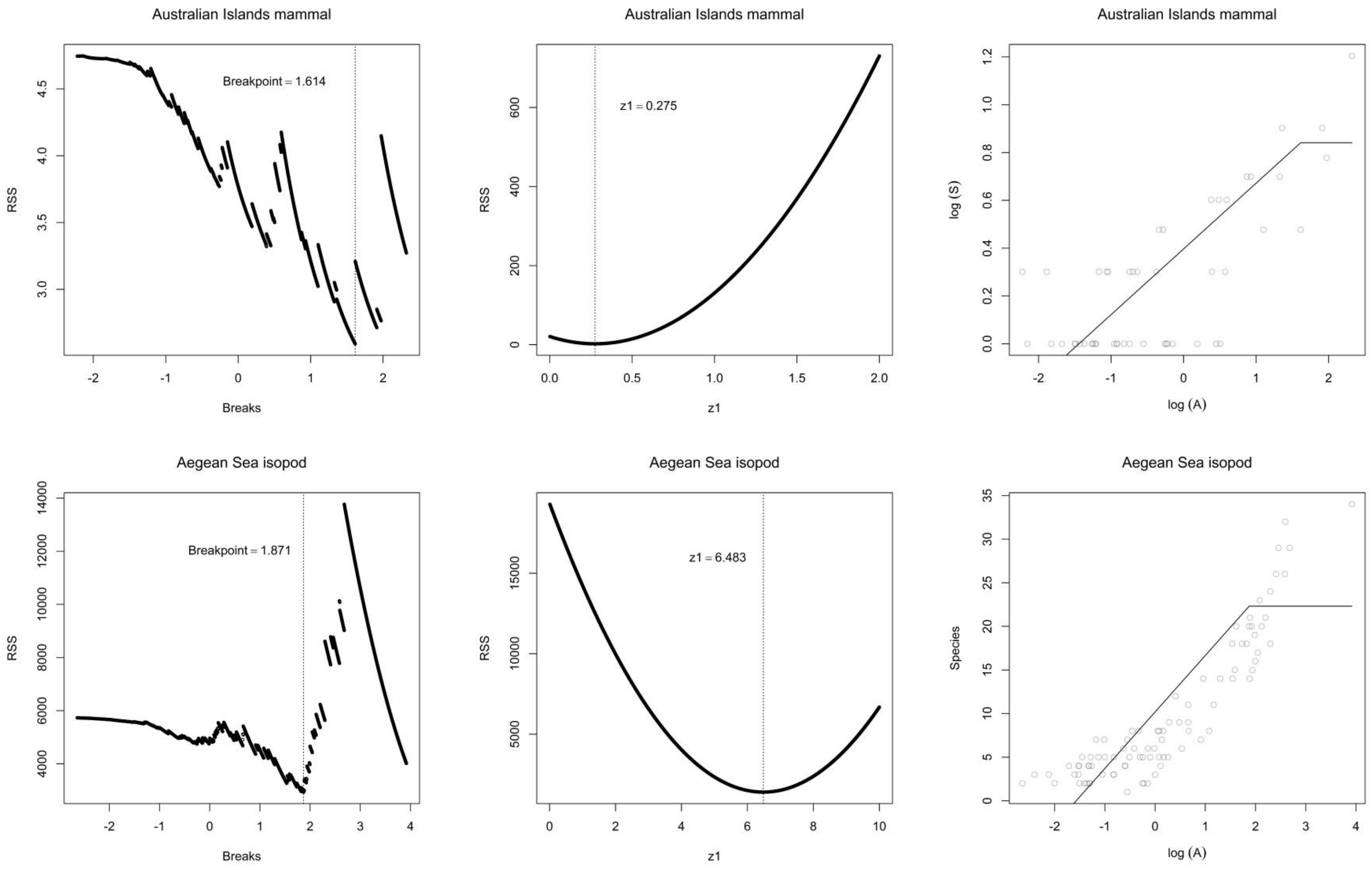
Australian Islands mammal
Breakpoint = 1.614
RSS
Breaks
Australian Islands mammal
z1 = 0.275
RSS
z1
Australian Islands mammal
log (S)
log (A)
Aegean Sea isopod
Breakpoint = 1.871
RSS
Breaks
Aegean Sea isopod
z1 = 6.483
RSS
z1
Aegean Sea isopod
Species
log (A)

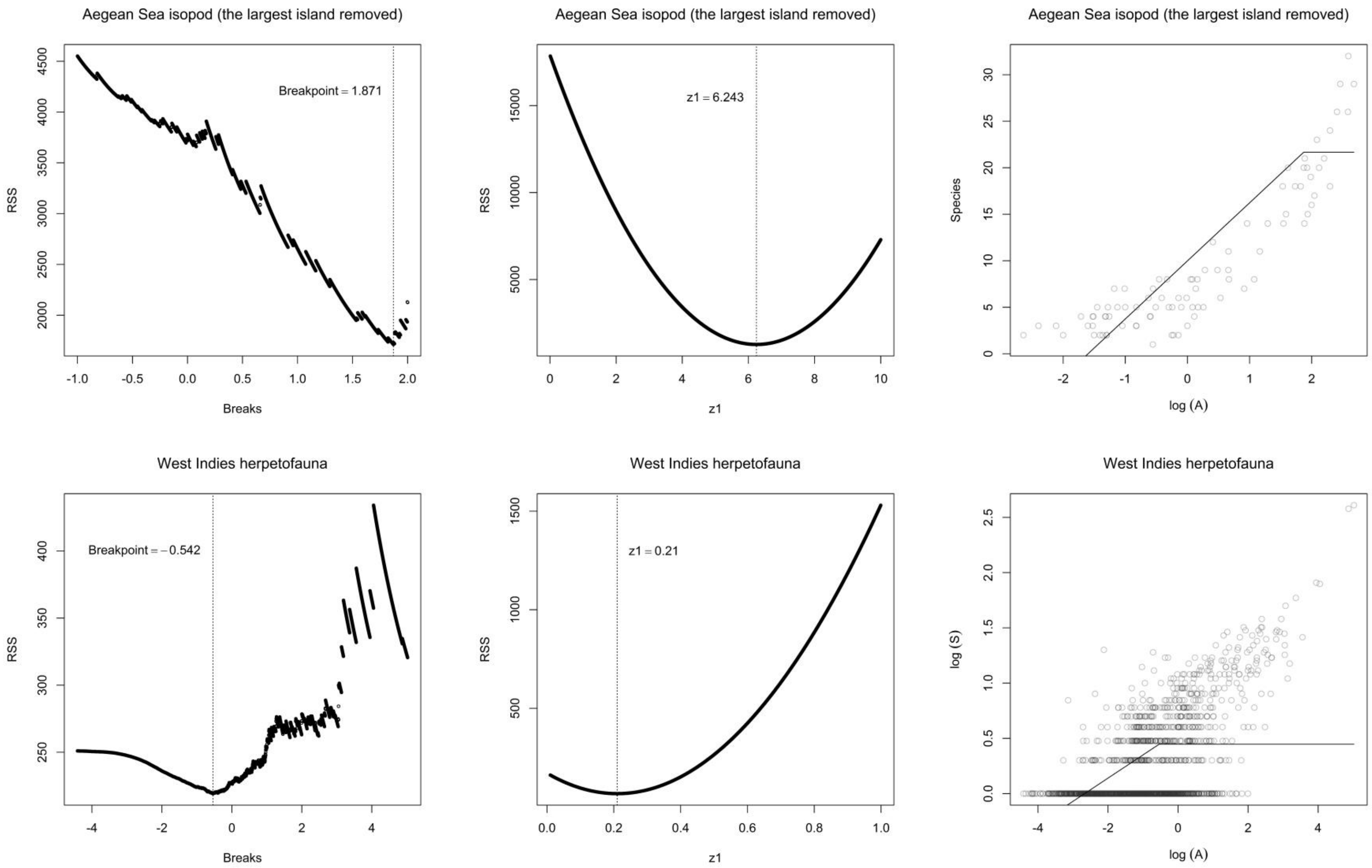
Aegean Sea isopod (the largest island removed)
Breakpoint = 1.871
RSS
Breaks
Aegean Sea isopod (the largest island removed)
z1 = 6.243
RSS
z1
Aegean Sea isopod (the largest island removed)
Species
log (A)
West Indies herpetofauna
Breakpoint = −0.542
RSS
Breaks
West Indies herpetofauna
z1 = 0.21
RSS
z1
West Indies herpetofauna
log (S)
log (A)

Figure S10. Results and the iterative processes used in Model (10) regression analyses for six sample datasets in accordance with Fig. 2. The breakpoint (T_1) that returns a minimal residual sum of squares (RSS) was chosen. After T_1 was determined, we run iterative process of c_2 again to look for the c_2 that produced the minimum RSS value.

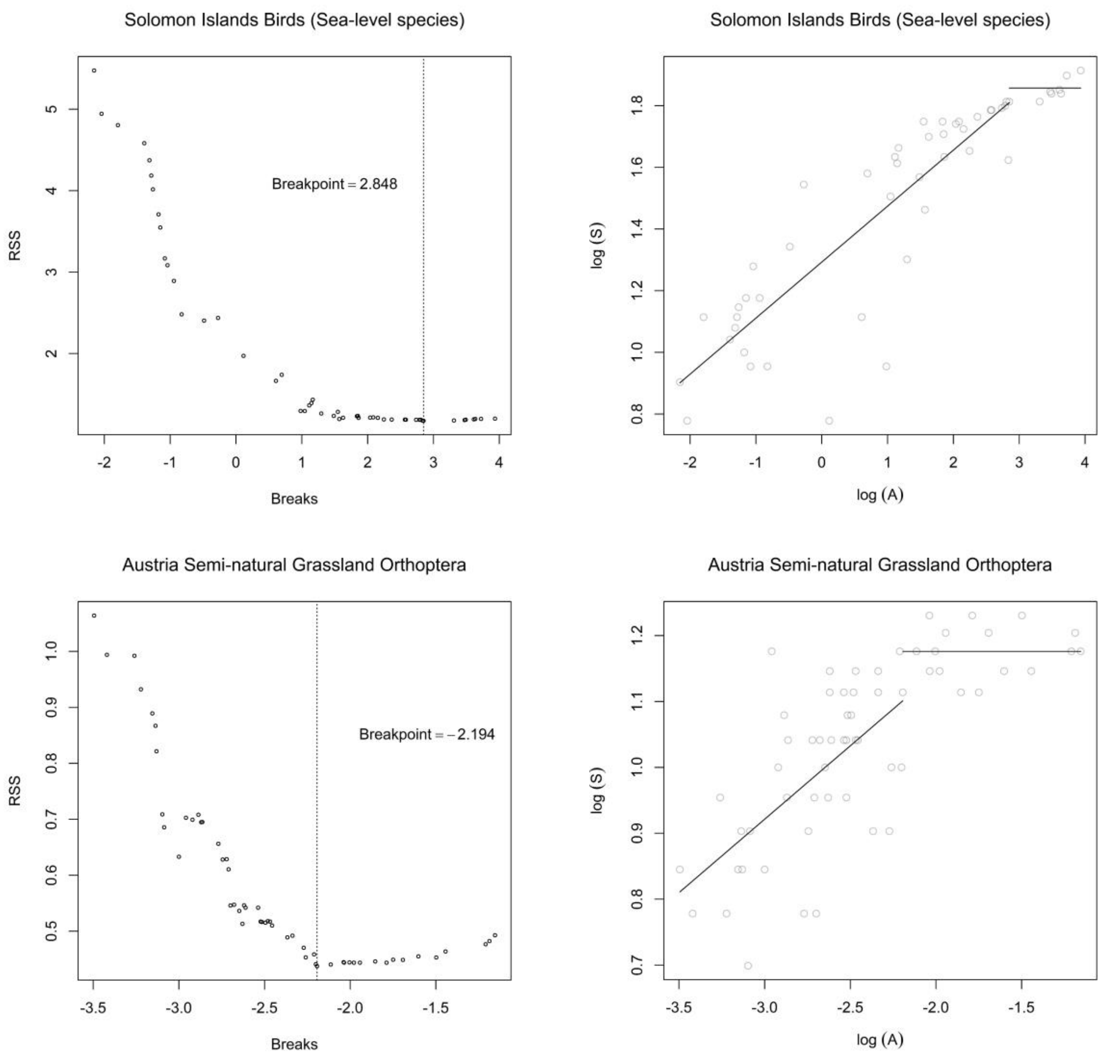

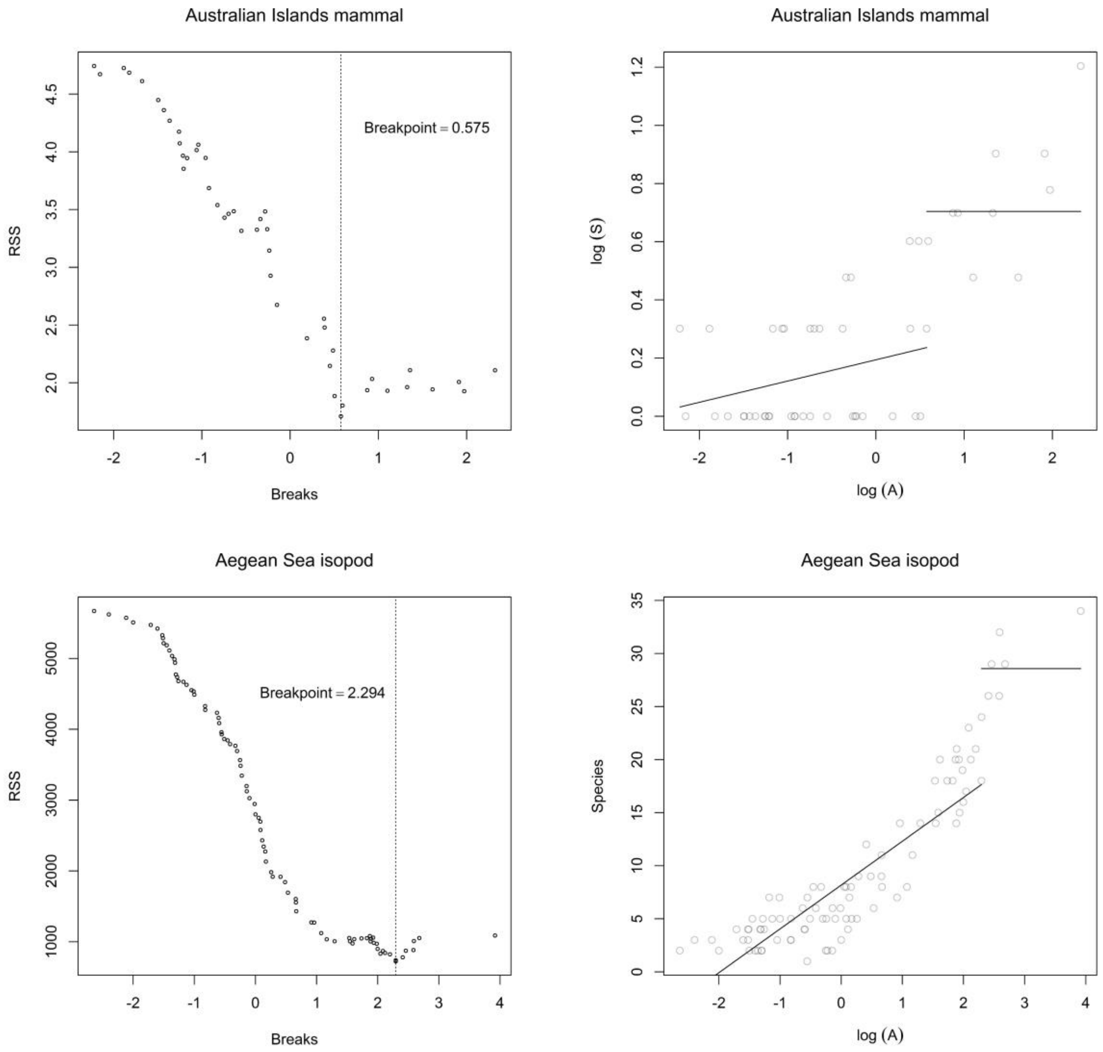
Australian Islands mammal
Breakpoint = 0.575
RSS
Breaks
Australian Islands mammal
log (S)
log (A)
Aegean Sea isopod
Breakpoint = 2.294
RSS
Breaks
Aegean Sea isopod
Species
log (A)

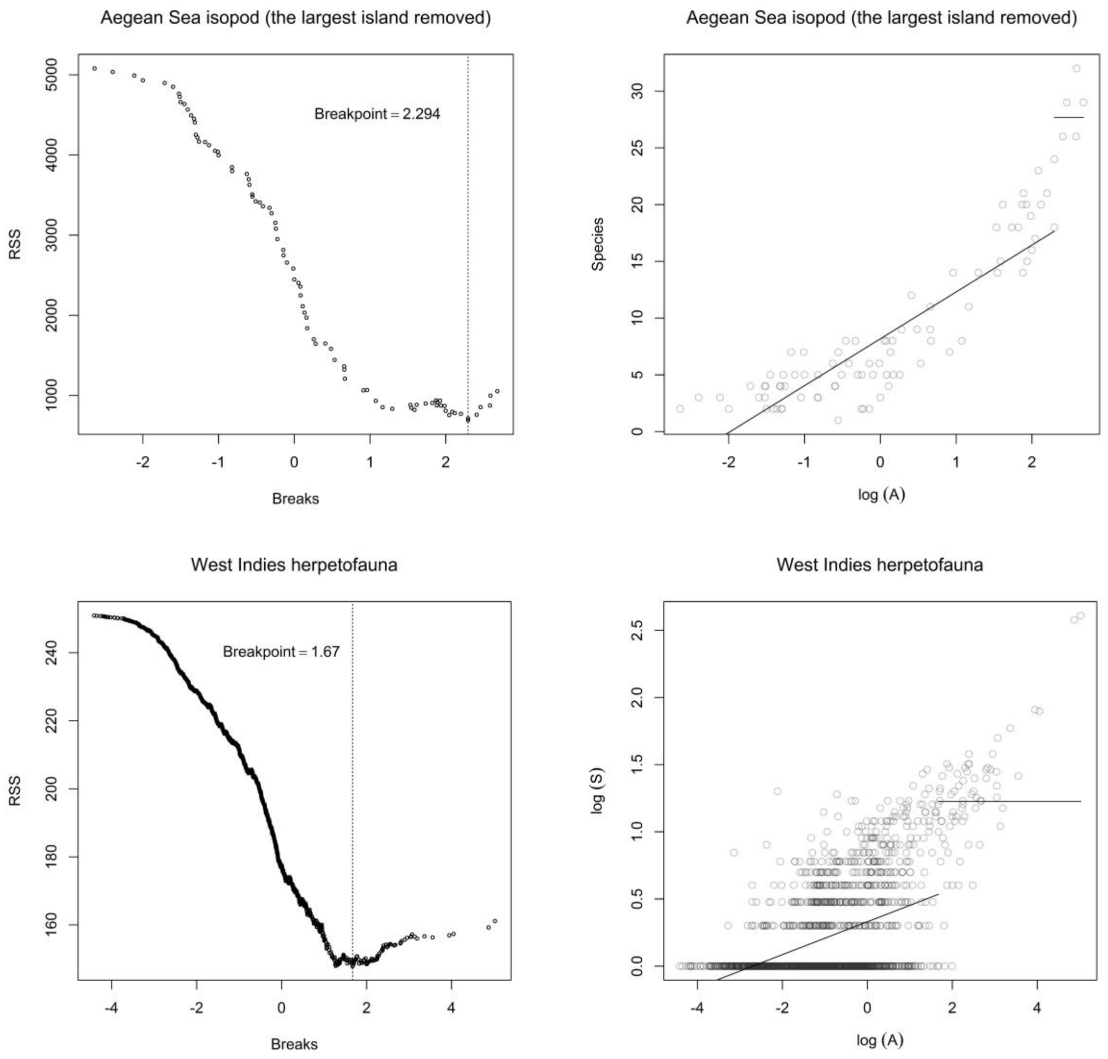
Aegean Sea isopod (the largest island removed)
Breakpoint = 2.294
RSS
Breaks
Aegean Sea isopod (the largest island removed)
Species
log (A)
West Indies herpetofauna
Breakpoint = 1.67
RSS
Breaks
West Indies herpetofauna
log (S)
log (A)

Figure S11. Results and the double iterative processes used in Model (11) regression analyses for six sample datasets in accordance with Fig. 2. The second breakpoint (T_2) was obtained prior to the first one (T_1). After T_2 was determined, we run iterative process of T_1 again to look for the T_1 that produced the minimum RSS value.

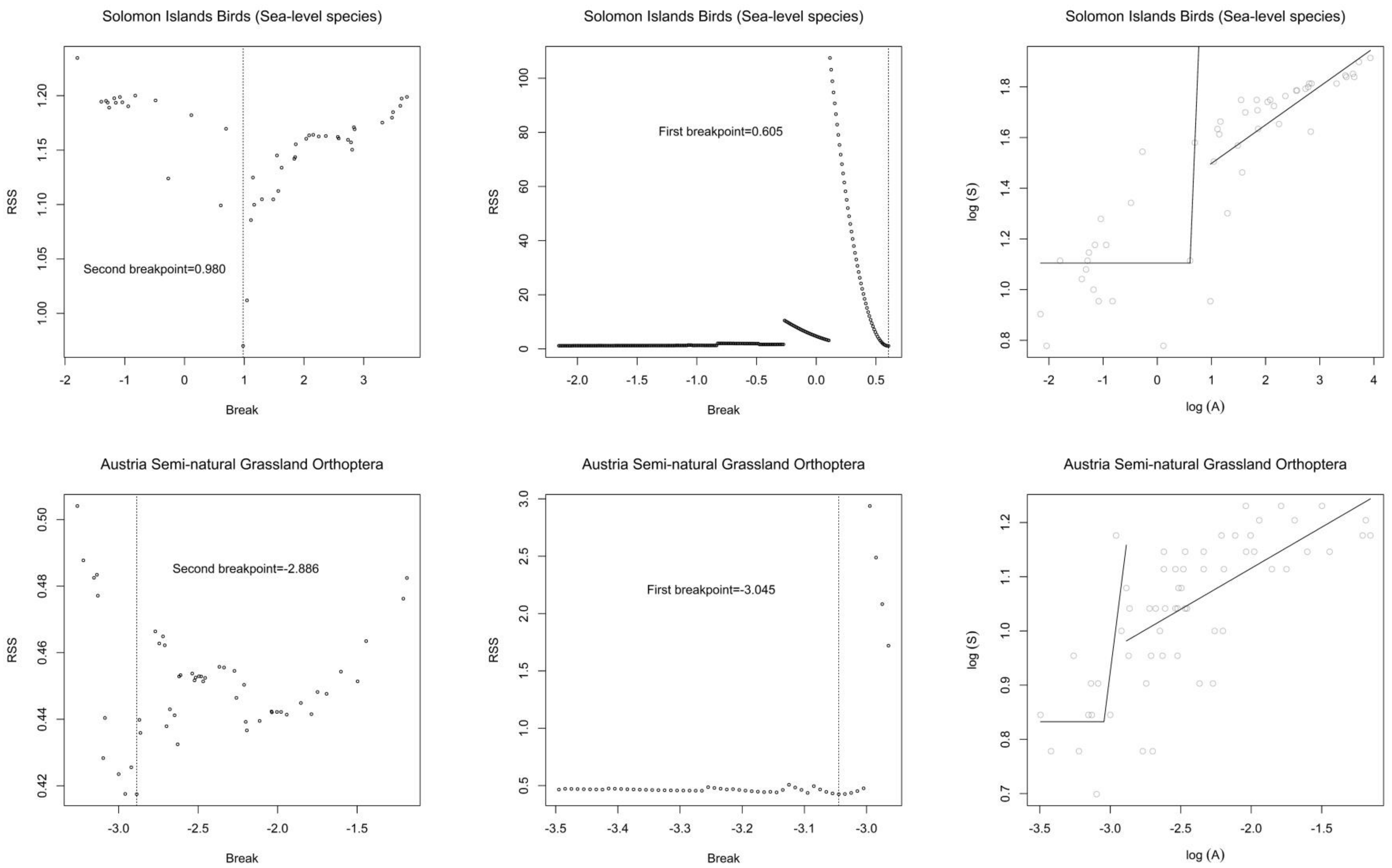

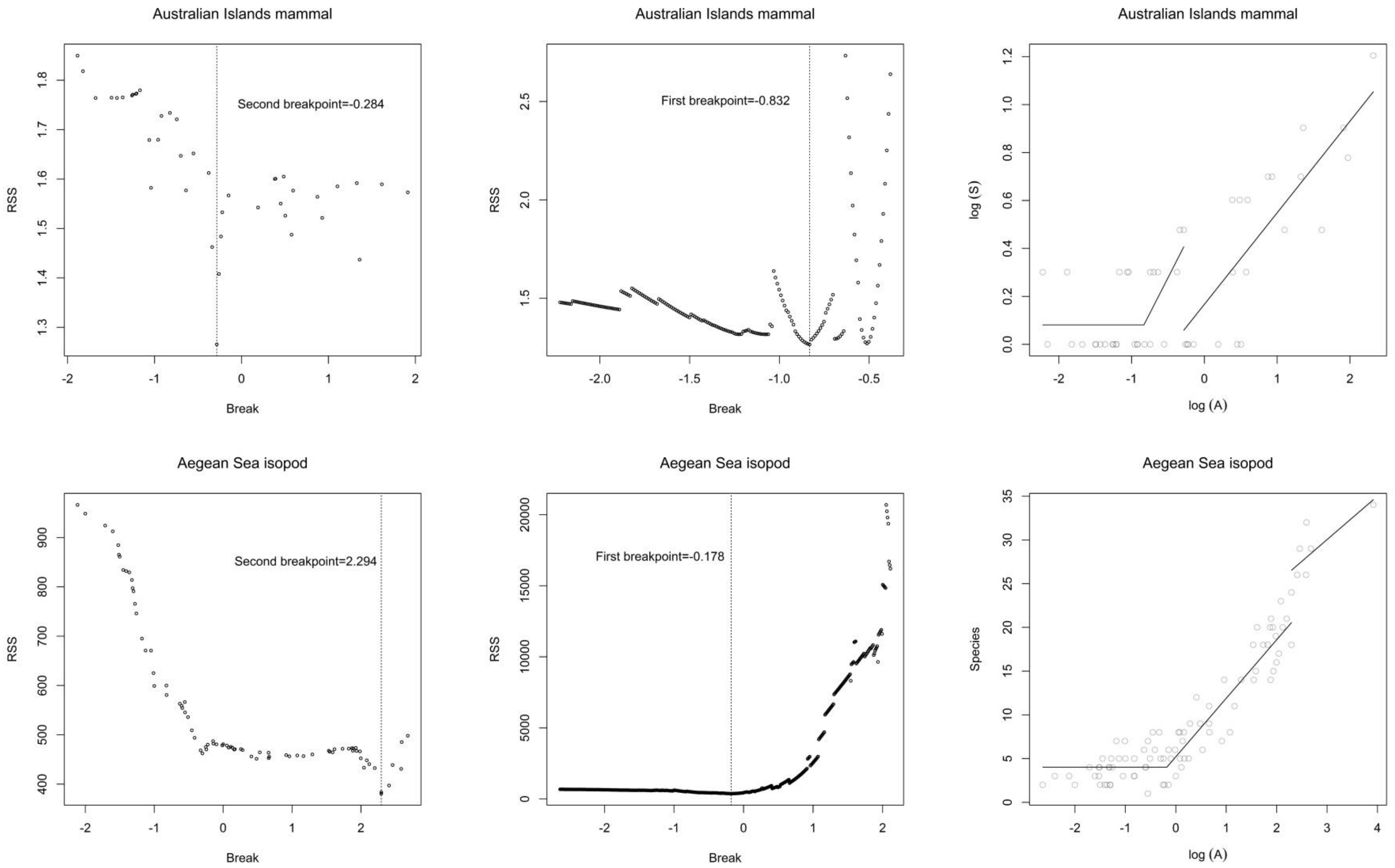
Australian Islands mammal
Second breakpoint=-0.284
RSS
Break
Australian Islands mammal
First breakpoint=-0.832
RSS
Break
Australian Islands mammal
log (S)
log (A)
Aegean Sea isopod
Second breakpoint=2.294
RSS
Break
Aegean Sea isopod
First breakpoint=-0.178
RSS
Break
Aegean Sea isopod
Species
log (A)

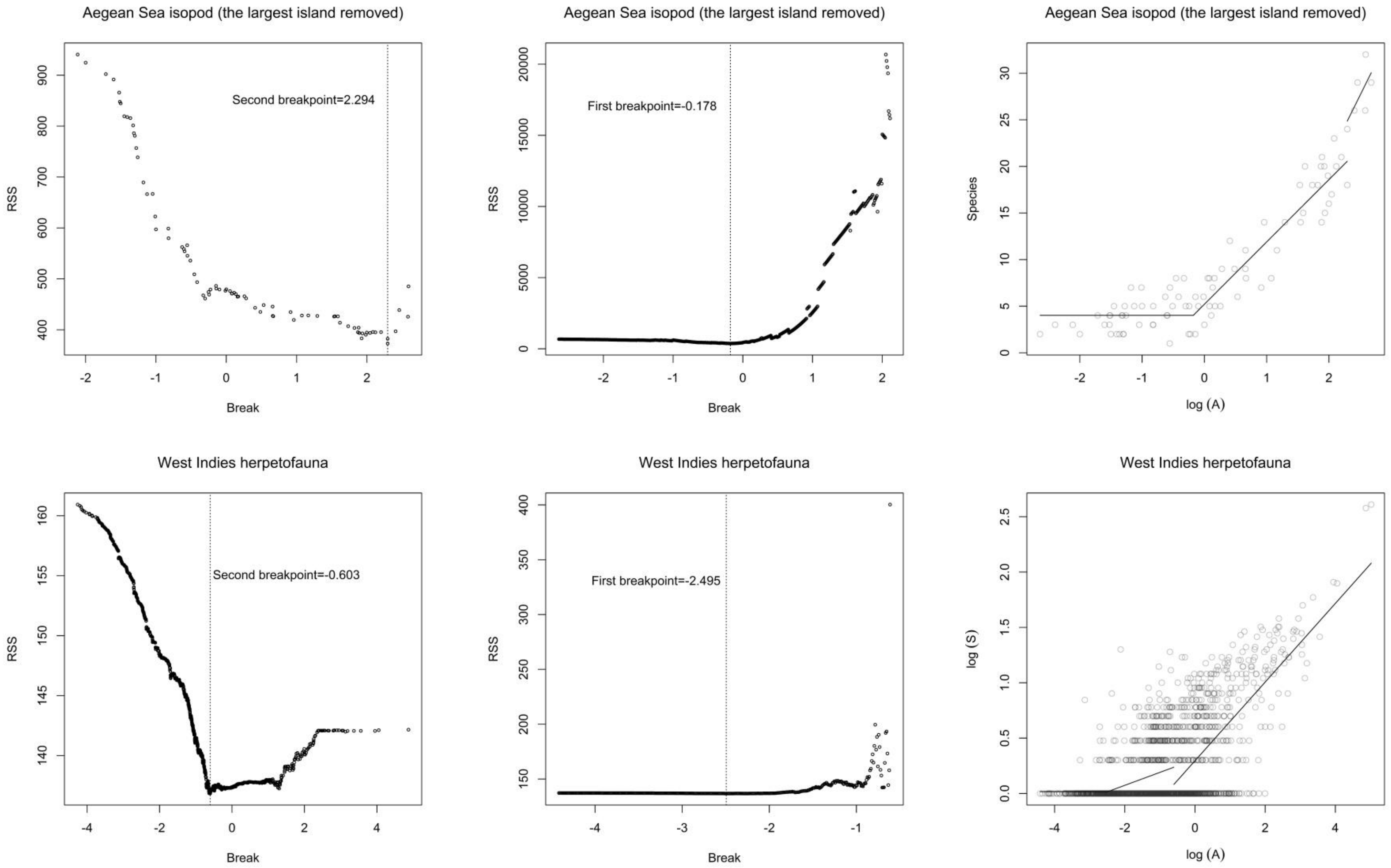
Aegean Sea isopod (the largest island removed)
Second breakpoint=2.294
RSS
Break
Aegean Sea isopod (the largest island removed)
First breakpoint=-0.178
RSS
Break
Aegean Sea isopod (the largest island removed)
Species
log (A)
West Indies herpetofauna
Second breakpoint=-0.603
RSS
Break
West Indies herpetofauna
First breakpoint=-2.495
RSS
Break
West Indies herpetofauna
log (S)
log (A)

Figure S12. Results and the double iterative processes used in Model (12) regression analyses for six sample datasets in accordance with Fig. 2. The first breakpoint (T_1) was obtained prior to the second one (T_2). After T_1 was determined, we run iterative processes of c_1 and T_2 again to look for the c_1 and T_2 that produced the minimum RSS value.

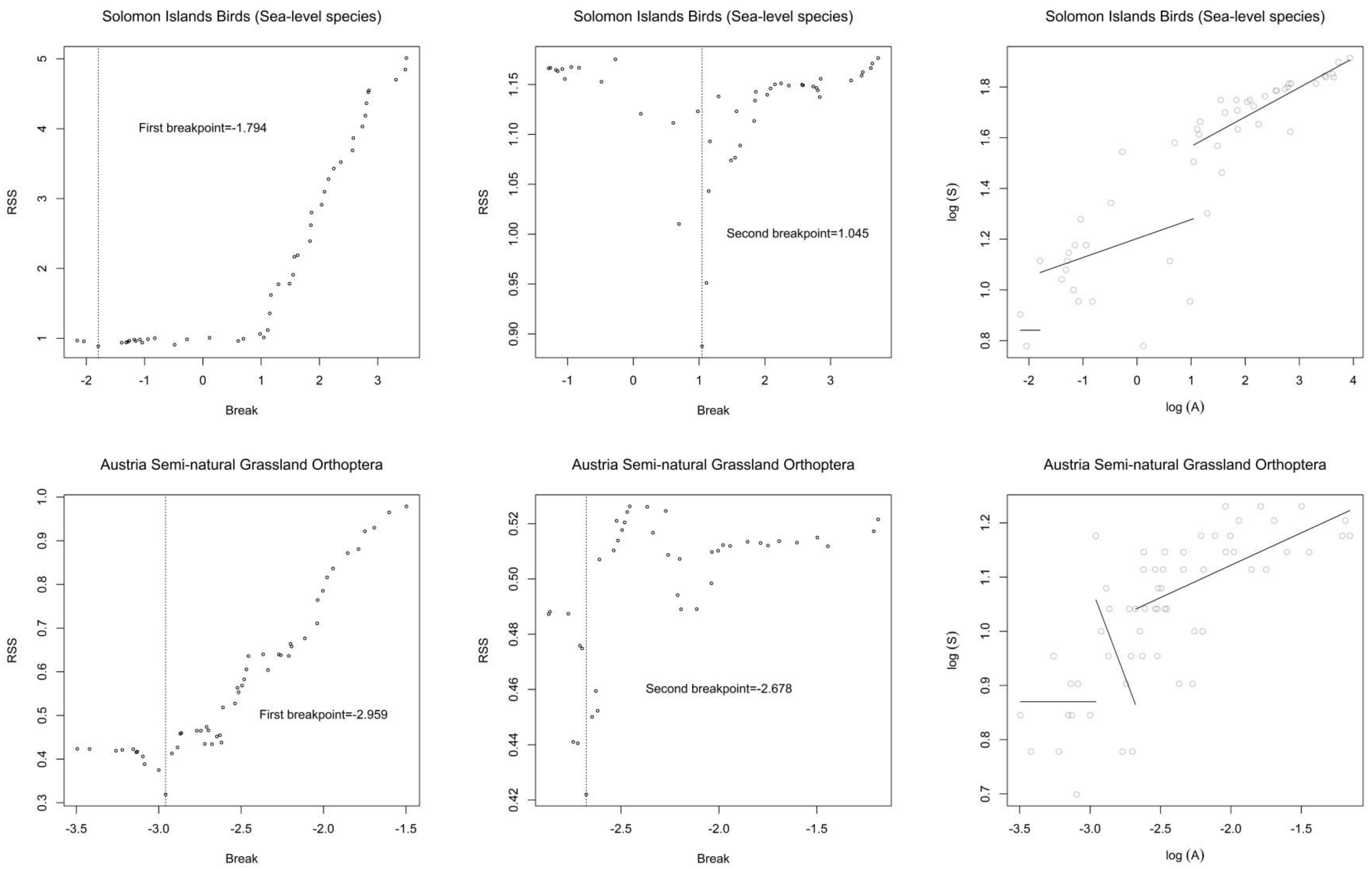

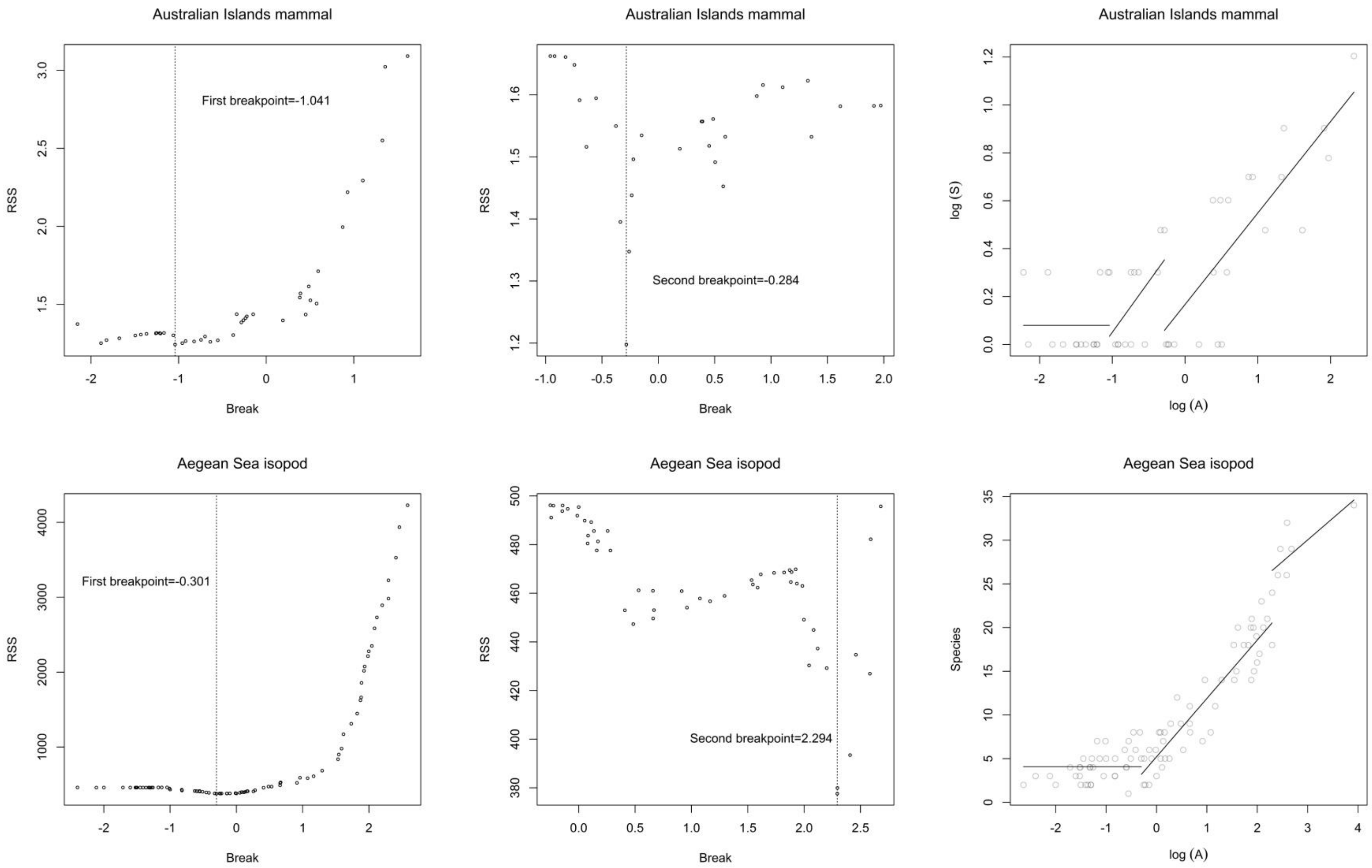
Australian Islands mammal
RSS
Break
First breakpoint=-1.041
Australian Islands mammal
RSS
Break
Second breakpoint=-0.284
Australian Islands mammal
log (S)
log (A)
Aegean Sea isopod
RSS
Break
First breakpoint=-0.301
Aegean Sea isopod
RSS
Break
Second breakpoint=2.294
Aegean Sea isopod
Species
log (A)

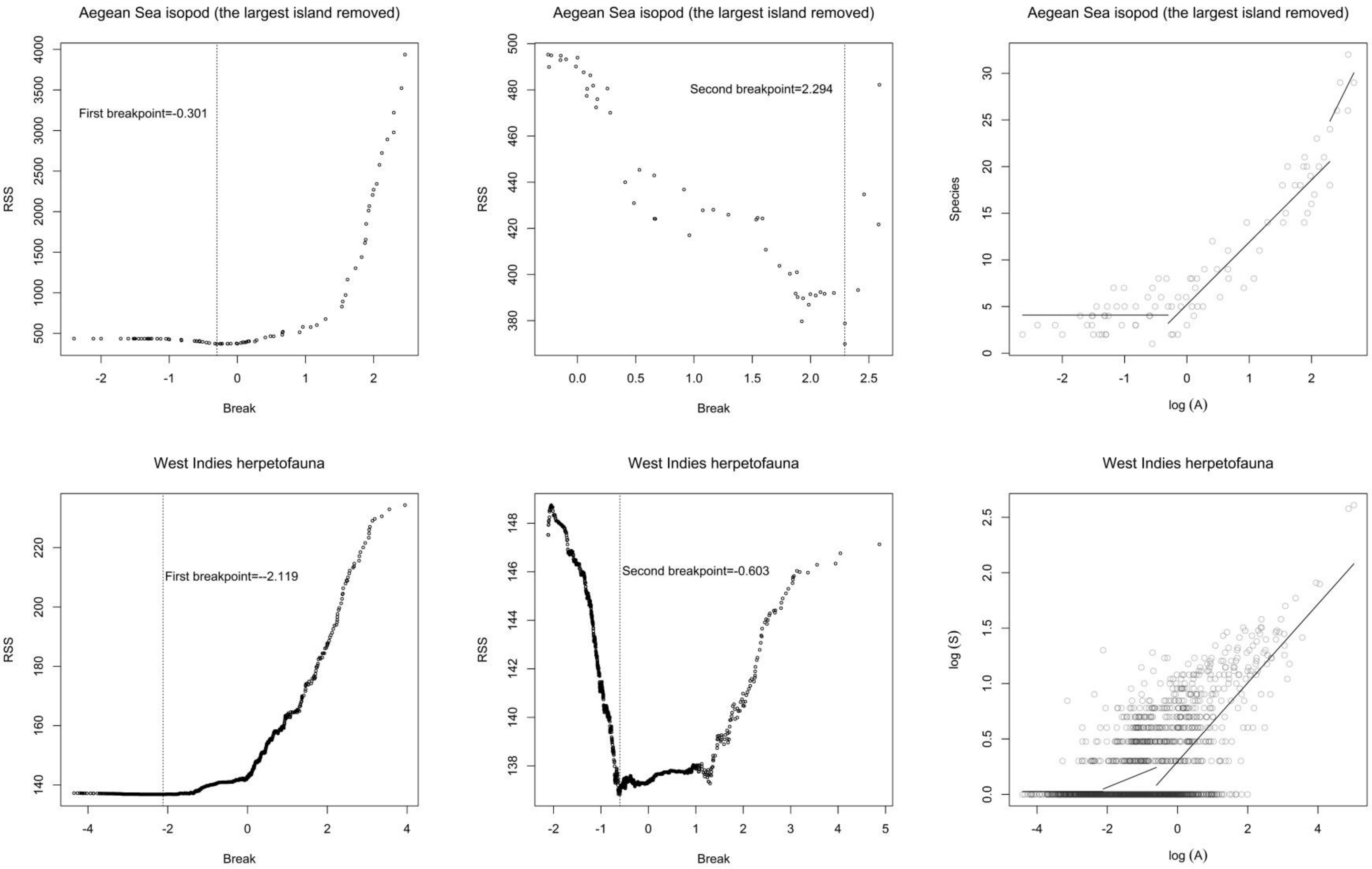
Aegean Sea isopod (the largest island removed)
RSS
Break
First breakpoint=-0.301
Aegean Sea isopod (the largest island removed)
RSS
Break
Second breakpoint=2.294
Aegean Sea isopod (the largest island removed)
Species
log (A)
West Indies herpetofauna
RSS
Break
First breakpoint=--2.119
West Indies herpetofauna
RSS
Break
Second breakpoint=-0.603
West Indies herpetofauna
log (S)
log (A)

Figure S13. Results and the double iterative processes used in Model (13) regression analyses for six sample datasets in accordance with Fig. 2. The first breakpoint (T_1) was obtained prior to the second one (T_2). After T_1 was determined, we run iterative process of T_2 again to look for the T_2 that produced the minimum RSS value.

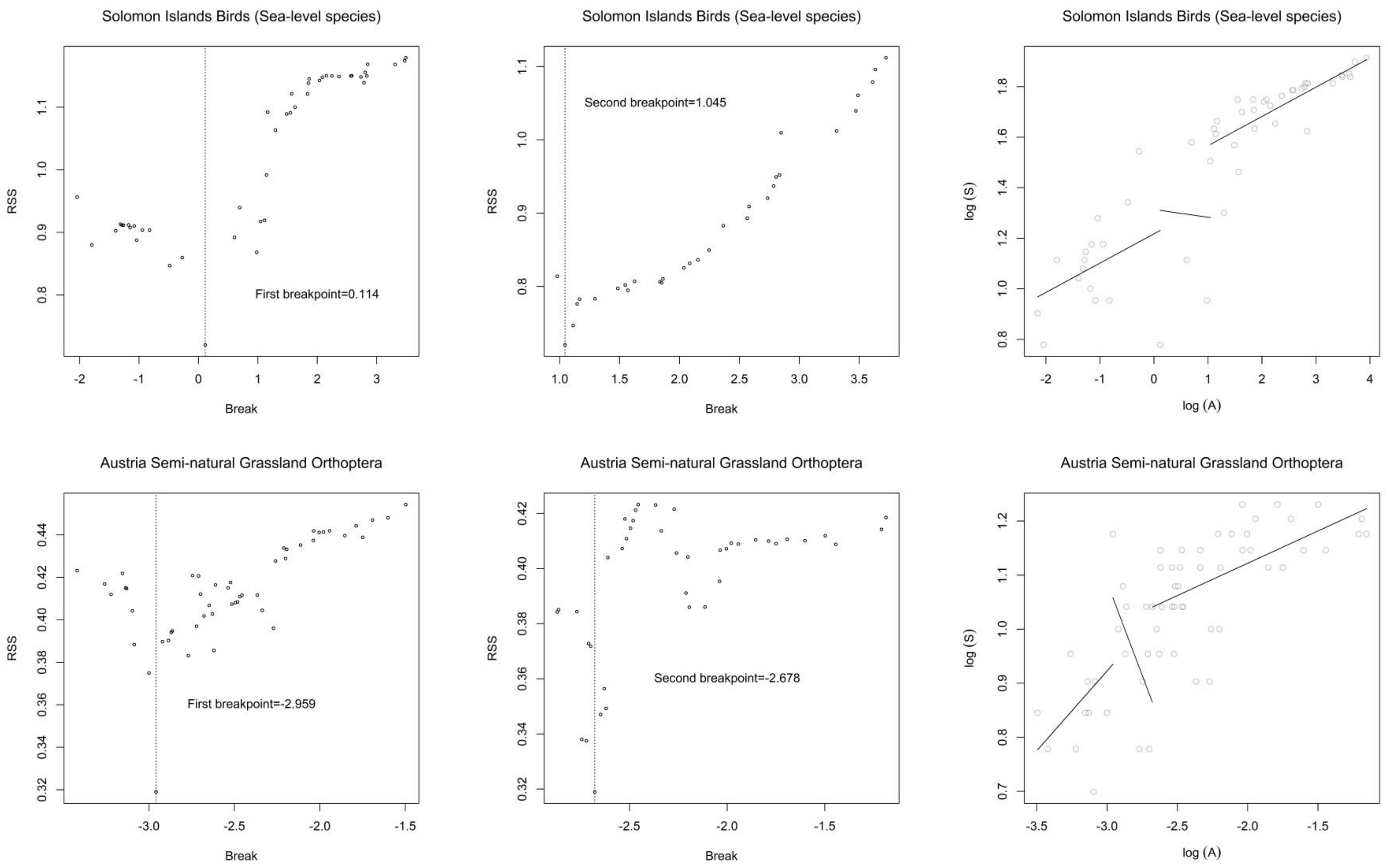

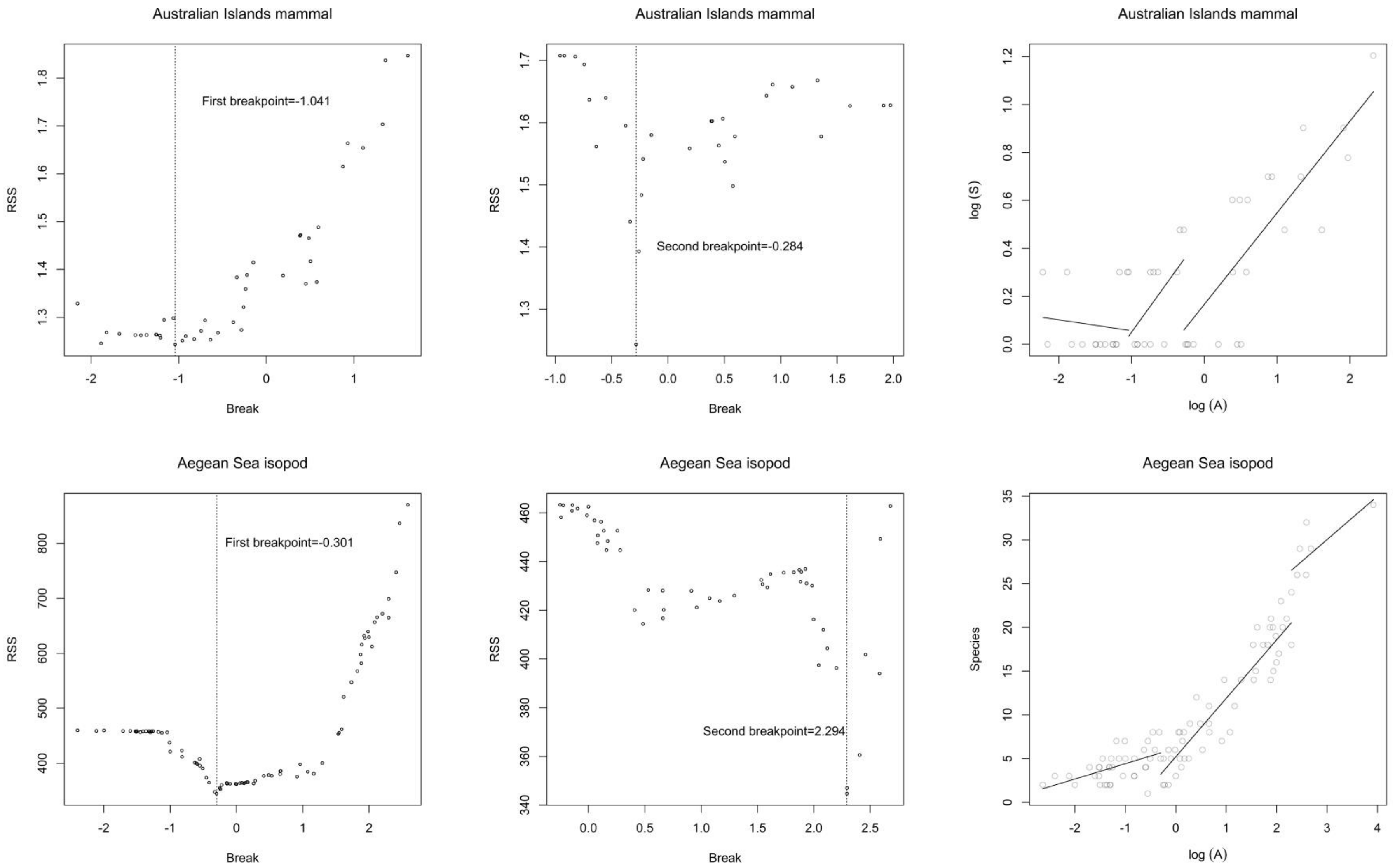
Australian Islands mammal
RSS
Break
First breakpoint=-1.041
Australian Islands mammal
RSS
Break
Second breakpoint=-0.284
Australian Islands mammal
log (S)
log (A)
Aegean Sea isopod
RSS
Break
First breakpoint=-0.301
Aegean Sea isopod
RSS
Break
Second breakpoint=2.294
Aegean Sea isopod
Species
log (A)

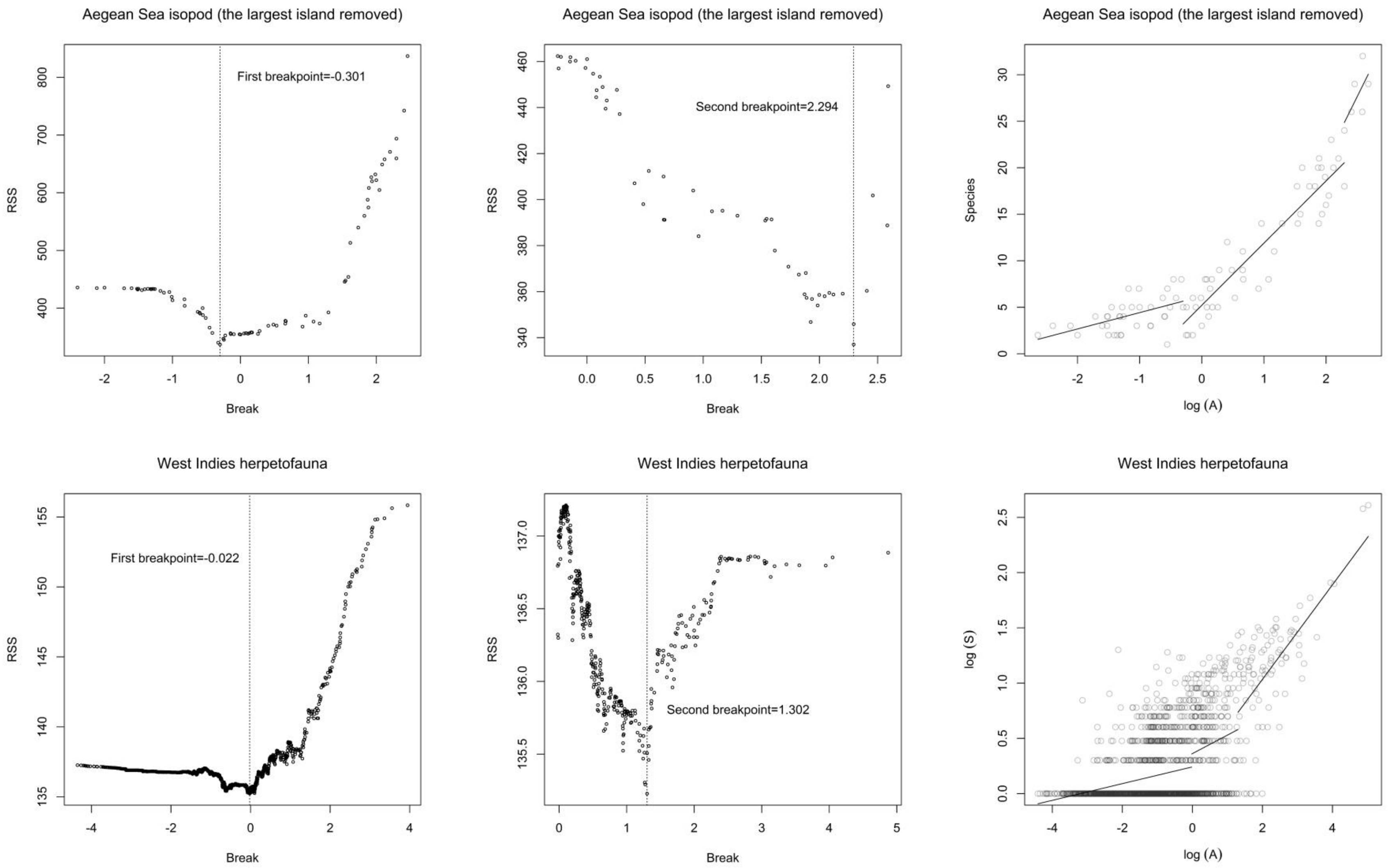

Aegean Sea isopod (the largest island removed)
First breakpoint=-0.301
RSS
Break
Aegean Sea isopod (the largest island removed)
Second breakpoint=2.294
RSS
Break
Aegean Sea isopod (the largest island removed)
Species
log (A)
West Indies herpetofauna
First breakpoint=-0.022
RSS
Break
West Indies herpetofauna
Second breakpoint=1.302
RSS
Break
West Indies herpetofauna
log (S)
log (A)

Figure S14. Results and the double iterative processes used in Model (14) regression analyses for six sample datasets in accordance with Fig. 2. The first breakpoint (T_1) was obtained prior to the second one (T_2). After T_1 was determined, we run iterative processes of T_2 and c_3 again to look for the T_2 and c_3 that produced the minimum RSS value.

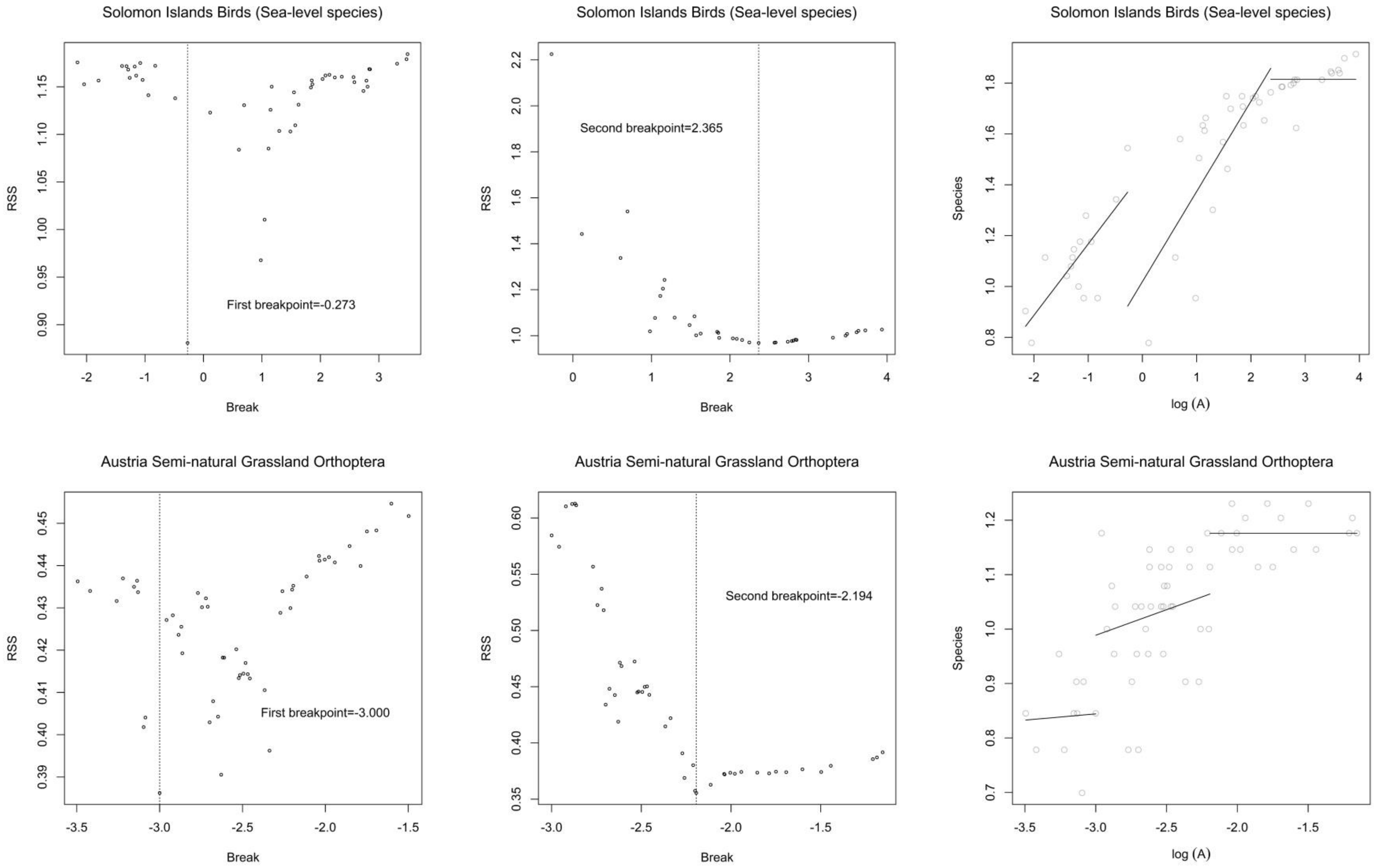

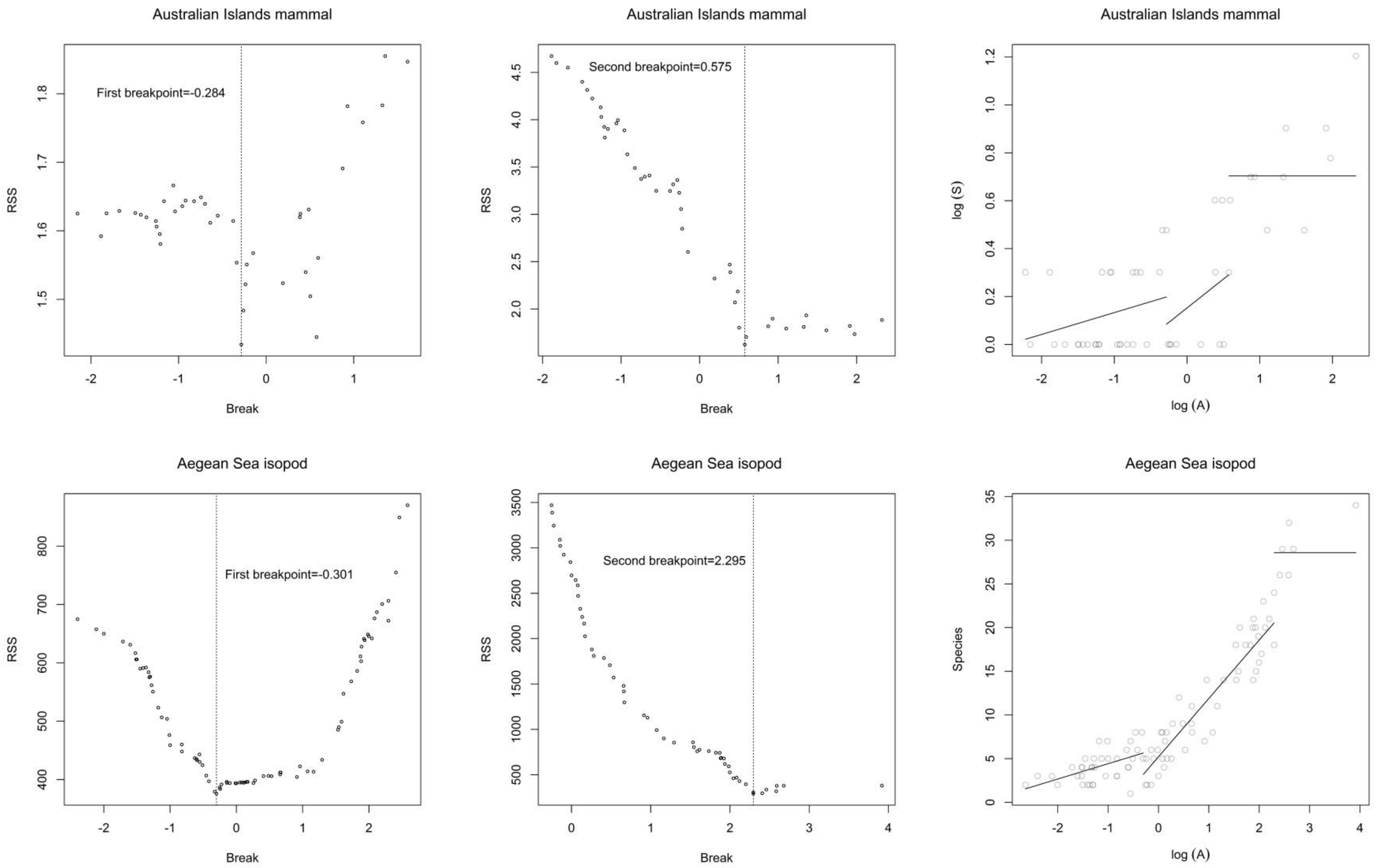
Australian Islands mammal
First breakpoint=-0.284
RSS
Break
Australian Islands mammal
Second breakpoint=0.575
RSS
Break
Australian Islands mammal
log (S)
log (A)
Aegean Sea isopod
First breakpoint=-0.301
RSS
Break
Aegean Sea isopod
Second breakpoint=2.295
RSS
Break
Aegean Sea isopod
Species
log (A)

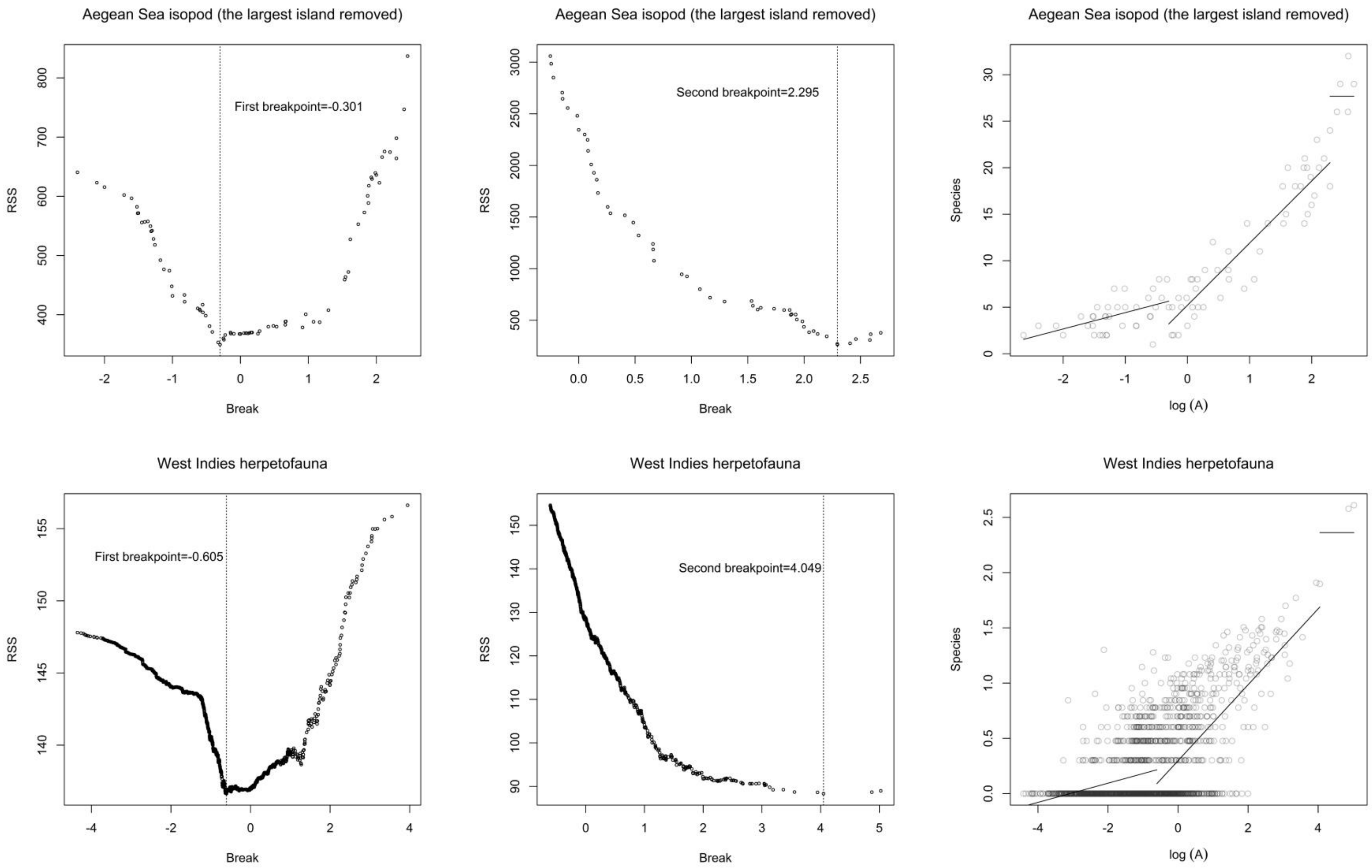
Aegean Sea isopod (the largest island removed)
First breakpoint=-0.301
RSS
Break
Aegean Sea isopod (the largest island removed)
Second breakpoint=2.295
RSS
Break
Aegean Sea isopod (the largest island removed)
Species
log (A)
West Indies herpetofauna
First breakpoint=-0.605
RSS
Break
West Indies herpetofauna
Second breakpoint=4.049
RSS
Break
West Indies herpetofauna
Species
log (A)

Figure S15. Results and the double iterative processes used in Model (15) regression analyses for six sample datasets in accordance with Fig. 2. The second breakpoint (T_2) was obtained prior to the first one (T_1). After T_2 was determined, we run iterative process of T_1 again to look for the T_1 that produced the minimum RSS value.

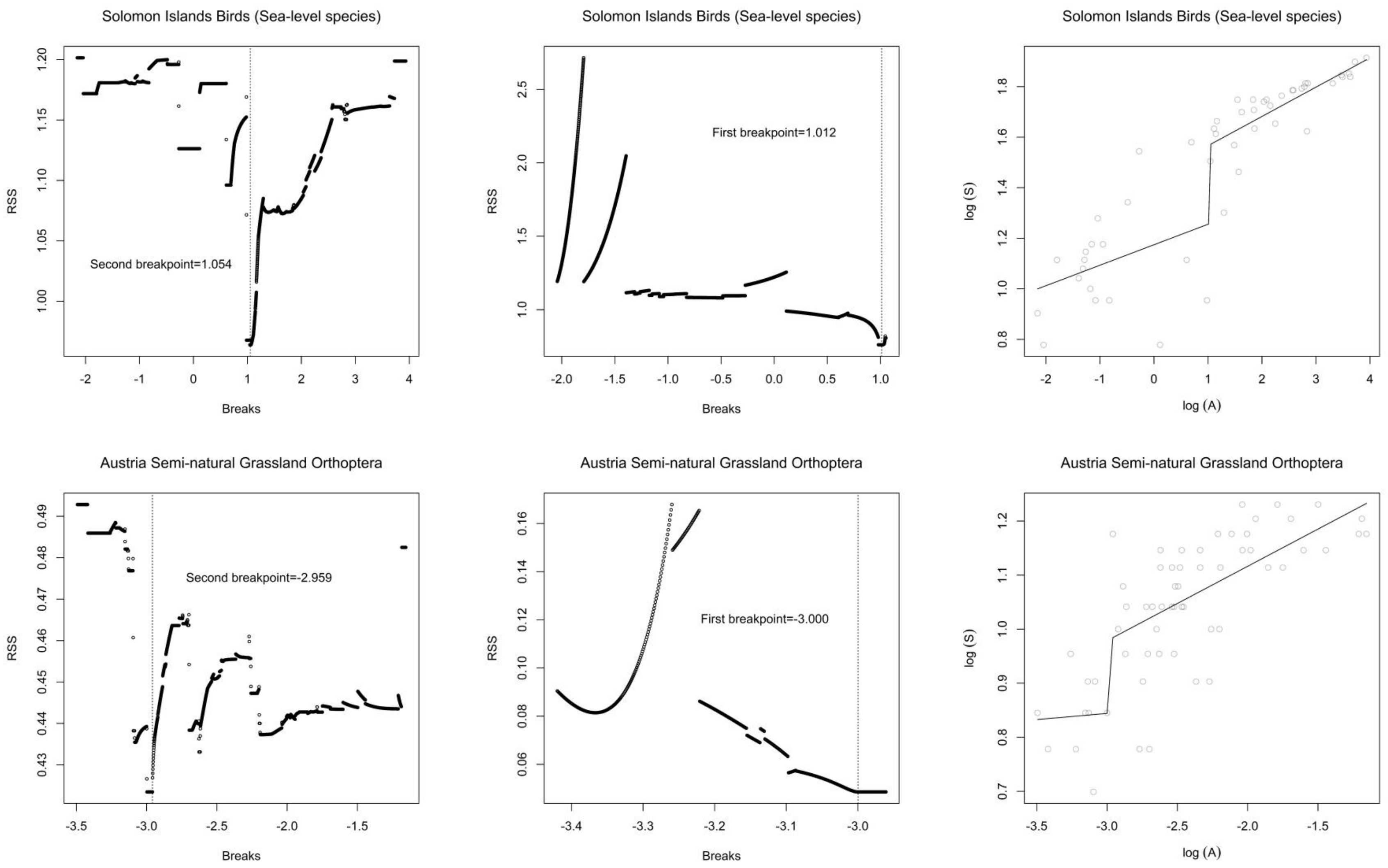

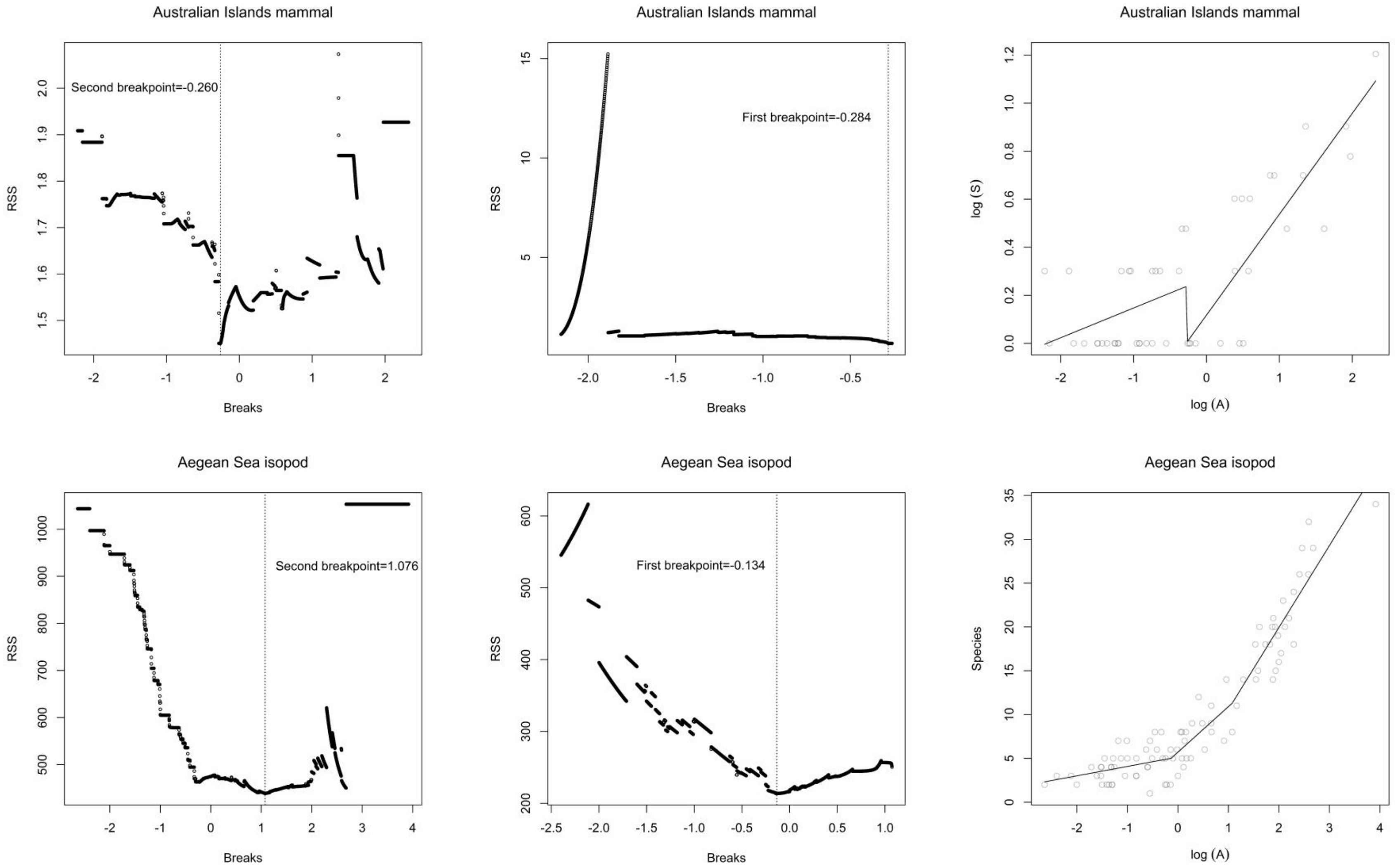
Australian Islands mammal
Second breakpoint=-0.260
RSS
Breaks
Australian Islands mammal
First breakpoint=-0.284
RSS
Breaks
Australian Islands mammal
log (S)
log (A)
Aegean Sea isopod
Second breakpoint=1.076
RSS
Breaks
Aegean Sea isopod
First breakpoint=-0.134
RSS
Breaks
Aegean Sea isopod
Species
log (A)

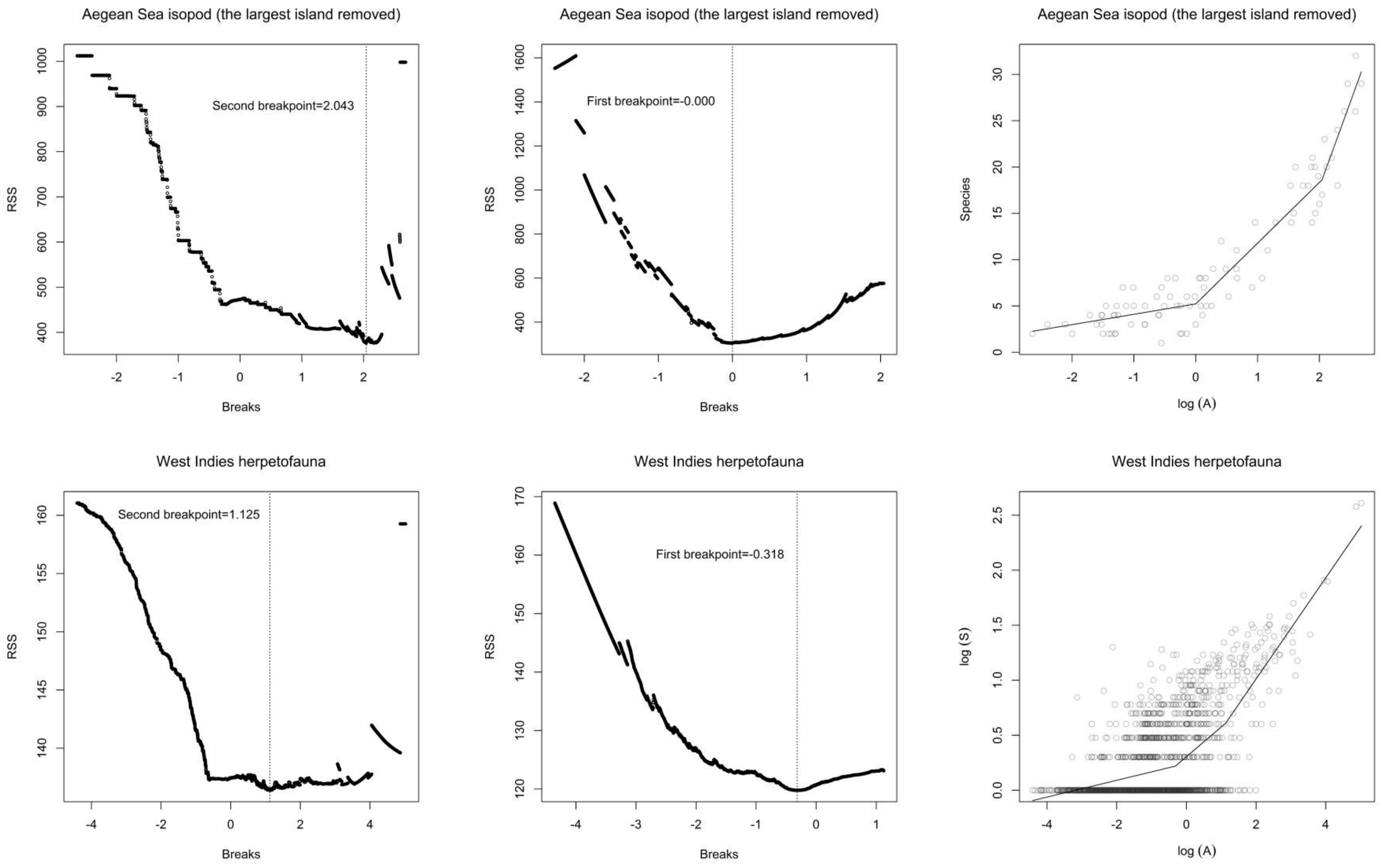
Aegean Sea isopod (the largest island removed)
Second breakpoint=2.043
RSS
Breaks
Aegean Sea isopod (the largest island removed)
First breakpoint=-0.000
RSS
Breaks
Aegean Sea isopod (the largest island removed)
Species
log (A)
West Indies herpetofauna
Second breakpoint=1.125
RSS
Breaks
West Indies herpetofauna
First breakpoint=-0.318
RSS
Breaks
West Indies herpetofauna
log (S)
log (A)
